# Supplementary material for: O-GlcNAcylation of circadian clock protein Bmal1 impairs cognitive function in diabetic mice
Source: EMBO J. 2024 Oct 7;43(22):5667–89. doi: 10.1038/s44318-024-00263-6 (PMC11574178; doi:10.1038/s44318-024-00263-6)
Supplement: Supplementary file 1 — Appendix [file 44318_2024_263_MOESM1_ESM.pdf]

Appendix for:

O-GlcNAcylation of circadian clock protein Bmal1 impairs cognitive function in diabetic mice

Table of contents

Appendix Figure S1 1

Appendix Figure S2 4

Appendix Figure S3 6

Appendix Figure S4 9

Appendix Figure S5 11

Appendix Figure S6 12

Appendix Figure S7 15

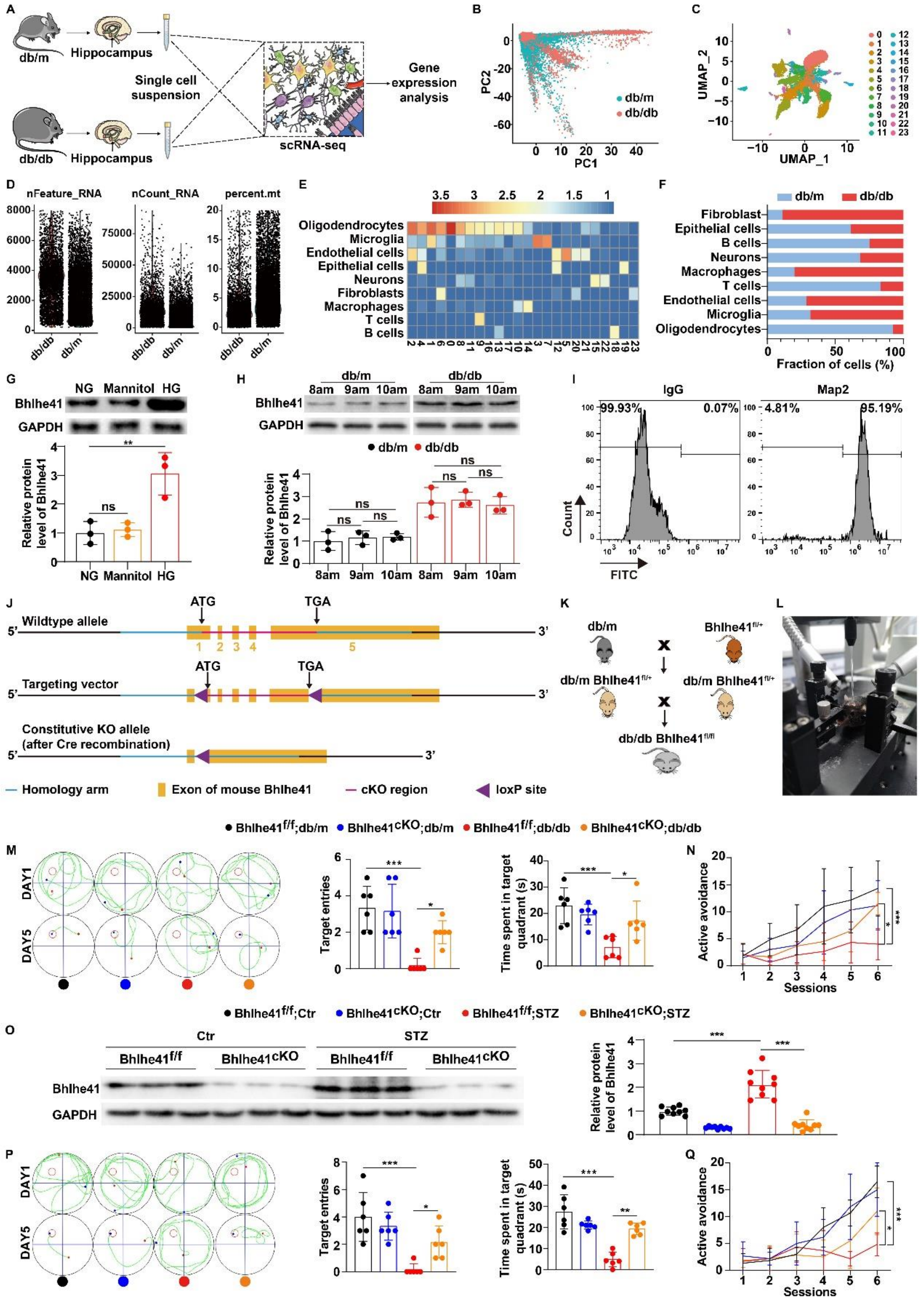

Appendix Figure S1. High Glucose Impairs Cognitive Function through Upregulating Bhlhe41 Expression in Hippocampal Neurons

(A): Workflow depicting collection and processing of hippocampus of db/m and db/db

mice for scRNA-seq.

(B): Principal component analysis (PCA) of single-cell RNA sequencing data from hippocampus of db/m and db/db mice.

(C): Cluster analysis of cells by UMAP.

(D): Number of gene types (nFeature), total gene counts (nCount) and percentage of mitochondrial genes (percent.mt) in the db/m and db/db groups. Each dot represents a hippocampal cell.

(E): 24 cell clusters annotated into 9 cell types using the R “SingleR” package.

(F): Horizontal bar charts showing the relative proportion of each cell type in the hippocampus of db/m (blue) and db/db (red) mice.

(G): Immunoblots and quantification analysis of protein level of Bhlhe41 in primary hippocampal neurons treated with normal glucose (NG, 5.5 mmol/L D-glucose), osmotic control (Mannitol 19.5 mmol/L D-mannitol plus 5.5 mmol/L D-glucose), or high glucose (HG, 25 mmol/L D-glucose) for 48 h (n = 3 replicates). *P* values: 0.9608 (NG vs Mannitol), 0.0058 (NG vs HG).

(H): Immunoblots and quantification analysis of protein level of Bhlhe41 in hippocampal CA1 neurons of db/m and db/db littermates harvested at 8, 9, and 10 am (n = 3 mice per group). *P* values: 0.9964 (db/m, 8am vs 9am), >0.9999 (db/m, 9am vs 10am), 0.9908 (db/m, 8am vs 10am), 0.999 (db/db, 8am vs 9am), >0.9999 (db/db, 9am vs 10am), 0.9989 (db/db, 8am vs 10am).

(I): Purity of isolated hippocampal CA1 neurons by flow cytometry.

(J): Strategy for production of Bhlhe41$^{fl/+}$ mice.

(K): Experimental scheme for generating the db/db Bhlhe41$^{fl/fl}$ mice.

(L): Representative image showing stereotaxic injection.

(M-N): Representative track images during the training trials, target entries and time spent in target quadrant in the probe trial of Morris water maze (M), and active avoidance performance (N) of db/m Bhlhe41$^{fl/fl}$, db/db Bhlhe41$^{fl/fl}$ injected with AAV-CAMKII or AAV-CAMKII-Cre (n = 6 mice per group). *P* values: 0.0002 (M, Target entries, Bhlhe41$^{f/f}$;db/m vs Bhlhe41$^{f/f}$;db/db), 0.0267 (M, Target entries, Bhlhe41$^{f/f}$;db/db vs Bhlhe41^{cKO};db/db), 0.0007 (M, Time spent in target quadrant,

Bhlhe41$^{f/f}$;db/m vs Bhlhe41$^{f/f}$;db/db), 0.0334 (M, Time spent in target quadrant, Bhlhe41$^{f/f}$;db/db vs Bhlhe41^{cKO};db/db); 0.0008 (N, Bhlhe41$^{f/f}$;db/m vs Bhlhe41$^{f/f}$;db/db), 0.0149 (N, Bhlhe41$^{f/f}$;db/db vs Bhlhe41^{cKO};db/db).

(O): Immunoblots and quantification analysis of protein level of Bhlhe41 in the hippocampal CA1 neurons of Ctr Bhlhe41$^{fl/fl}$ or STZ Bhlhe41$^{fl/fl}$ mice injected with AAV-CAMKII or AAV-CAMKII-Cre (n = 9 mice per group). *P* values: <0.0001 (Bhlhe41$^{f/f}$;Ctr vs Bhlhe41$^{f/f}$;STZ), <0.0001 (Bhlhe41$^{f/f}$;STZ vs Bhlhe41^{cKO};STZ).

(P-Q): Representative track images during the training trials, target entries and time spent in target quadrant in the probe trial of Morris water maze (P), and active avoidance performance (Q) of Ctr Bhlhe41$^{fl/fl}$, STZ Bhlhe41$^{fl/fl}$ mice injected with AAV-CAMKII or AAV-CAMKII-Cre (n = 6 mice per group). *P* values: 0.0001 (P, Target entries, Bhlhe41$^{f/f}$;Ctr vs Bhlhe41$^{f/f}$;STZ), 0.0426 (P, Target entries, Bhlhe41$^{f/f}$;STZ vs Bhlhe41^{cKO};STZ), 0.0007 (P, Time spent in target quadrant, Bhlhe41$^{f/f}$;Ctr vs Bhlhe41$^{f/f}$;STZ), 0.0334 (P, Time spent in target quadrant, Bhlhe41$^{f/f}$;STZ vs Bhlhe41^{cKO};STZ); 0.0002 (Q, Bhlhe41$^{f/f}$;Ctr vs Bhlhe41$^{f/f}$;STZ), 0.0467 (Q, Bhlhe41$^{f/f}$;STZ vs Bhlhe41^{cKO};STZ);

Data are means ± SEM. ns, not significant, *P < 0.05, **P < 0.01, ***P < 0.001. One-way ANOVA followed by Tukey's test (G), Two-way ANOVA followed by Tukey's test (H and M-Q).

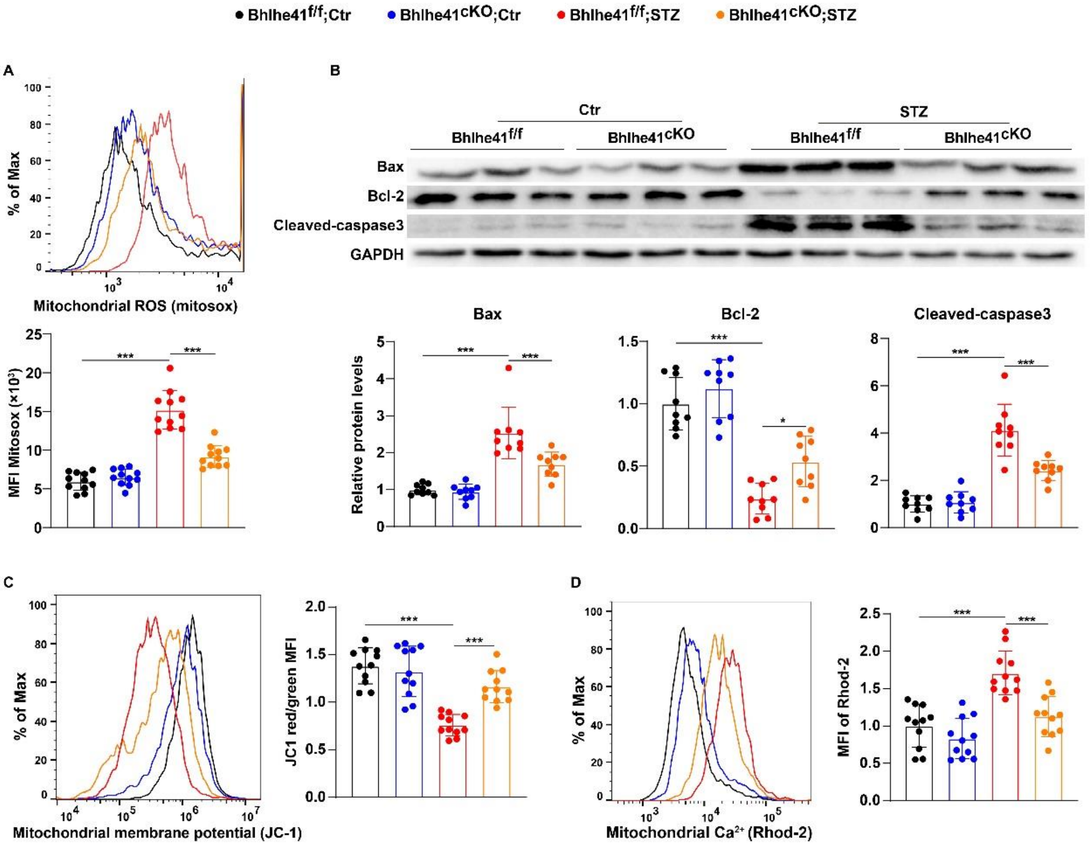

Appendix Figure S2. Bhlhe41 Contributes to Mitochondrial Calcium Overload and Oxidative Damage in Hippocampal Neurons under Diabetic Conditions

(A): Flow cytometry analysis of mtROS levels (n = 11 mice per group) in the hippocampal CA1 neurons of Ctr Bhlhe41$^{fl/fl}$ or STZ Bhlhe41$^{fl/fl}$ mice injected with AAV-CAMKII or AAV-CAMKII-Cre. *P* values: <0.0001 (Bhlhe41$^{f/f}$;Ctr vs Bhlhe41$^{f/f}$;STZ), <0.0001 (Bhlhe41$^{f/f}$;STZ vs Bhlhe41^{cKO};STZ).

(B): Immunoblots and quantification analysis of protein levels of Bax, Bcl-2 and Cleaved-caspase3 (n = 9 mice per group) in the hippocampal CA1 neurons of Ctr Bhlhe41$^{fl/fl}$ or STZ Bhlhe41$^{fl/fl}$ mice injected with AAV-CAMKII or AAV-CAMKII-Cre. *P* values: <0.0001 (Bax, Bhlhe41$^{f/f}$;Ctr vs Bhlhe41$^{f/f}$;STZ), <0.0001 (Bax, Bhlhe41$^{f/f}$;STZ vs Bhlhe41^{cKO};STZ); <0.0001 (Bcl-2, Bhlhe41$^{f/f}$;Ctr vs Bhlhe41$^{f/f}$;STZ), 0.0442 (Bcl-2, Bhlhe41$^{f/f}$;STZ vs Bhlhe41^{cKO};STZ); <0.0001 (Cleaved-caspase3, Bhlhe41$^{f/f}$;Ctr vs Bhlhe41$^{f/f}$;STZ), <0.0001 (Cleaved-caspase3, Bhlhe41$^{f/f}$;STZ vs Bhlhe41^{cKO};STZ).

(C-D): Flow cytometry analysis of MMP (C, n = 11 mice per group) and mitochondrial calcium levels (D, n = 11 mice per group) in the hippocampal CA1 neurons of Ctr $Bhlhe41^{fl/fl}$ or STZ $Bhlhe41^{fl/fl}$ mice injected with AAV-CAMKII or AAV-CAMKII-Cre. *P* values: <0.0001 (C, $Bhlhe41^{f/f}$;Ctr vs $Bhlhe41^{f/f}$;STZ), <0.0001 (C, $Bhlhe41^{f/f}$;STZ vs $Bhlhe41^{cKO}$;STZ); <0.0001 (D, $Bhlhe41^{f/f}$;Ctr vs $Bhlhe41^{f/f}$;STZ), 0.0001 (D, $Bhlhe41^{f/f}$;STZ vs $Bhlhe41^{cKO}$;STZ)

Data are means ± SEM. *P < 0.05, ***P < 0.001. Two-way ANOVA followed by Tukey's test (A-D).

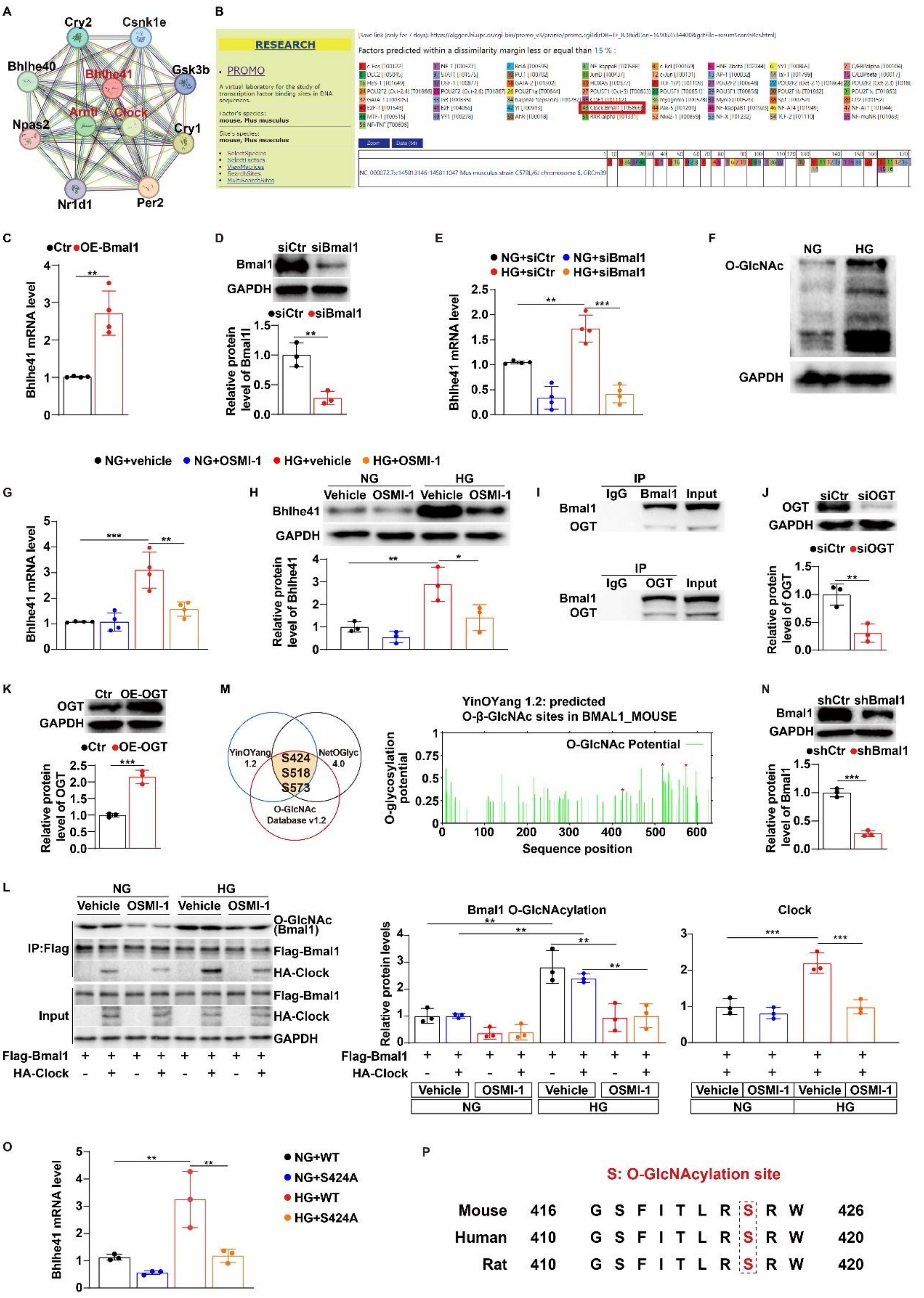

Appendix Figure S3. Increasing O-GlcNAcylation of Bmal1 at S424 Contributes to High Glucose-Induced Upregulation of Bhlhe41 Expression in Hippocampal Neurons

(A): Protein-interaction network plotted using the STRING database.

(B): Potential transcription factors of Bhlhe41 were predicted in ALGGEN website.

(C): mRNA levels of Bhlhe41 in primary hippocampal neurons transfected with Bmal1 overexpression plasmid or control plasmid (n = 4 replicates). *P* value: 0.0012.

(D): Immunoblots and quantification analysis of protein level of Bmal1 in primary hippocampal neurons treated with Bmal1 siRNA or control siRNA (n = 3 replicates). *P* value: 0.0056.

(E): mRNA levels of Bhlhe41 in primary hippocampal neurons treated with Bmal1 siRNA or control siRNA in the presence of normal glucose (NG, 5.5 mmol/L D-glucose) or high glucose (HG, 25 mmol/L D-glucose) (n = 4 replicates). *P* values: 0.0021 (NG+siCtr vs HG+siCtr), <0.0001 (HG+siCtr vs HG+siBmal1).

(F): Immunoblotting analysis of global O-GlcNAcylation levels in primary hippocampal neurons treated with normal glucose (NG, 5.5 mmol/L D-glucose) or high glucose (HG, 25 mmol/L D-glucose) for 48 hours (n = 3 replicates).

(G-H): mRNA level (G, n = 4 replicates), immunoblots and quantification analysis of protein level of Bhlhe41 (H, n = 3 replicates) in primary hippocampal neurons treated with normal glucose (NG, 5.5 mmol/L D-glucose) or high glucose (HG, 25 mmol/L D-glucose) in the presence or absence of OGT inhibitors OSMI-1 (20 μM). *P* values: <0.0001 (G, NG+vehicle vs HG+vehicle), 0.0012 (G, HG+vehicle vs HG+OSMI-1), 0.0075 (H, NG+vehicle vs HG+vehicle), 0.0288 (H, HG+vehicle vs HG+OSMI-1).

(I): Reciprocal endogenous immunoprecipitation (IP) was performed to detect interactions between OGT and Bmal1 in primary hippocampal neurons.

(J): Immunoblots and quantification analysis of protein level of OGT in primary hippocampal neurons treated with OGT siRNA or control siRNA (n = 3 replicates). *P* value: 0.0091.

(K): Immunoblots and quantification analysis of protein level of OGT in primary hippocampal neurons transfected with OGT overexpression plasmid or control plasmid (n = 3 replicates). *P* value: 0.0007.

(L): HEK293T cells were transfected with Flag-Bmal1 in the presence or absence of HA-Clock as indicated. After treated with normal glucose (NG, 5.5 mmol/L D-glucose) or high glucose (HG, 25mmol/L D-glucose) in the presence of vehicle or OSMI-1

(20μM), cells were lysed for precipitated proteins detection by immunoprecipitation assay with anti-Flag antibodies and western blot with indicated antibodies (n = 3 replicates). *P* values: 0.004 (Bmal1O-GlcNAcylation, NG+vehicle vs HG+vehicle), 0.0013 (Bmal1O-GlcNAcylation, NG+vehicle+HA-Clock vs HG+vehicle+HA-Clock), 0.0032 (Bmal1O-GlcNAcylation, HG+vehicle vs HG+OSMI-1), 0.0013 (Bmal1O-GlcNAcylation, HG+vehicle+HA-Clock vs HG+OSMI-1+HA-Clock); 0.0007 (Clock, NG+vehicle vs HG+vehicle), 0.0006 (Clock, HG+vehicle vs HG+OSMI-1).

(M): O-glycosylation sites were predicted using YinOYang 1.2, NetOGlyc 4.0 and the O-GlcNAc Database (v1.2).

(N): Immunoblots and quantification analysis of protein level of Bmal1 in primary hippocampal neurons transduced with shCtr or shBmal1 (n = 3 replicates). *P* value: 0.0002.

(O): mRNA levels of Bhlhe41 in primary hippocampal neurons with endogenous Bmal1 stably silenced and expressing either wild-type (WT) Bmal1 or S424A Bmal1, treated with normal glucose (NG, 5.5 mmol/L D-glucose) or high glucose (HG, 25 mmol/L D-glucose) (n = 3 replicates). *P* values: 0.0054 (NG+WT vs HG+WT), 0.0064 (HG+WT vs HG+S424A).

(P): Cross-species sequence alignment of Bmal1.

Data are means ± SEM. *P < 0.05, **P < 0.01, ***P < 0.001. Two-tailed Student's unpaired t test analysis (C-D, J-K and N), Two-way ANOVA followed by Tukey's test (E, G-H, L and O).

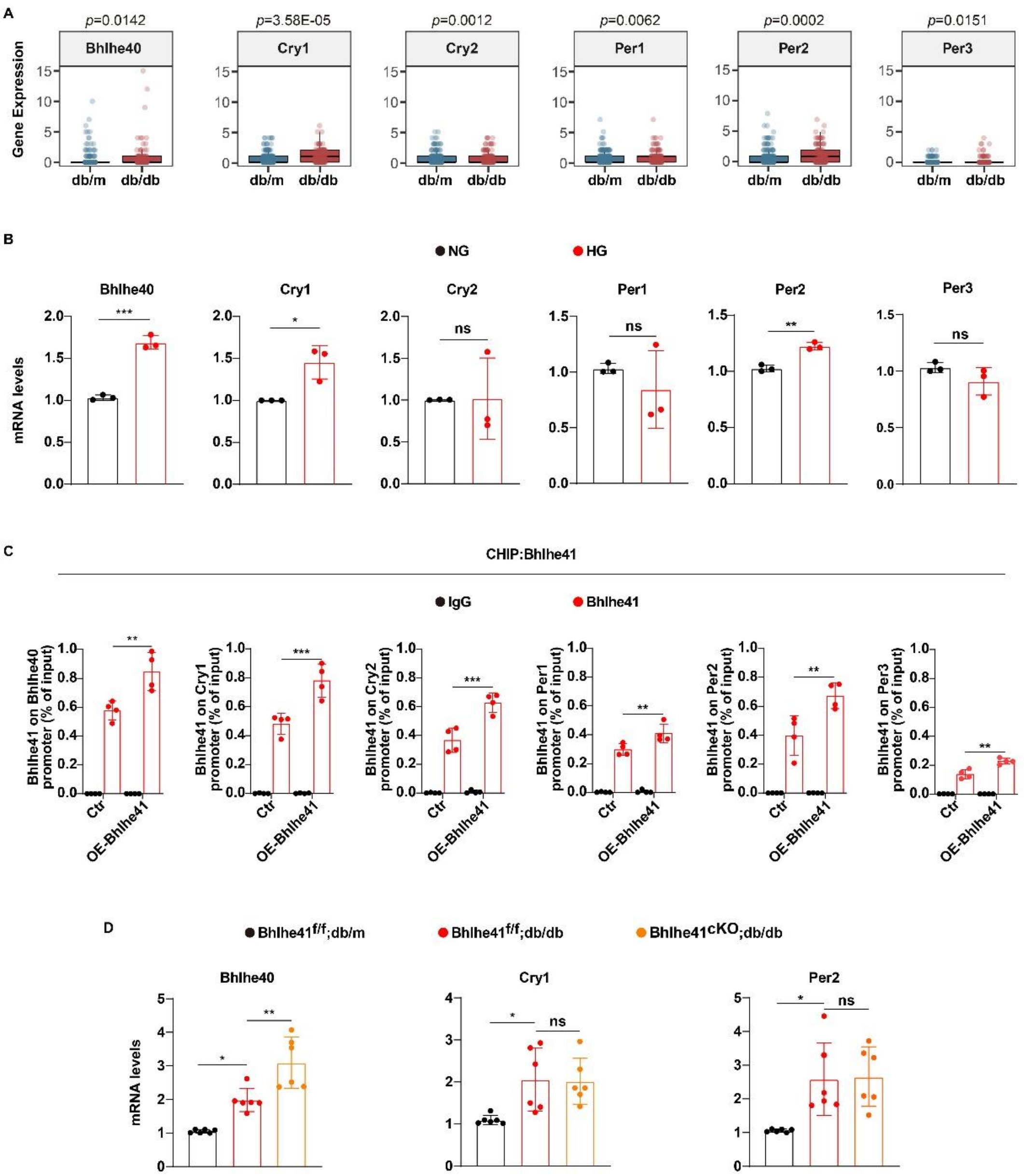

Appendix Figure S4. High Glucose Increased the Expression of Clock Controlled Genes Regulated by Bmal1/Clock Heterodimer in Hippocampal Neurons

(A): Box plots showing gene expression of Bhlhe40, Cryptochrome (Cry1, Cry2) and Period (Per1, Per2, Per3) in hippocampal neurons of db/m and db/db mice identified by scRNA-seq. Boxes represent the first and third quartiles, with the medians indicated by the central lines. Whiskers extend to the lowest and highest values within 1.5 times the interquartile range (n=1 mouse per group).

(B): mRNA levels of Bhlhe40, Cryptochrome (Cry1, Cry2) and Period (Per1, Per2, Per3) in primary hippocampal neurons treated with normal glucose (NG, 5.5 mmol/L D-

glucose) or high glucose (HG, 25 mmol/L D-glucose) for 48 h (n = 3 replicates). *P* values: 0.0002 (Bhlhe40), 0.0169 (Cry1), 0.9641 (Cry2), 0.4055 (Per1), 0.0015 (Per2), 0.1773 (Per3).

(C): Binding of Bhlhe41 to the Bhlhe40, Cryptochrome (Cry1, Cry2) and Period (Per1, Per2, Per3) promoter in primary hippocampal neurons transfected with control plasmid or a plasmid overexpressing Bhlhe41 for 48h. IgG was used as negative CHIP control (n = 3 replicates). *P* values: 0.0012 (Bhlhe40), 0.0002 (Cry1), <0.0001 (Cry2), 0.007 (Per1), 0.0024 (Per2), 0.0030 (Per3).

(D): mRNA levels of Bhlhe40, Cry1 and Per2 in hippocampal CA1 neurons of Bhlhe41$^{fl/fl}$;db/m, Bhlhe41$^{fl/fl}$;db/db and Bhlhe41^{cKO};db/db mice (n = 6 mice per group). *P* values: 0.0117 (Bhlhe40, Bhlhe41$^{fl/fl}$;db/m vs Bhlhe41$^{fl/fl}$;db/db), 0.0029 (Bhlhe41$^{fl/fl}$;db/db vs Bhlhe41^{cKO};db/db) 0.0193 (Cry1, Bhlhe41$^{fl/fl}$;db/m vs Bhlhe41$^{fl/fl}$;db/db), 0.9902 (Cry1, Bhlhe41$^{fl/fl}$;db/db vs Bhlhe41^{cKO};db/db), 0.0128 (Per2, Bhlhe41$^{fl/fl}$;db/m vs Bhlhe41$^{fl/fl}$;db/db), 0.9866 (Per2, Bhlhe41$^{fl/fl}$;db/db vs Bhlhe41^{cKO};db/db).

Data are means ± SEM. ns, not significant, *P < 0.05, **P < 0.01, ***P < 0.001. Wilcoxon rank-sum test (A), Two-tailed Student's unpaired t test analysis (B), One-way ANOVA followed by Tukey's test (D), Two-way ANOVA followed by Tukey's test (C).

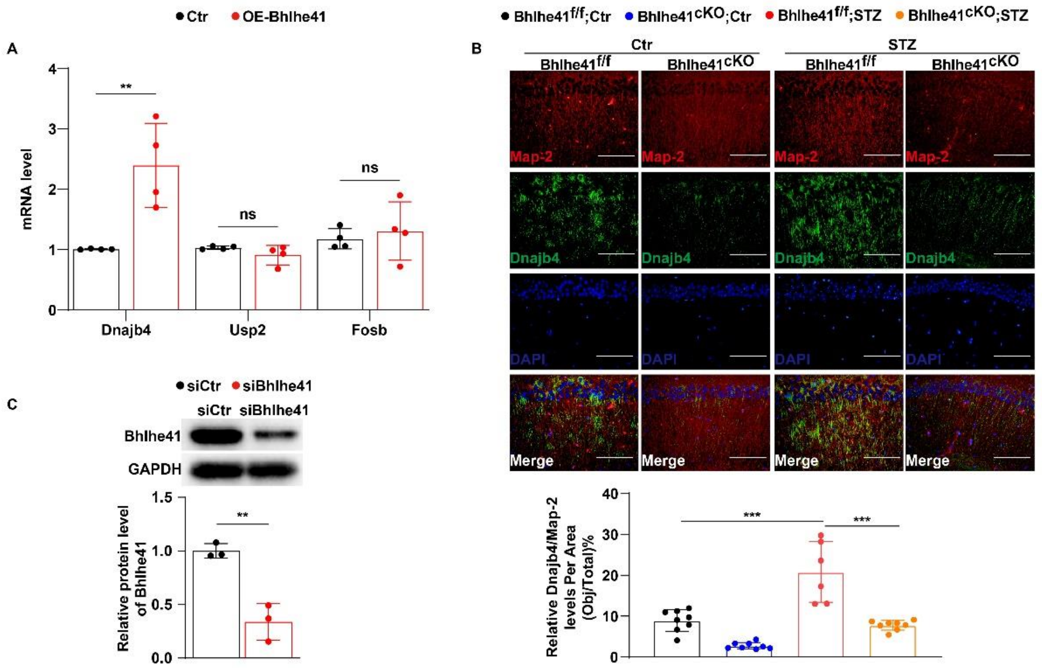

Appendix Figure S5. High Glucose Increases Dnajb4 Expression by Upregulating Bhlhe41 in Hippocampal Neurons

(A): mRNA levels of Dnajb4, Usp2, Fosb in primary hippocampal neurons transfected with control plasmid or a plasmid overexpressing Bhlhe41 for 48h (n = 4 replicates). *P* values: 0.0072 (Dnajb4), 0.1828 (Usp2), 0.6274 (Fosb).

(B): Representative images and quantifications showing IF staining of Dnajb4 (green) and MAP-2 (red) in the hippocampal CA1 neurons of Ctr Bhlhe41$^{fl/fl}$ or STZ Bhlhe41$^{fl/fl}$ mice injected with AAV-CAMKII or AAV-CAMKII-Cre (n = 6-8 mice per group), Scale. bar: 100 μm. *P* values: <0.0001 (Bhlhe41$^{f/f}$;Ctr vs Bhlhe41$^{f/f}$;STZ), <0.0001 (Bhlhe41$^{f/f}$;STZ vs Bhlhe41^{cKO};STZ).

(C): A representative western blot (n = 3 blots in total) and quantification of Bhlhe41 expression in primary hippocampal neurons treated with Bhlhe41 siRNA or control siRNA. *P* value: 0.0033.

Data are means ± SEM. ns, not significant, **P < 0.01, ***P < 0.001. Two-tailed Student's unpaired t test analysis (A and C), Two-way ANOVA followed by Tukey's test (B).

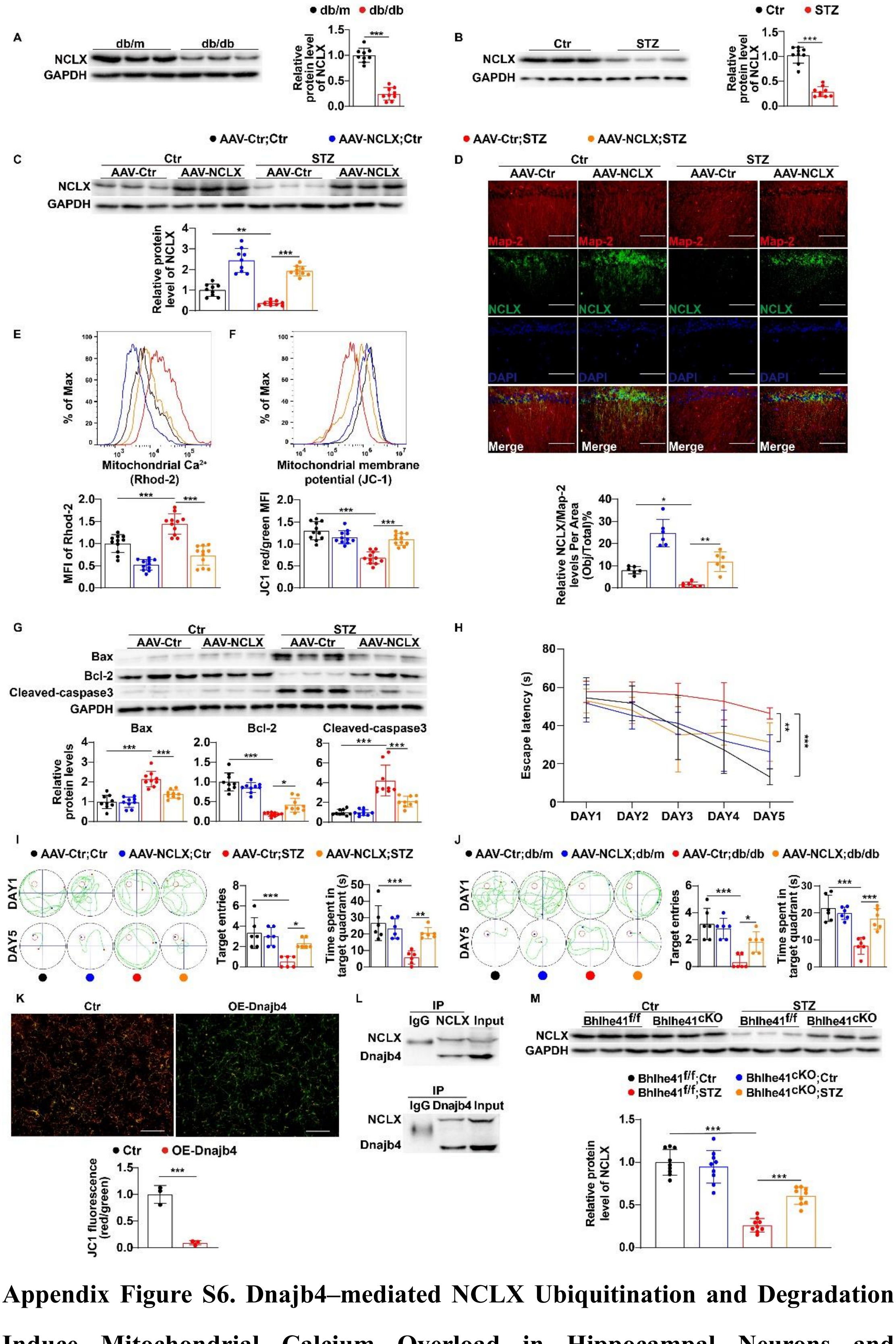

Appendix Figure S6. Dnajb4–mediated NCLX Ubiquitination and Degradation Induce Mitochondrial Calcium Overload in Hippocampal Neurons and Subsequently Lead to Mitochondrial Dysfunction and Neuronal Damage

(A-B): Immunoblots and quantification analysis of protein level of NCLX in the

hippocampal CA1 neurons of db/m and db/db mice (A, n = 9 mice per group), Ctr and STZ mice (B, n = 9 mice per group). *P* values: <0.0001 (A), <0.0001 (B).

(C): Immunoblots and quantification analysis of protein level of NCLX in the hippocampal CA1 neurons of Ctr or STZ mice injected with AAV-CAMKII or AAV-CAMKII-NCLX (n = 9 mice per group). *P* values: 0.0026 (AAV-Ctr;Ctr vs AAV-Ctr;STZ), <0.0001 (AAV-Ctr;STZ vs AAV-NCLX;STZ).

(D): Representative images and quantifications showing IF staining of NCLX (green) and MAP-2 (red) in the hippocampal CA1 neurons of Ctr or STZ mice injected with AAV-CAMKII or AAV-CAMKII-NCLX (n = 6 mice per group). Scale bar: 100 μm. *P* values: 0.0455 (AAV-Ctr;Ctr vs AAV-Ctr;STZ), 0.0011 (AAV-Ctr;STZ vs AAV-NCLX;STZ).

(E-F): Flow cytometry analysis of mitochondrial calcium levels (E, n = 11 mice per group), MMP (F, n = 11 mice per group) in the hippocampal CA1 neurons of Ctr or STZ mice injected with AAV-CAMKII or AAV-CAMKII-NCLX. *P* values: <0.0001 (E, AAV-Ctr;Ctr vs AAV-Ctr;STZ), <0.0001 (E, AAV-Ctr;STZ vs AAV-NCLX;STZ); <0.0001 (F, AAV-Ctr;Ctr vs AAV-Ctr;STZ), <0.0001 (F, AAV-Ctr;STZ vs AAV-NCLX;STZ).

(G): Immunoblots and quantification analysis of protein levels of Bax, Bcl-2 and Cleaved-caspase3 in the hippocampal CA1 neurons of Ctr or STZ mice injected with AAV-CAMKII or AAV-CAMKII-NCLX (n = 9 mice per group). *P* values: <0.0001 (Bax, AAV-Ctr;Ctr vs AAV-Ctr;STZ), <0.0001 (Bax, AAV-Ctr;STZ vs AAV-NCLX;STZ); <0.0001 (Bcl-2, AAV-Ctr;Ctr vs AAV-Ctr;STZ), 0.0173 (Bcl-2, AAV-Ctr;STZ vs AAV-NCLX;STZ); <0.0001 (Cleaved-caspase3, AAV-Ctr;Ctr vs AAV-Ctr;STZ), <0.0001 (Cleaved-caspase3, AAV-Ctr;STZ vs AAV-NCLX;STZ).

(H): Escape latency to the platform during the training trial in a Morris water maze of Ctr or STZ mice injected with AAV-CAMKII or AAV-CAMKII-NCLX (n = 6 mice per group). *P* values: <0.0001 (AAV-Ctr;Ctr vs AAV-Ctr;STZ), 0.0083 (AAV-Ctr;STZ vs AAV-NCLX;STZ).

(I-J): Representative track images during the training trials in a Morris water maze, target entries and time spent in target quadrant in the probe trial of Morris water maze

of Ctr, STZ (I), db/m, db/db (J) mice injected with AAV-CAMKII or AAV-CAMKII-NCLX (n = 6 mice per group). *P* values: 0.0003 (I, Target entries, AAV-Ctr;Ctr vs AAV-Ctr;STZ), 0.0161 (I, Target entries, AAV-Ctr;STZ vs AAV-NCLX;STZ), 0.0001 (I, Time spent in target quadrant, AAV-Ctr;Ctr vs AAV-Ctr;STZ), 0.0047 (I, Time spent in target quadrant, AAV-Ctr;STZ vs AAV-NCLX;STZ); <0.0001 (J, Target entries, AAV-Ctr:db/m vs AAV-Ctr:db/db), 0.0253 (J, Target entries, AAV-Ctr:db/db vs AAV-NCLX:db/db), <0.0001 (J, Time spent in target quadrant, AAV-Ctr:db/m vs AAV-Ctr:db/db), 0.0007 (J, Time spent in target quadrant, AAV-Ctr:db/db vs AAV-NCLX:db/db).

(K): MMP measurement using JC-1 dye in primary hippocampal neurons transfected with Dnajb4 overexpression plasmid or control plasmid (n = 3 replicates). Scale bar: 200 μm. *P* value: 0.0008.

(L): Reciprocal endogenous immunoprecipitation (IP) was performed to detect interactions between NCLX and Dnajb4 in primary hippocampal neurons (n = 3 replicates).

(M): Immunoblots and quantification analysis of protein level of NCLX in the hippocampal CA1 neurons of Ctr Bhlhe41$^{fl/fl}$ or STZ Bhlhe41$^{fl/fl}$ mice injected with AAV-CAMKII or AAV-CAMKII-Cre (n = 9 mice per group). *P* values: <0.0001 (Bhlhe41$^{f/f}$;Ctr vs Bhlhe41$^{f/f}$;STZ), <0.0001 (Bhlhe41$^{f/f}$;STZ vs Bhlhe41^{cKO};STZ).

Data are means ± SEM. *P < 0.05, **P < 0.01, ***P < 0.001. Two-tailed Student's unpaired t test analysis (A-C), Two-way ANOVA followed by Tukey's test (D-I and K).

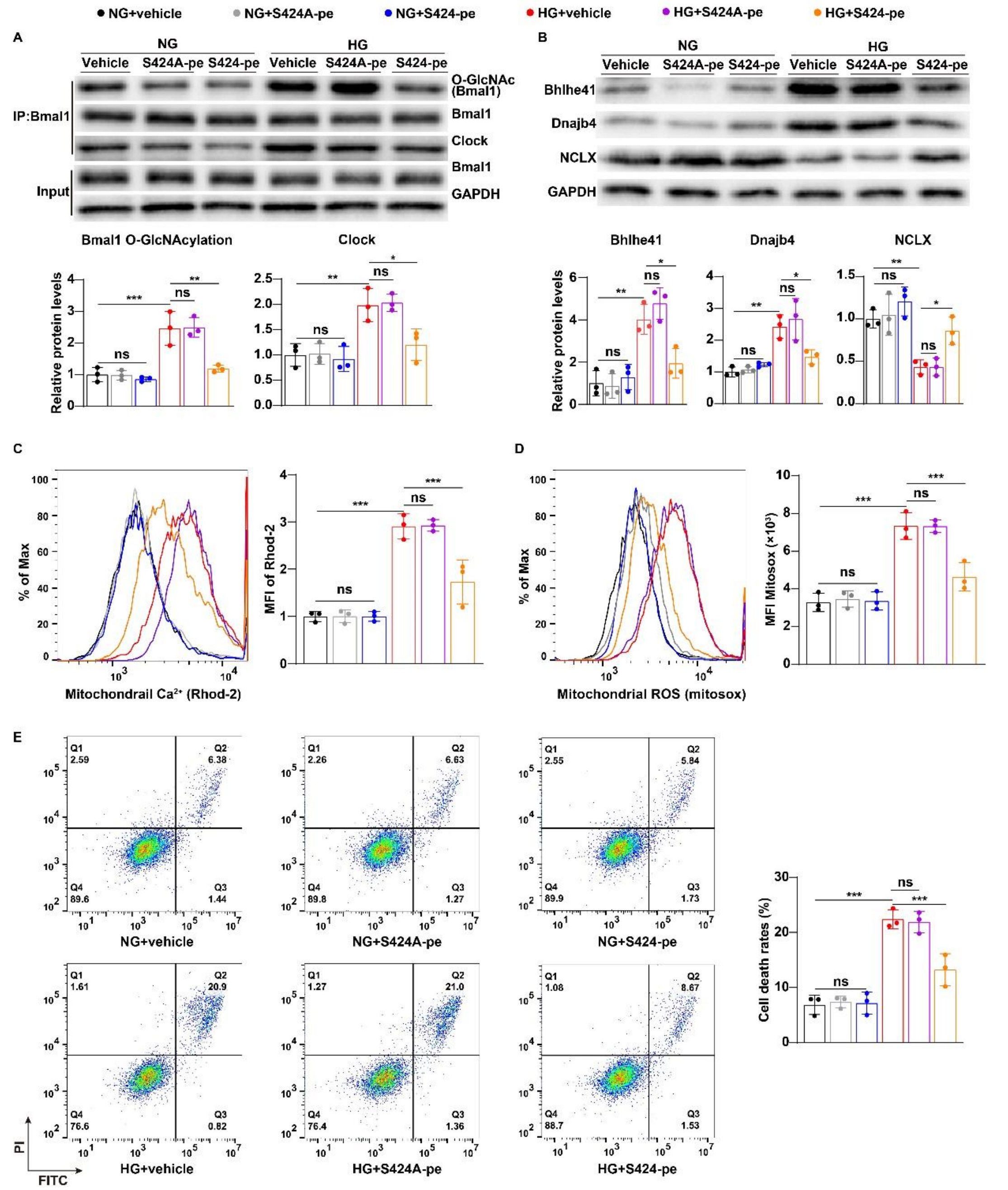

Appendix Figure S7. Short Peptide S424-pe Protects Against High Glucose-Induced Neuronal Apoptosis and Cognitive Dysfunction by Regulating Bhlhe41/Dnajb4/NCLX Signaling

(A): Primary hippocampal neurons were treated with normal glucose (NG, 5.5 mmol/L D-glucose) or high glucose (HG, 25 mmol/L D-glucose) in the presence of vehicle, S424A-pe (20 μM) or S424-pe (20 μM), and cell lysates were immunoprecipitated with Bmal1 antibodies and western blotted with the indicated antibodies (n = 3 replicates). *P* values: >0.9999 (Bmal1 O-GlcNAcylation, NG+Vehicle vs NG+S424A-pe), 0.9863

(Bmal1 O-GlcNAcylation, NG+Vehicle vs NG+S424-pe), 0.0004 (Bmal1 O-GlcNAcylation, NG+Vehicle vs HG+Vehicle), >0.9999 (Bmal1 O-GlcNAcylation, HG+Vehicle vs HG+ S424A-pe), 0.0014 (Bmal1 O-GlcNAcylation, HG+Vehicle vs HG+ S424-pe); >0.9999 (Clock, NG+Vehicle vs NG+S424A-pe), 0.9986 (Clock, NG+Vehicle vs NG+S424-pe), 0.005 (Clock, NG+Vehicle vs HG+Vehicle), 0.9998 (Clock, HG+Vehicle vs HG+ S424A-pe), 0.0254 (Clock, HG+Vehicle vs HG+ S424-pe).

(B): Immunoblots and quantification analysis of Bhlhe41, Dnajb4 and NCLX expression levels in primary hippocampal neurons treated with normal glucose (NG, 5.5 mmol/L D-glucose) or high glucose (HG, 25 mmol/L D-glucose) in the presence of vehicle, S424A-pe (20 μM) or S424-pe (20 μM) (n = 3 replicates).

P values: 0.9999 (Bhlhe41, NG+Vehicle vs NG+S424A-pe), 0.9938 (Bhlhe41, NG+Vehicle vs NG+S424-pe), 0.0012 (Bhlhe41, NG+Vehicle vs HG+Vehicle), 0.7163 (Bhlhe41, HG+Vehicle vs HG+ S424A-pe), 0.0212 (Bhlhe41, HG+Vehicle vs HG+ S424-pe); 0.9998 (Dnajb4, NG+Vehicle vs NG+S424A-pe), 0.9498 (Dnajb4, NG+Vehicle vs NG+S424-pe), 0.0019 (Dnajb4, NG+Vehicle vs HG+Vehicle), 0.9437 (Dnajb4, HG+Vehicle vs HG+ S424A-pe), 0.0349 (Dnajb4, HG+Vehicle vs HG+ S424-pe); 0.9987 (NCLX, NG+Vehicle vs NG+S424A-pe), 0.5834 (NCLX, NG+Vehicle vs NG+S424-pe), 0.0075 (NCLX, NG+Vehicle vs HG+Vehicle), >0.9999 (NCLX, HG+Vehicle vs HG+ S424A-pe), 0.0485 (NCLX, HG+Vehicle vs HG+ S424-pe).

(C-E): Flow cytometry analysis of mitochondrial calcium levels (C), mtROS levels (D) and apoptosis levels (E) in primary hippocampal neurons with different treatments as indicated (n = 3 replicates). *P* values: >0.9999 (C, NG+Vehicle vs NG+S424A-pe), >0.9999 (C, NG+Vehicle vs NG+S424-pe), <0.0001 (C, NG+Vehicle vs HG+Vehicle), >0.9999 (C, HG+Vehicle vs HG+ S424A-pe), 0.0006 (C, HG+Vehicle vs HG+ S424-pe); 0.9981 (D, NG+Vehicle vs NG+S424A-pe), >0.9999 (D, NG+Vehicle vs NG+S424-pe), <0.0001 (D, NG+Vehicle vs HG+Vehicle), >0.9999 (D, HG+Vehicle vs HG+ S424A-pe), 0.0007 (D, HG+Vehicle vs HG+ S424-pe); 0.9994 (E, NG+Vehicle vs NG+S424A-pe), >0.9999 (E, NG+Vehicle vs NG+S424-pe), <0.0001 (E, NG+Vehicle vs HG+Vehicle), 0.9995 (E, HG+Vehicle vs HG+ S424A-pe), 0.001

(E, HG+Vehicle vs HG+ S424-pe).

Data are means ± SEM. ns, not significant, *$P < 0.05$, **$P < 0.01$, ***$P < 0.001$. Two-way ANOVA followed by Tukey's test (A-E).
