# Supplementary figures and images for: O-GlcNAcylation of circadian clock protein Bmal1 impairs cognitive function in diabetic mice
Source: EMBO J. 2024 Oct 7;43(22):5667–89. doi: 10.1038/s44318-024-00263-6 (PMC11574178; doi:10.1038/s44318-024-00263-6)

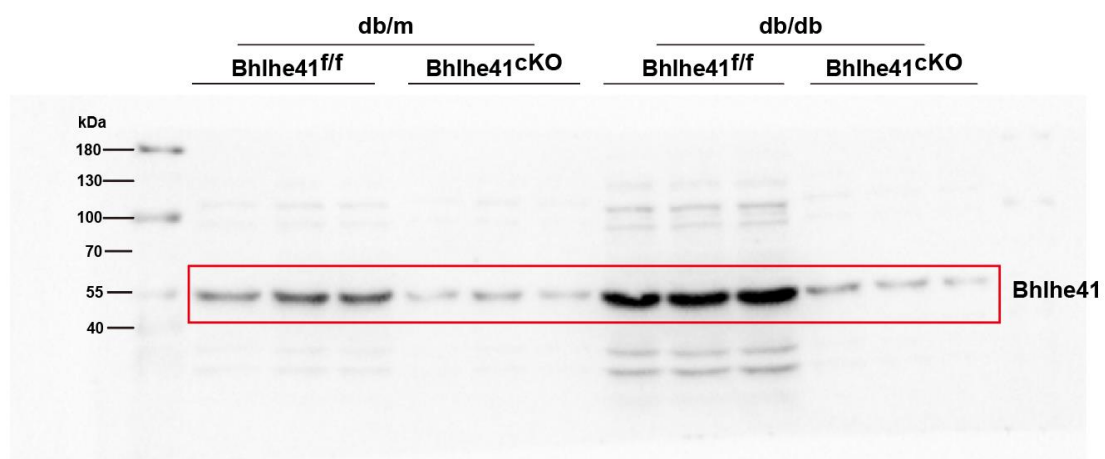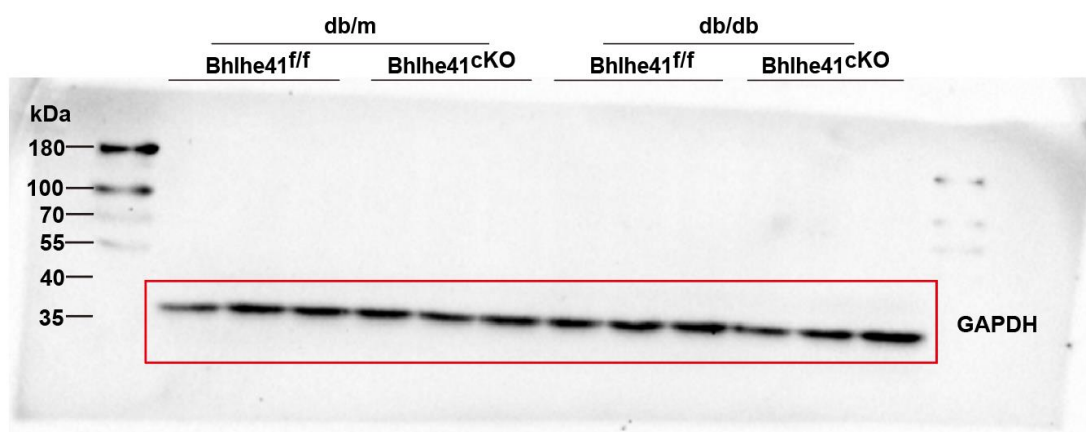

Supplement: Supplementary file 3 — Source data Fig. 1 [file 44318_2024_263_MOESM3_ESM.zip › Figure 1/1D/1D Western blot description for cropped image.pdf]

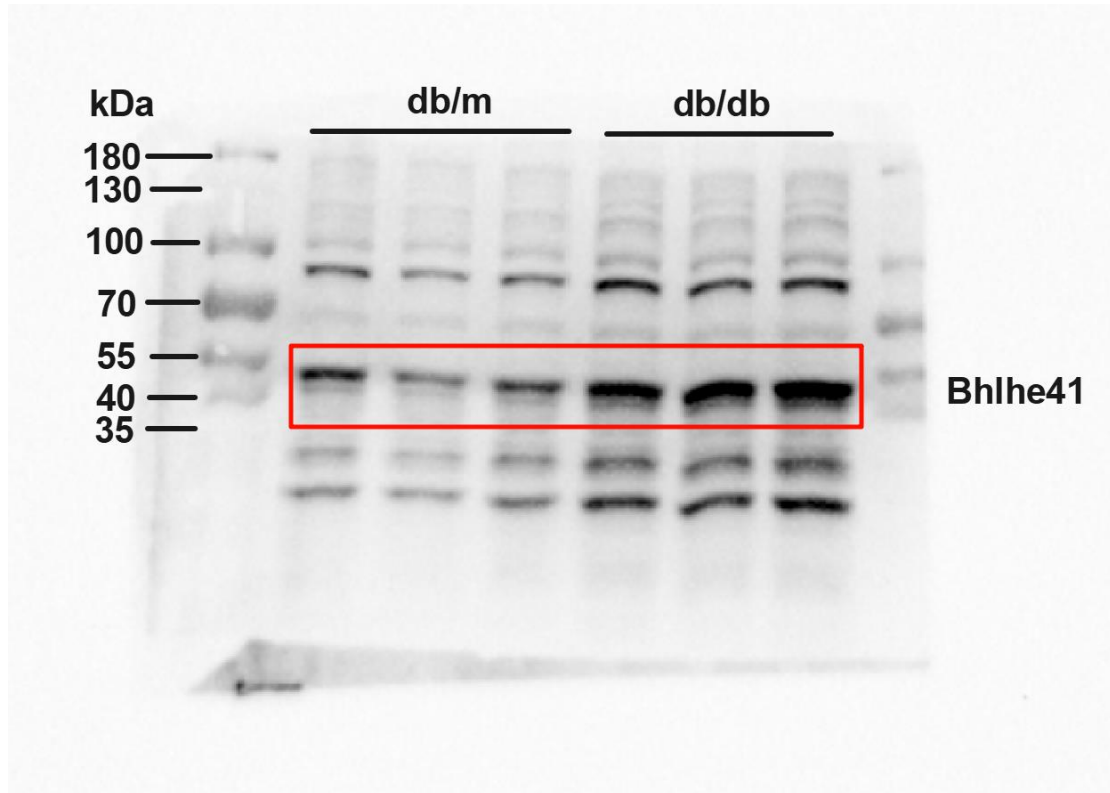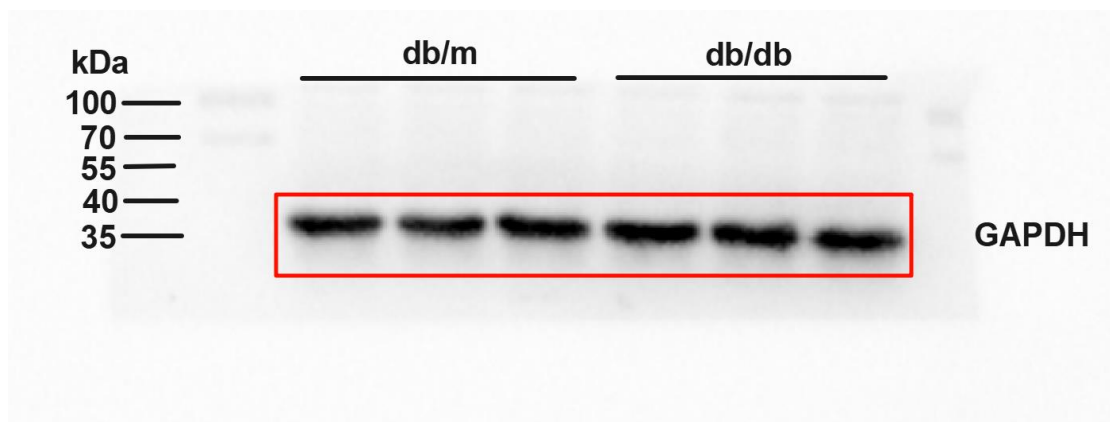

Supplement: Supplementary file 3 — Source data Fig. 1 [file 44318_2024_263_MOESM3_ESM.zip › Figure 1/1C/1C Western blot description for cropped image.pdf]

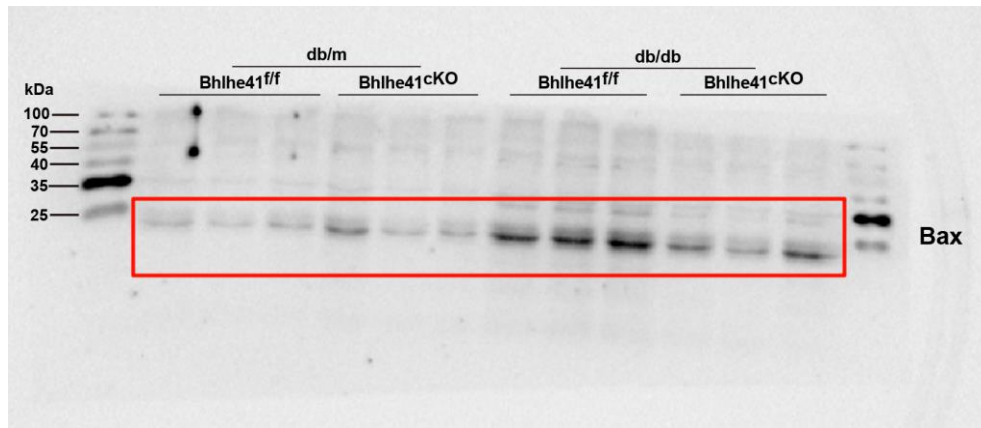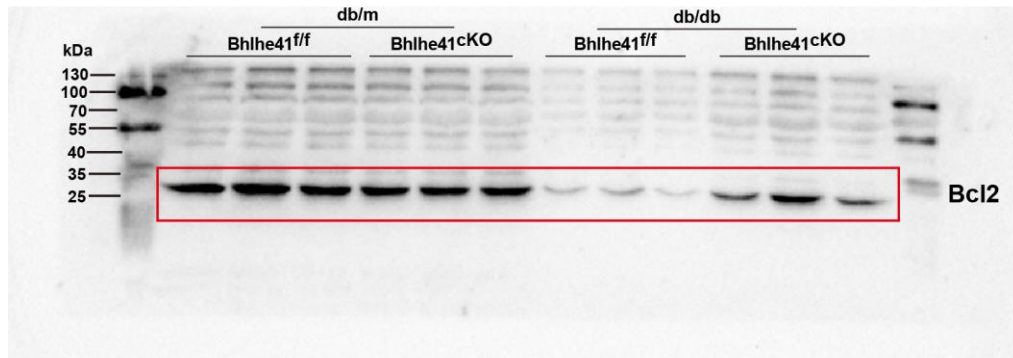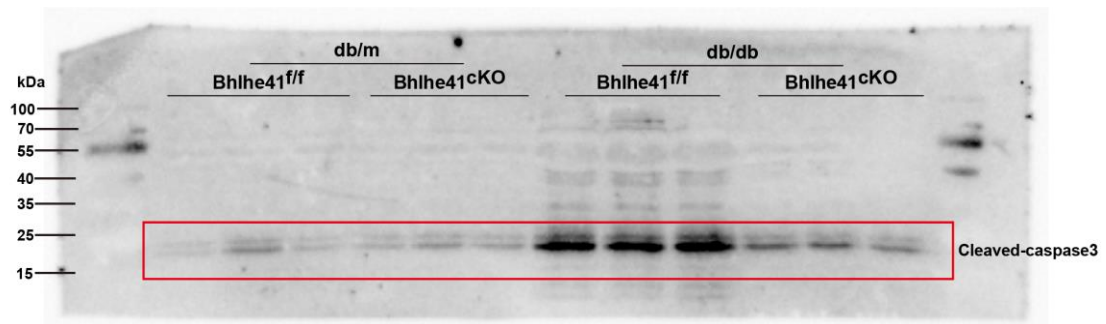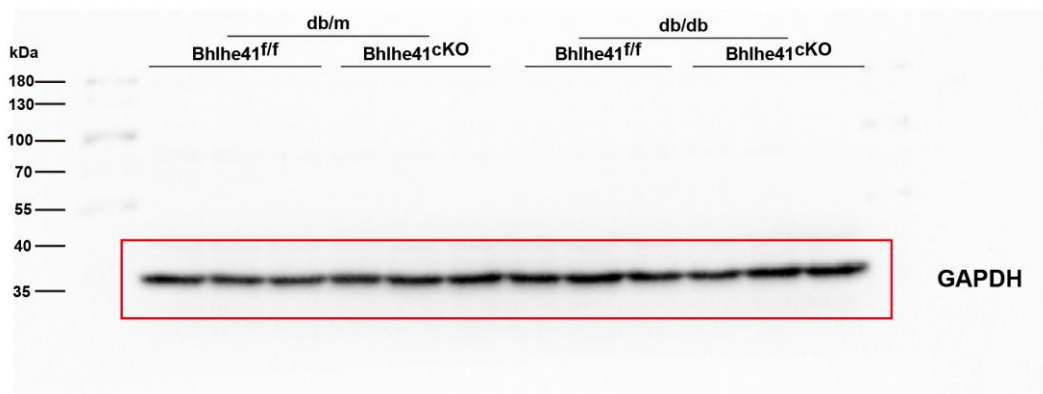

Supplement: Supplementary file 4 — Source data Fig. 2 [file 44318_2024_263_MOESM4_ESM.zip › Figure 2/2D/2D Western blot description for cropped image.pdf]

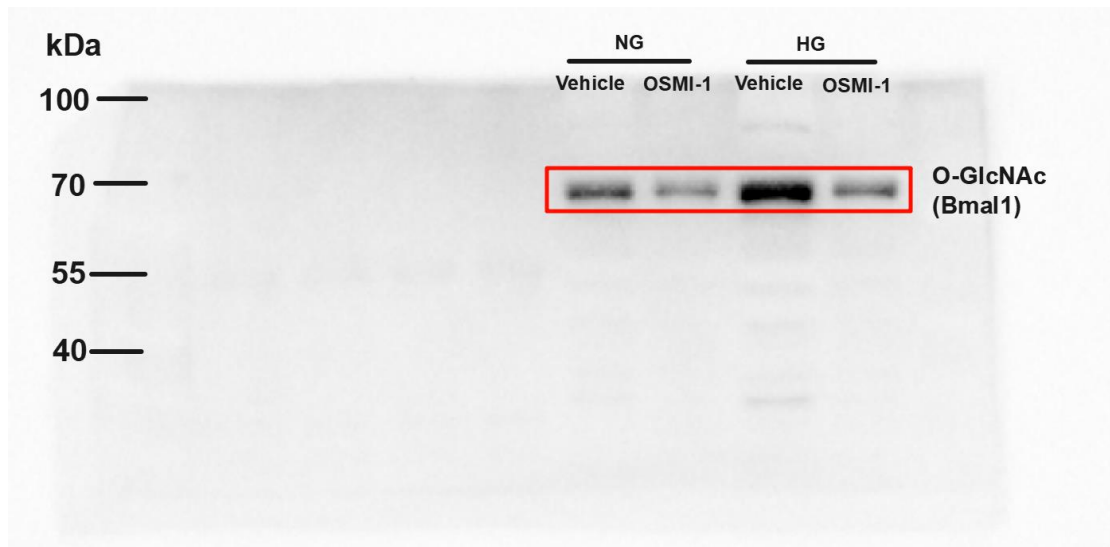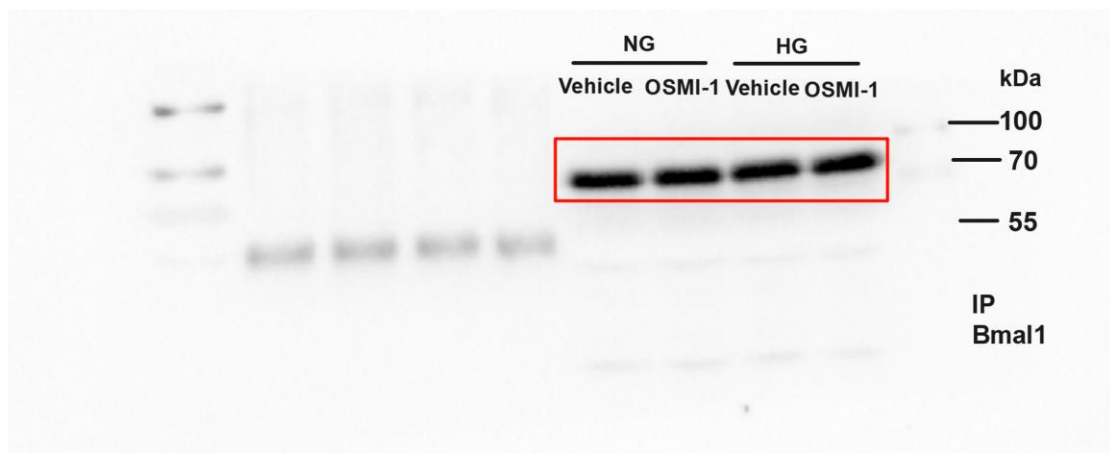

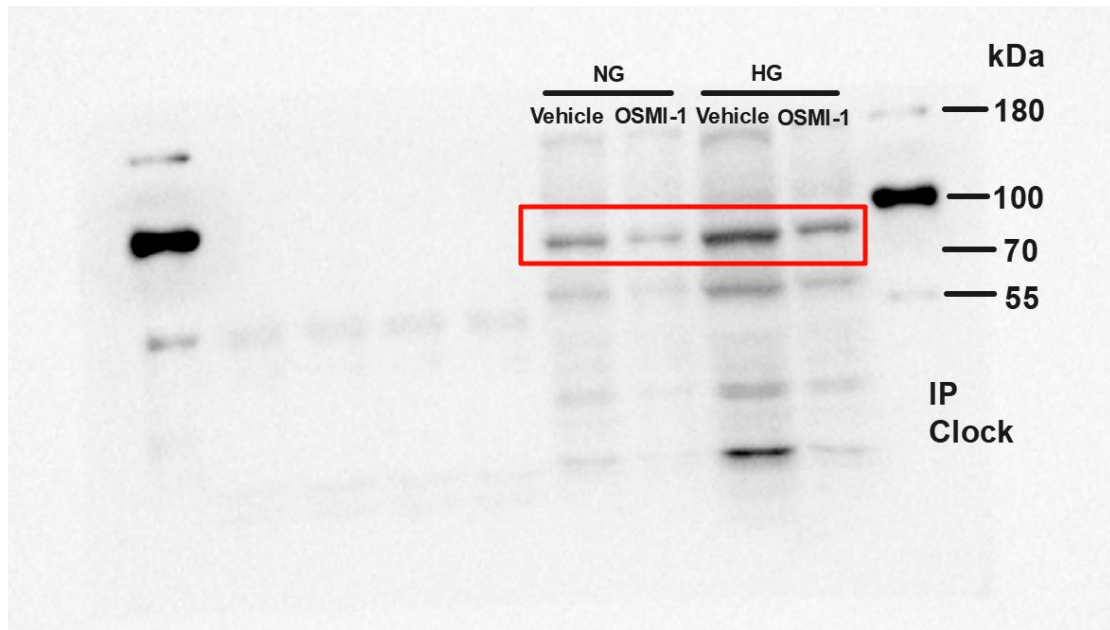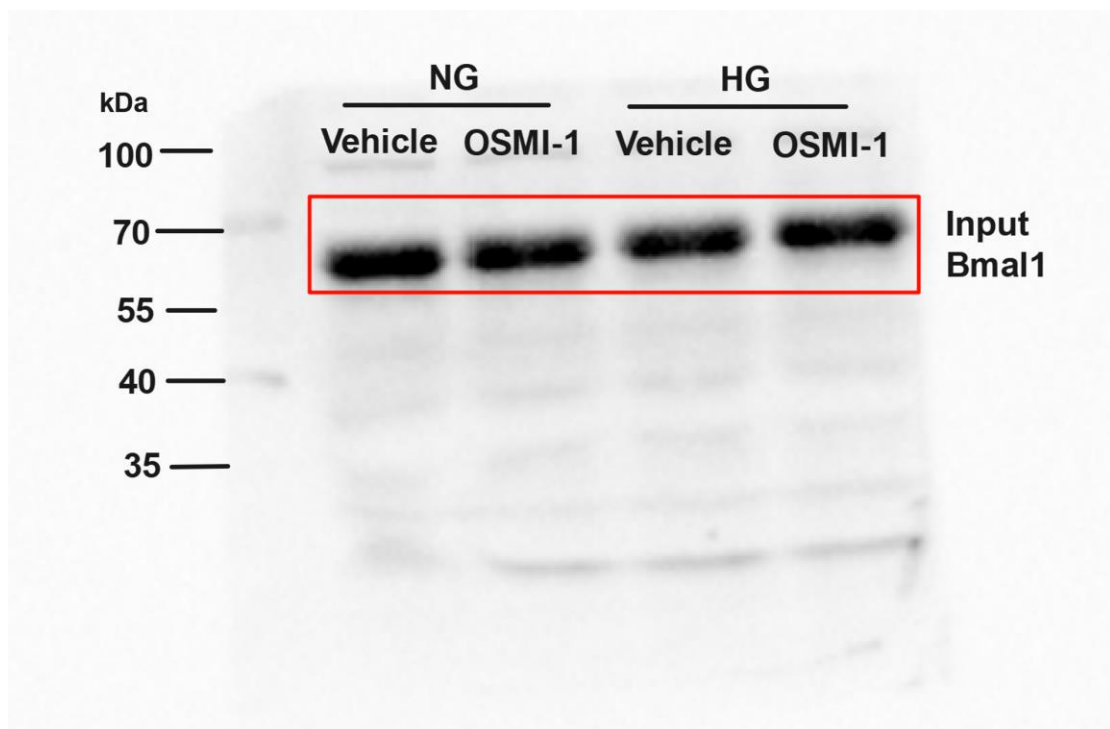

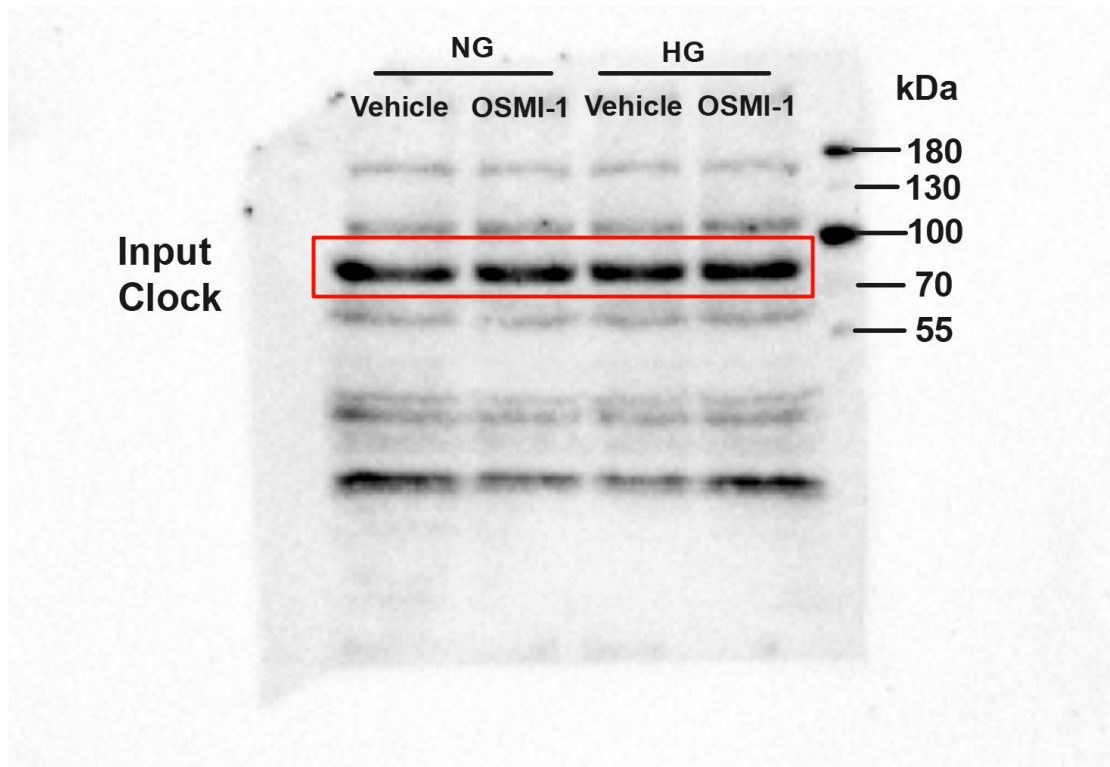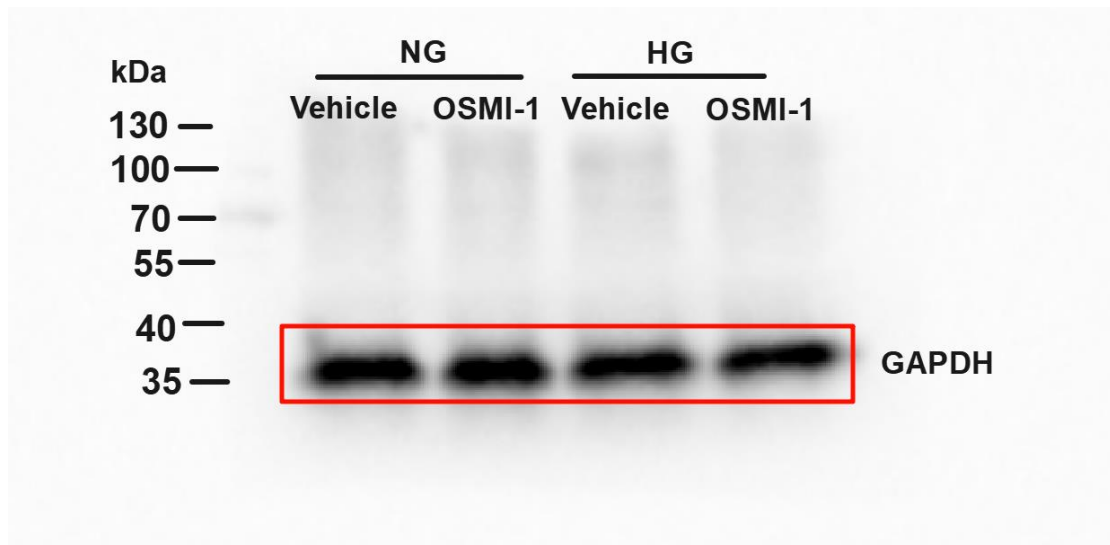

Supplement: Supplementary file 5 — Source data Fig. 3 [file 44318_2024_263_MOESM5_ESM.zip › Figure 3/3E/3E Western blot description for cropped image.pdf]

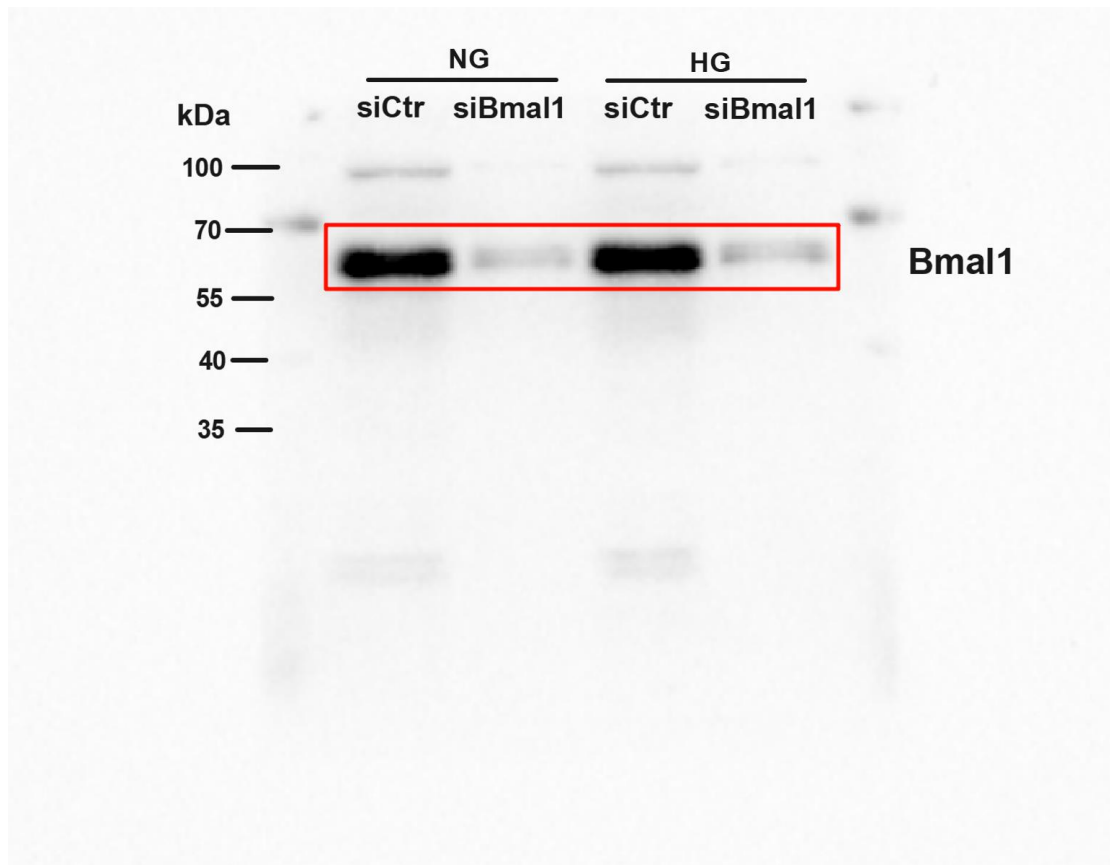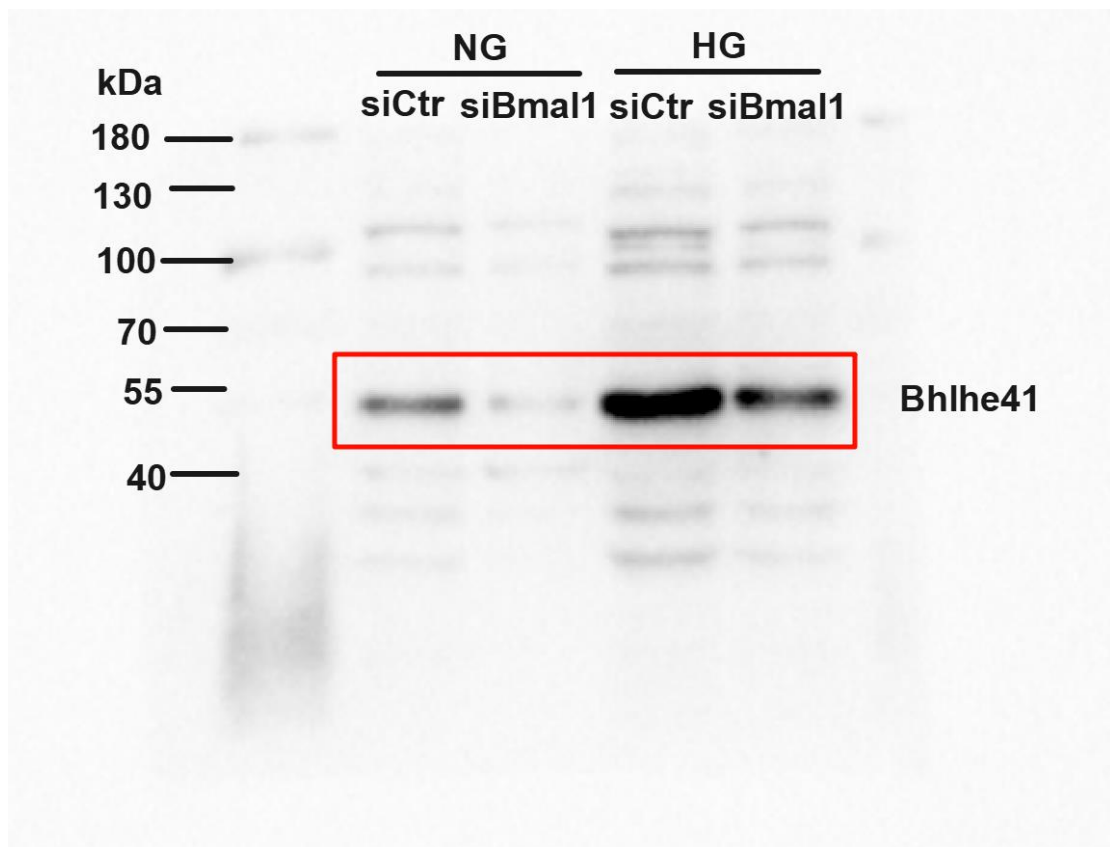

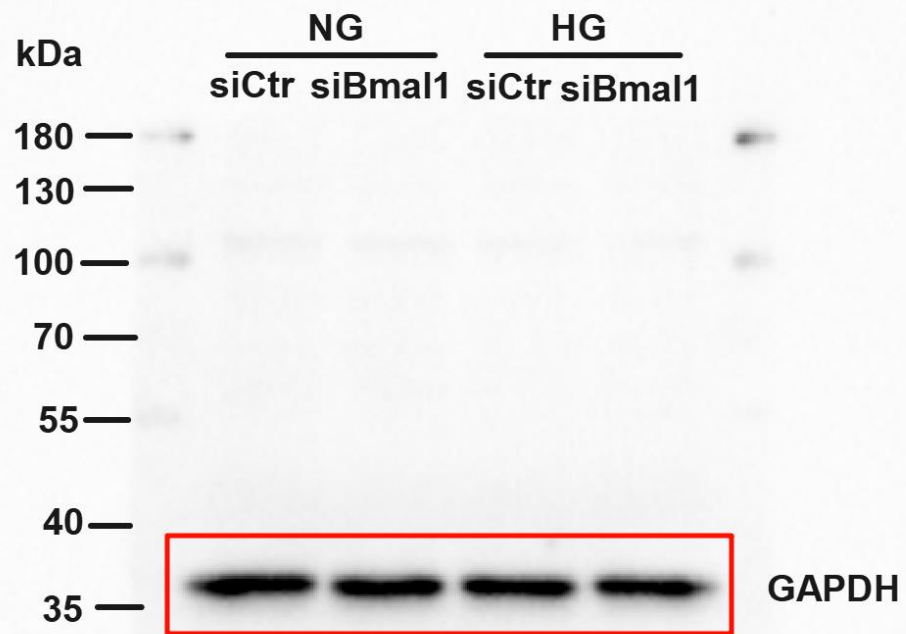

Supplement: Supplementary file 5 — Source data Fig. 3 [file 44318_2024_263_MOESM5_ESM.zip › Figure 3/3B/3B Western blot description for cropped image.pdf]

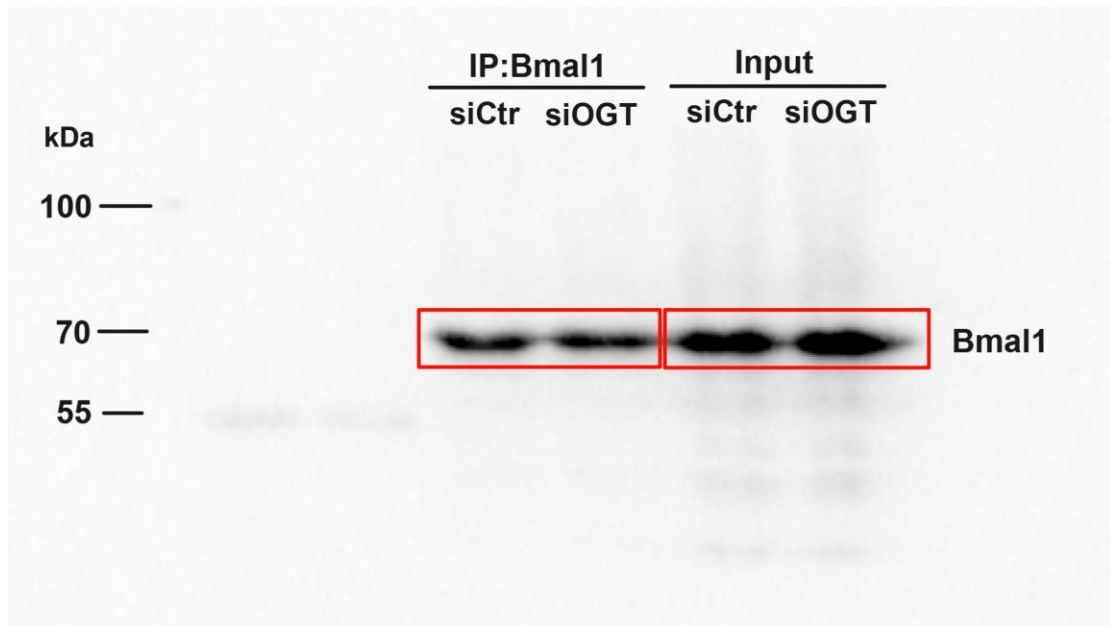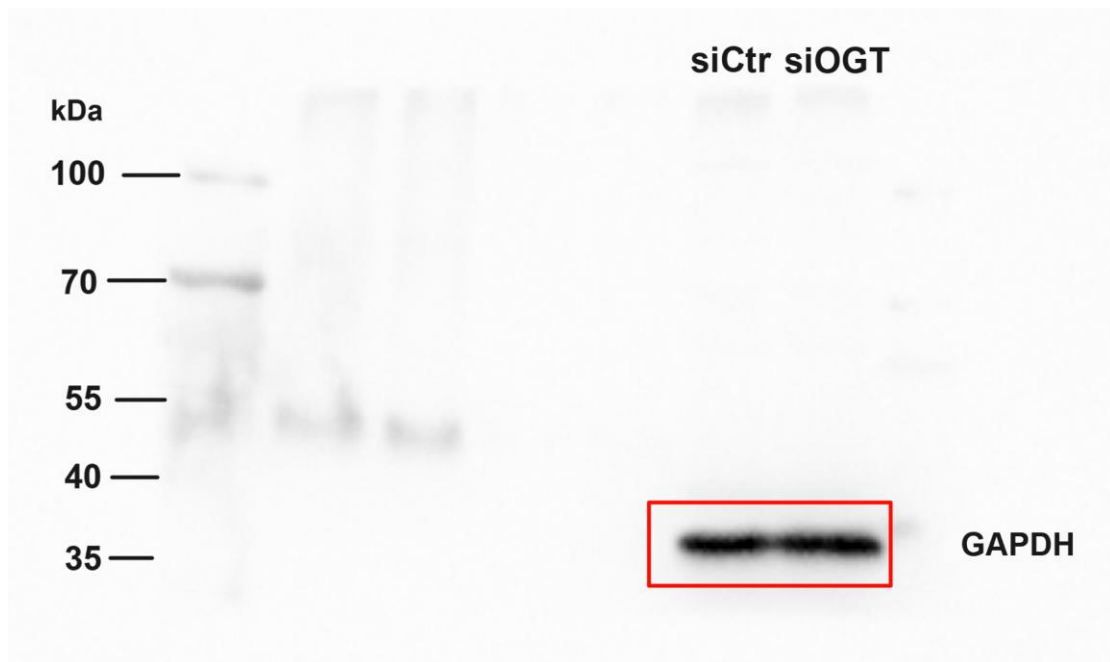

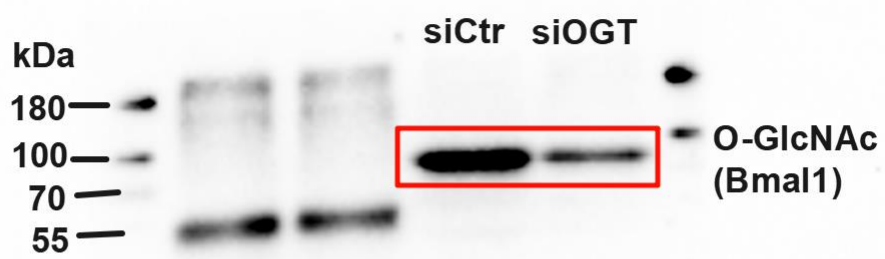

Supplement: Supplementary file 5 — Source data Fig. 3 [file 44318_2024_263_MOESM5_ESM.zip › Figure 3/3C/3C Western blot description for cropped image.pdf]

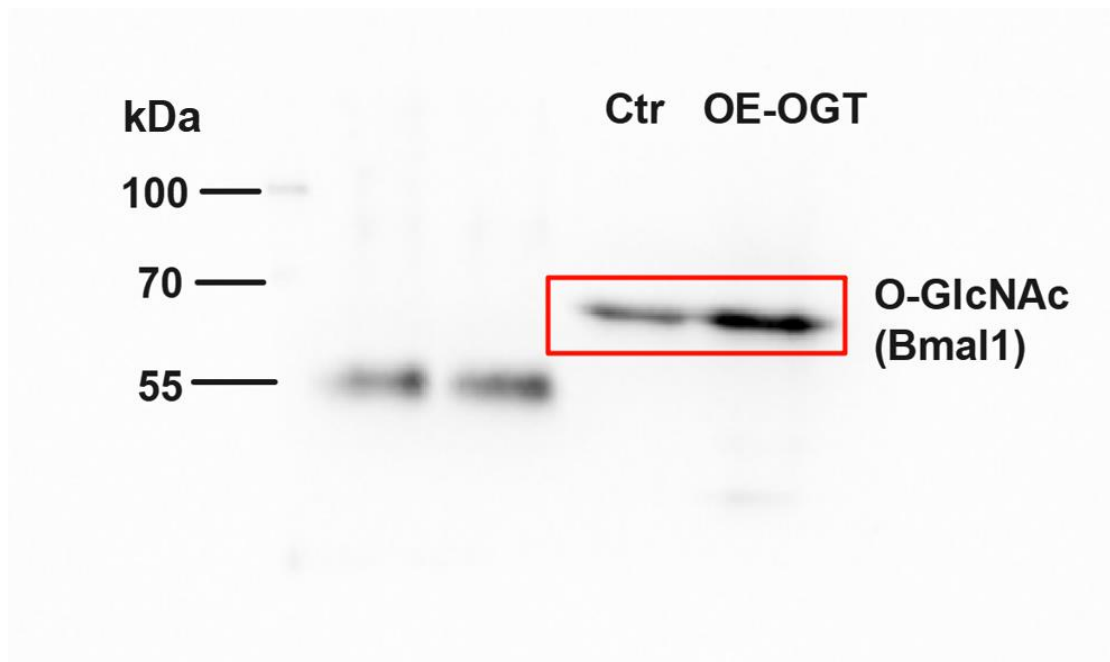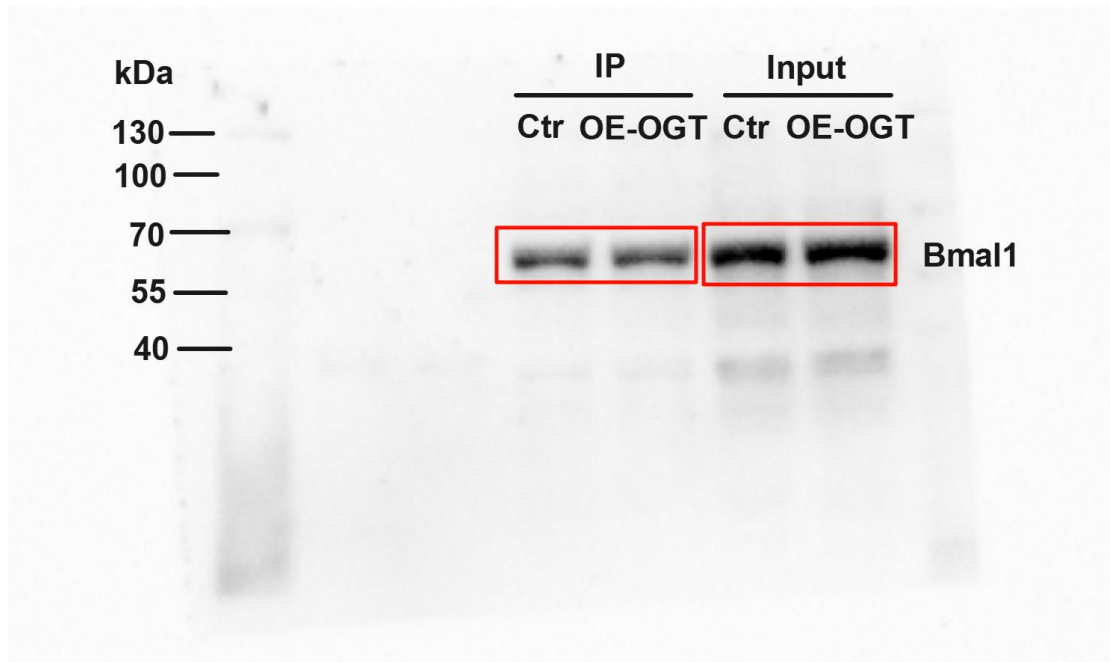

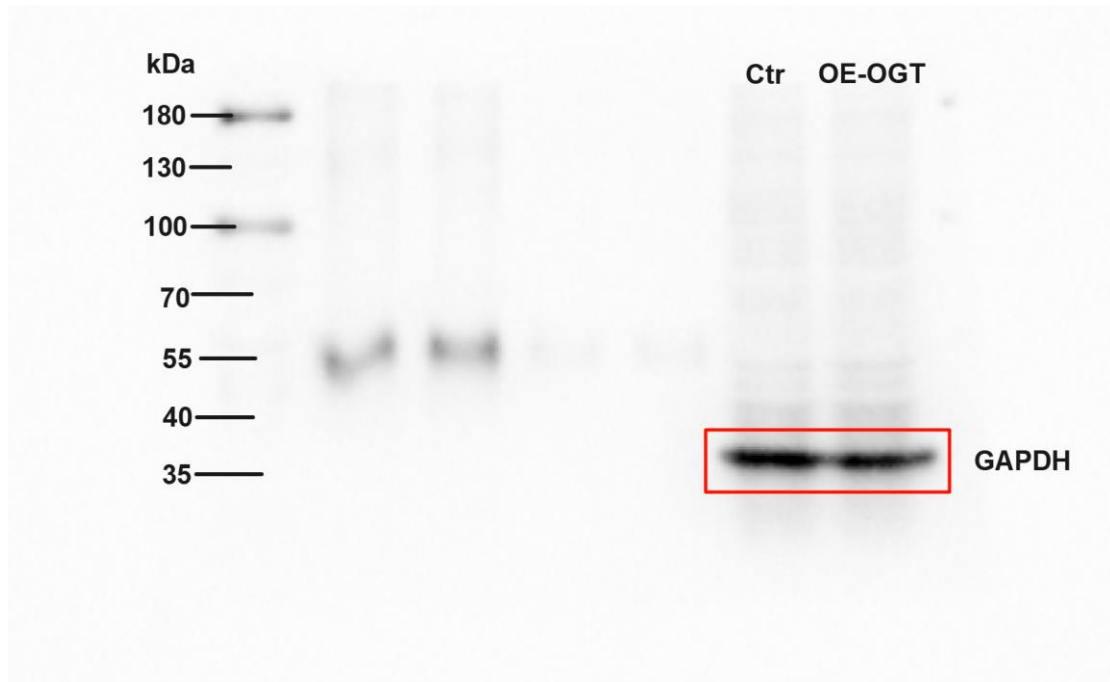

Supplement: Supplementary file 5 — Source data Fig. 3 [file 44318_2024_263_MOESM5_ESM.zip › Figure 3/3D/3D Western blot description for cropped image.pdf]

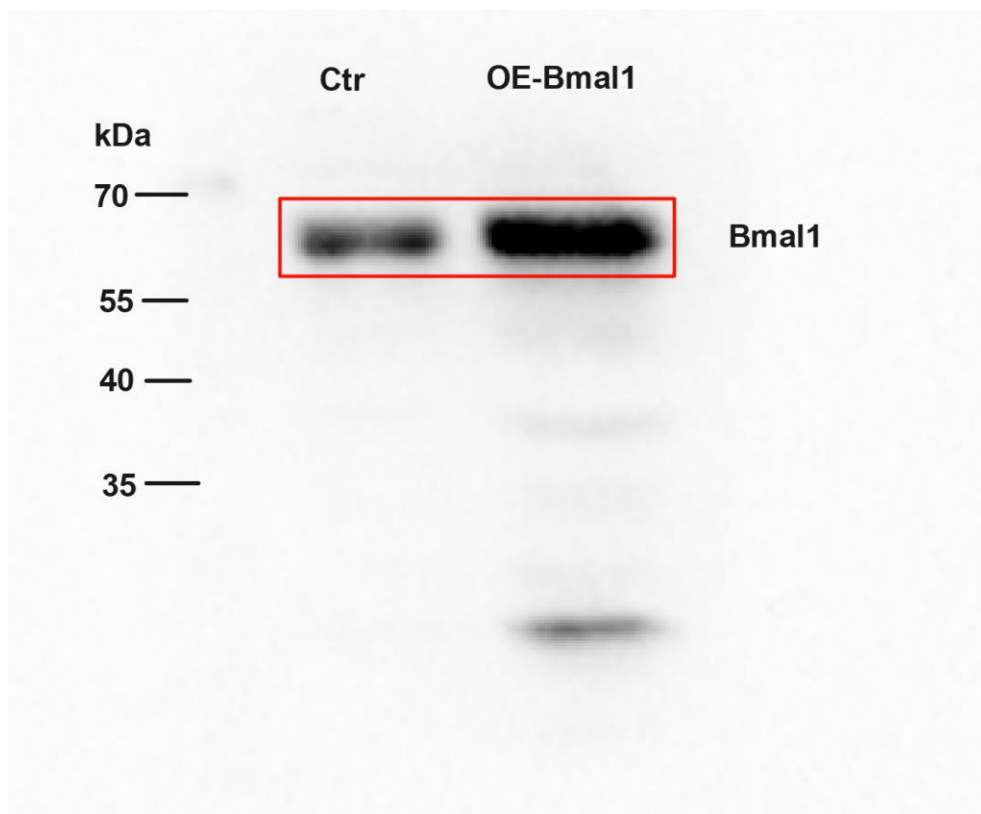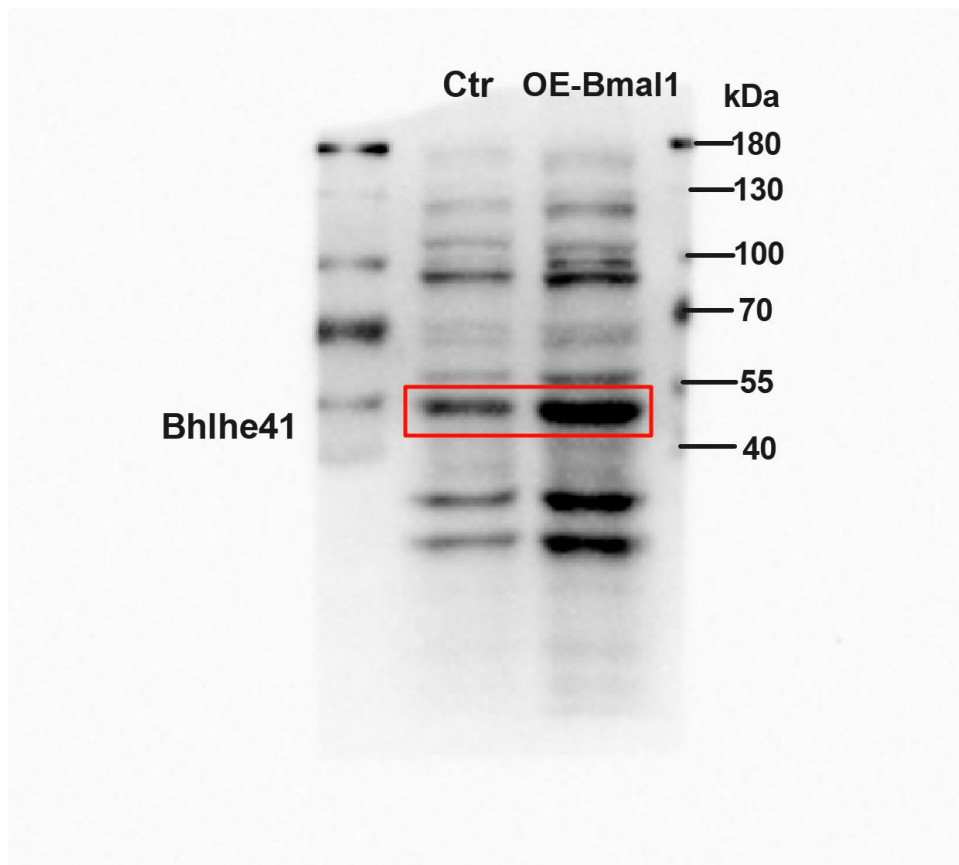

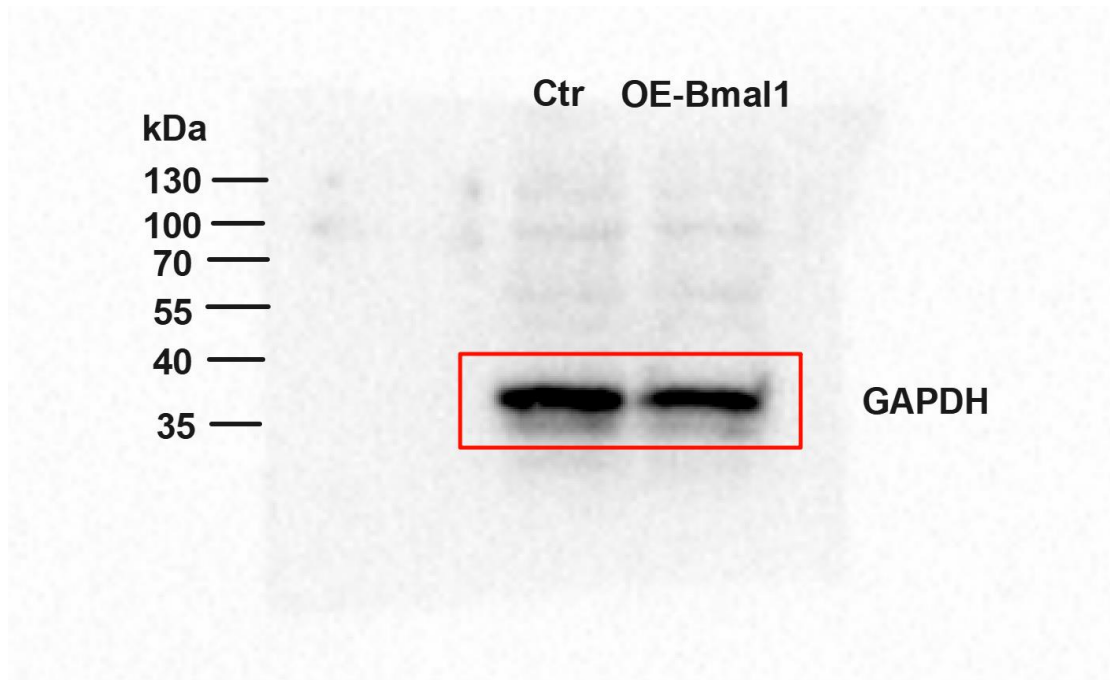

Supplement: Supplementary file 5 — Source data Fig. 3 [file 44318_2024_263_MOESM5_ESM.zip › Figure 3/3A/3A Western blot description for cropped image.pdf]

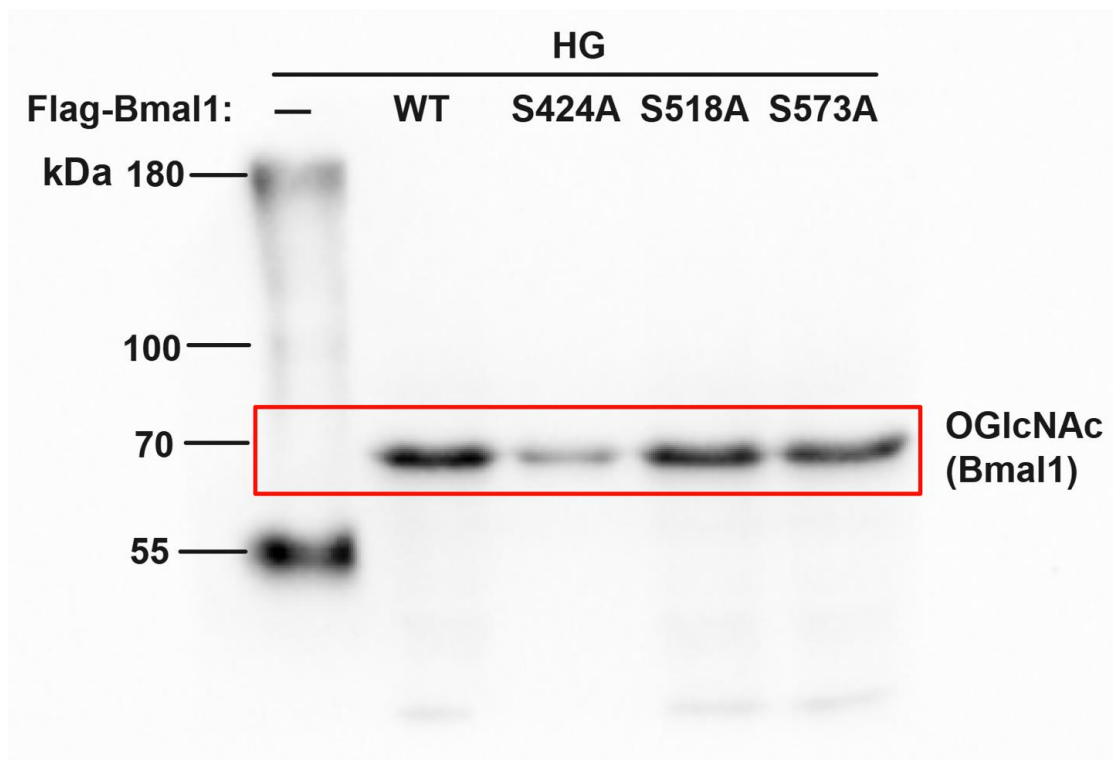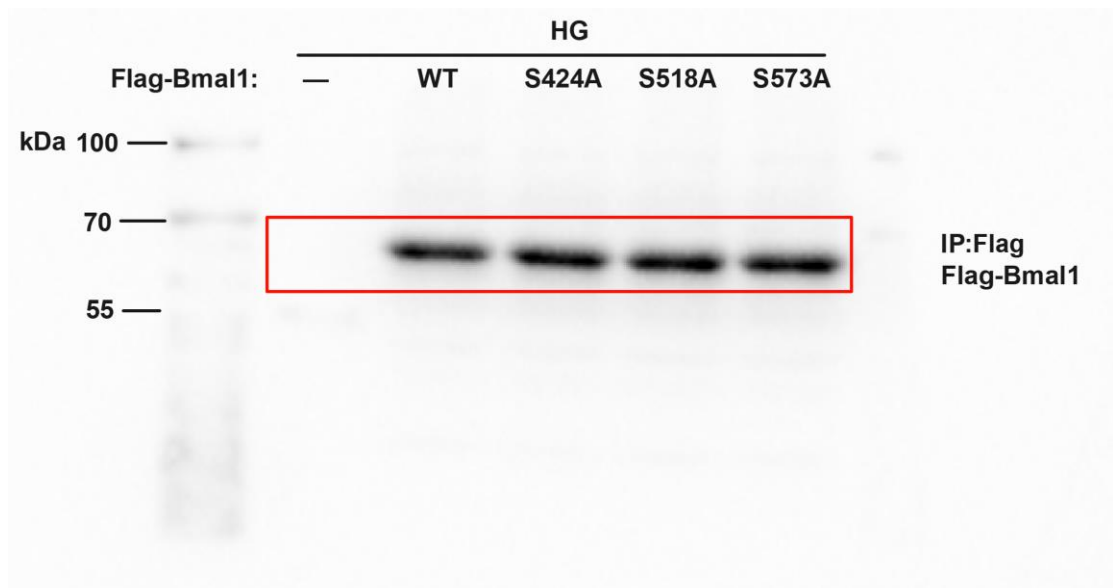

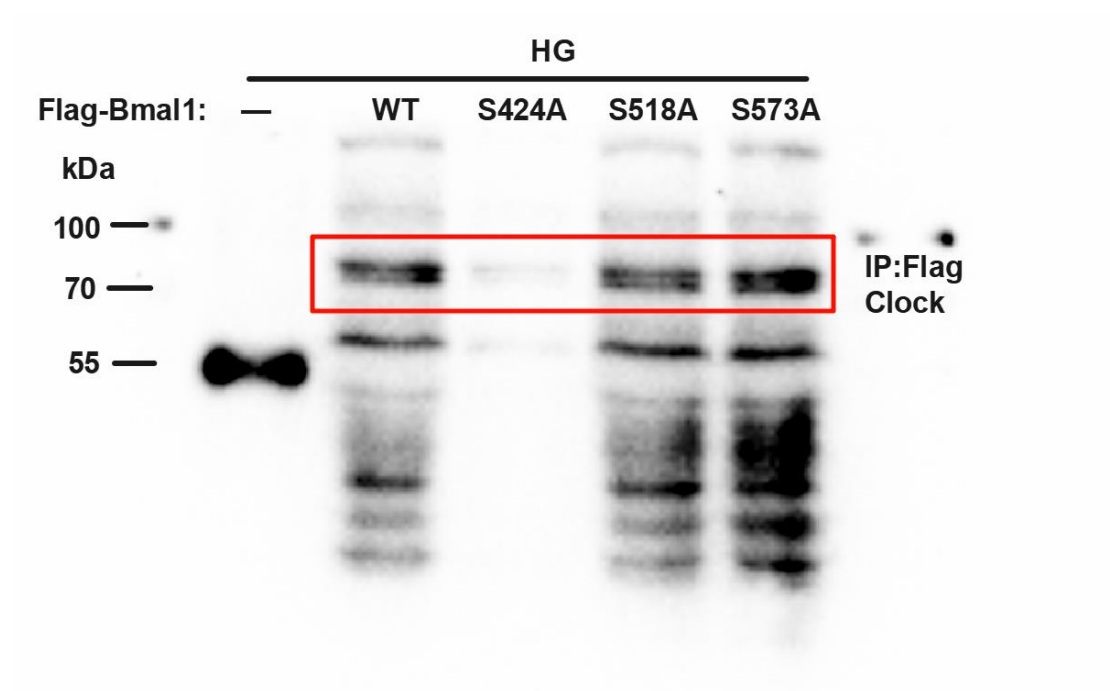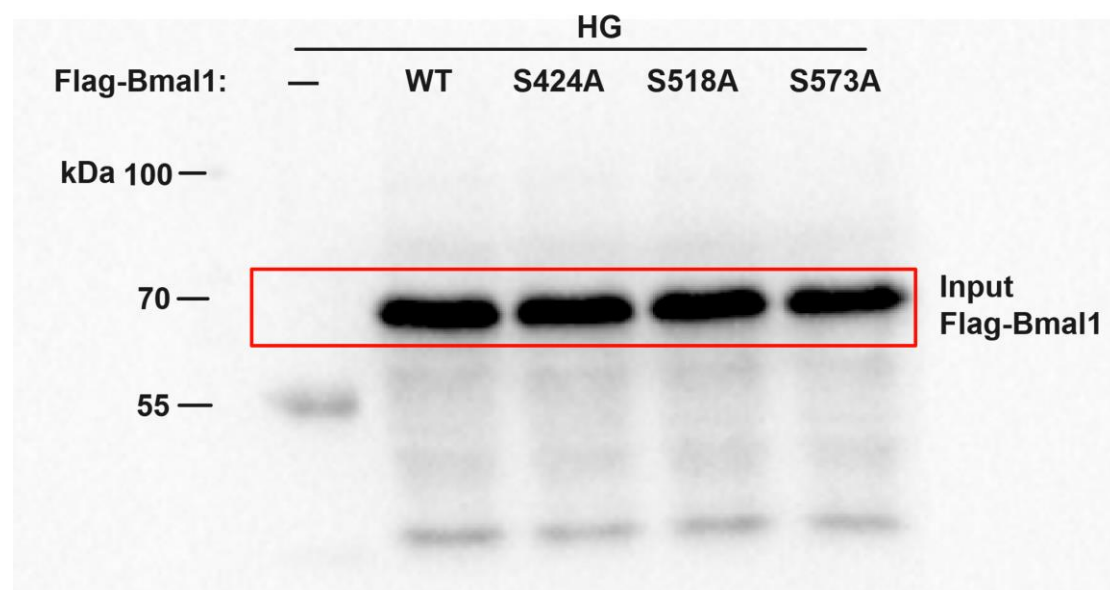

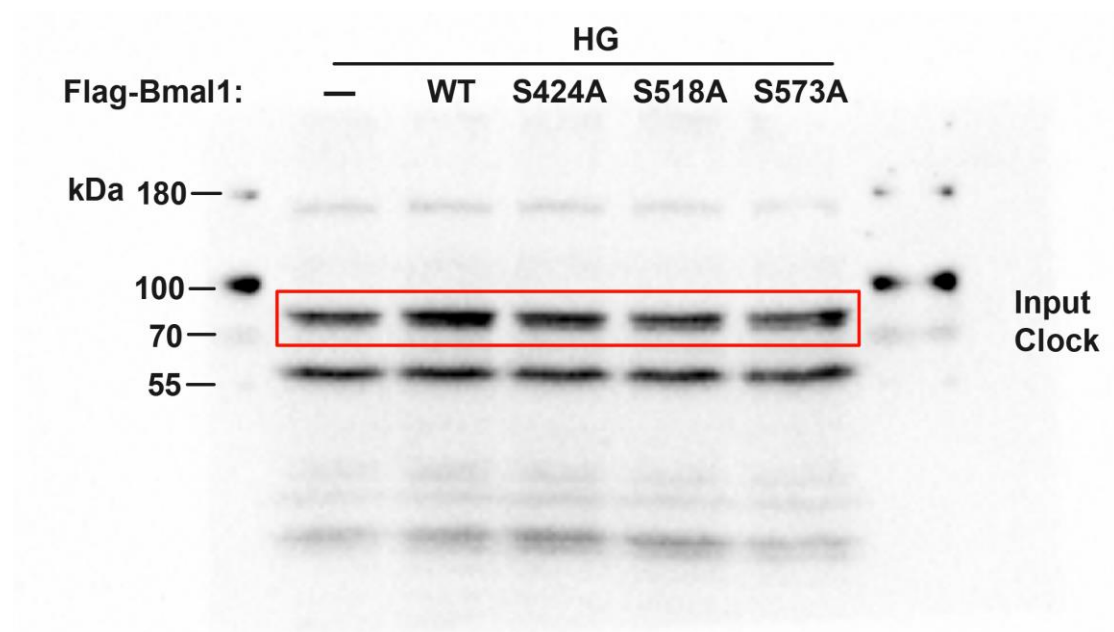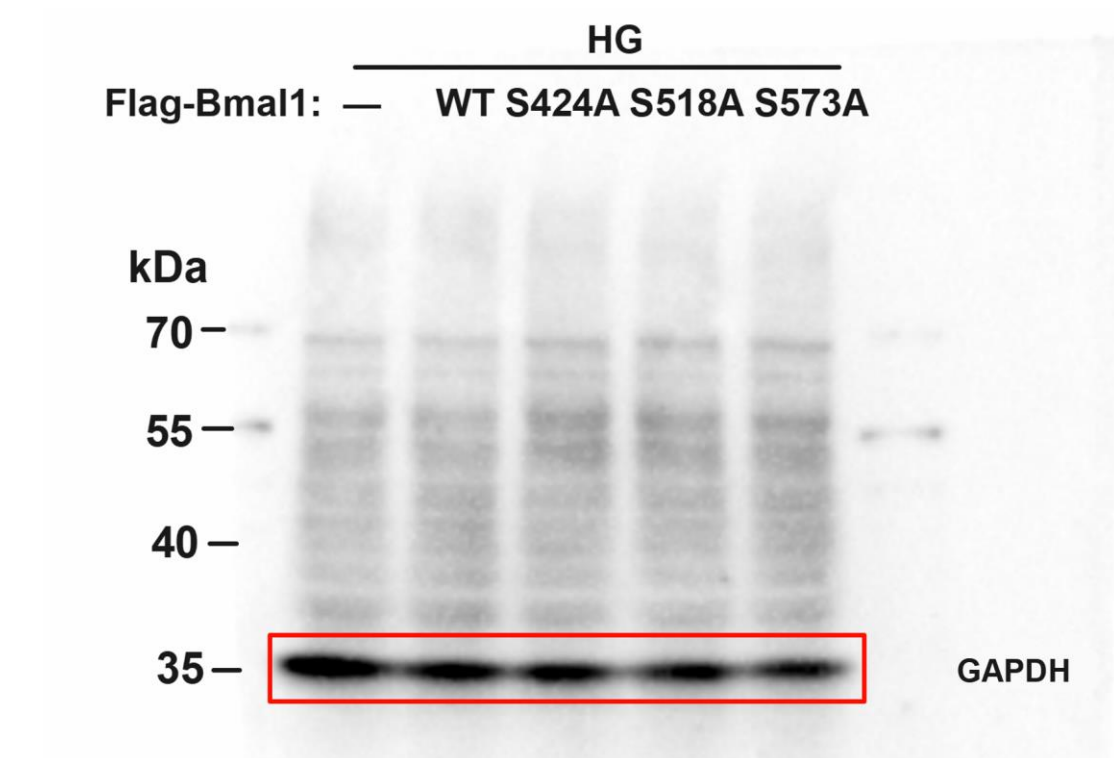

Supplement: Supplementary file 5 — Source data Fig. 3 [file 44318_2024_263_MOESM5_ESM.zip › Figure 3/3F/3F Western blot description for cropped image.pdf]

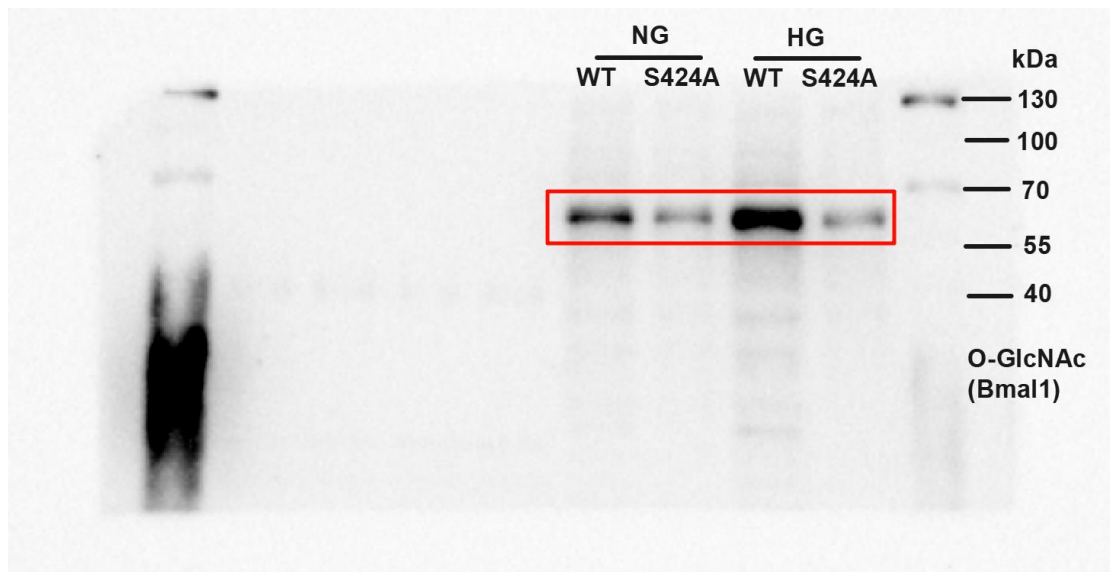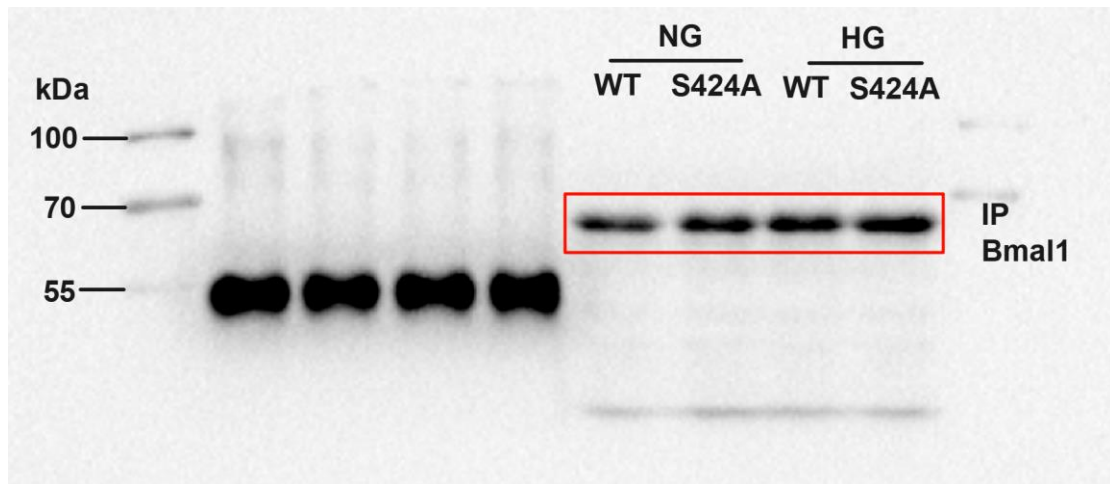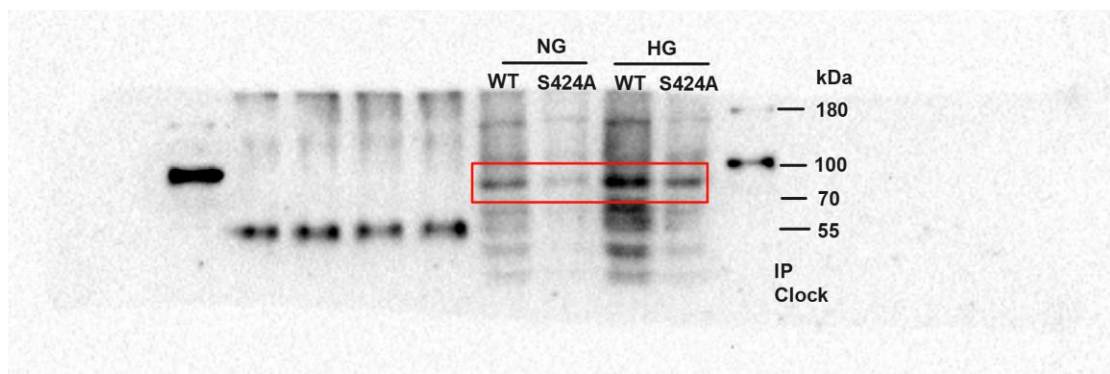

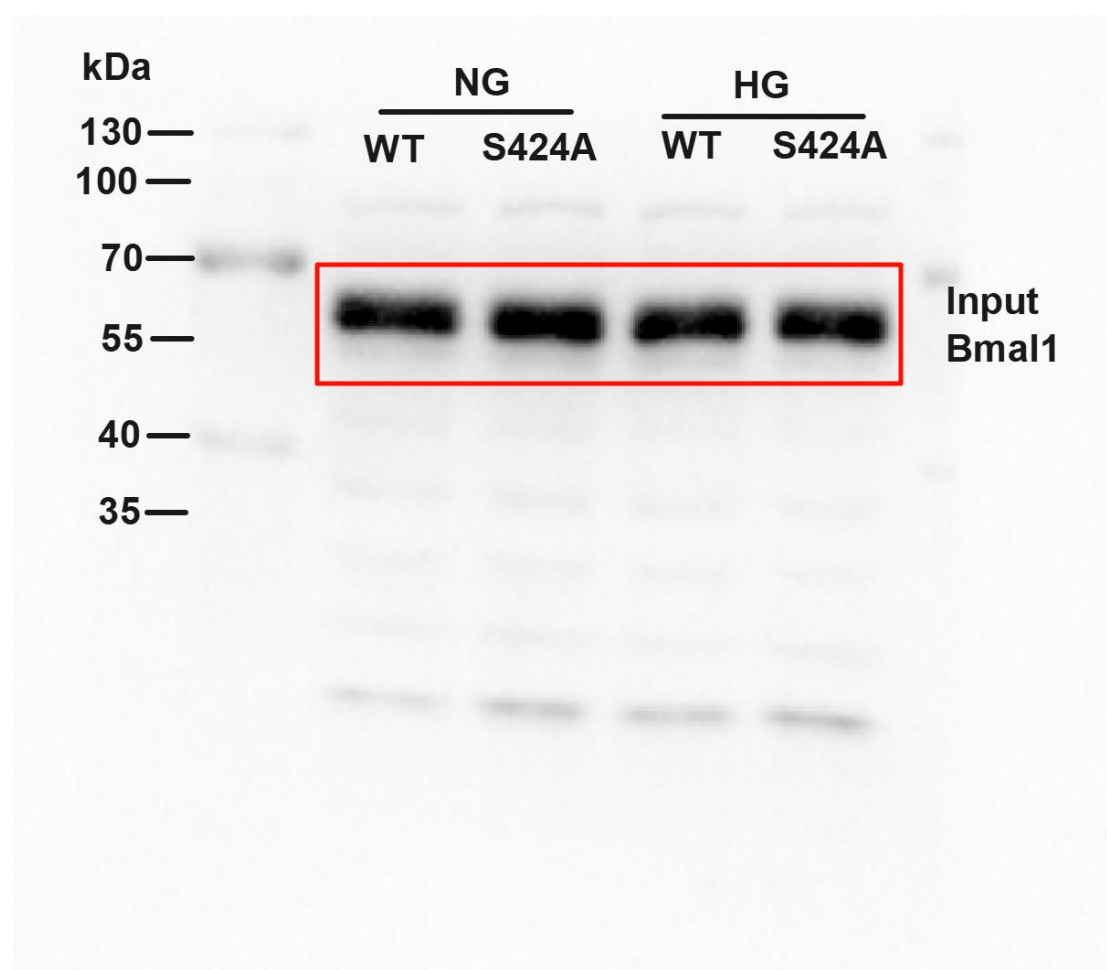

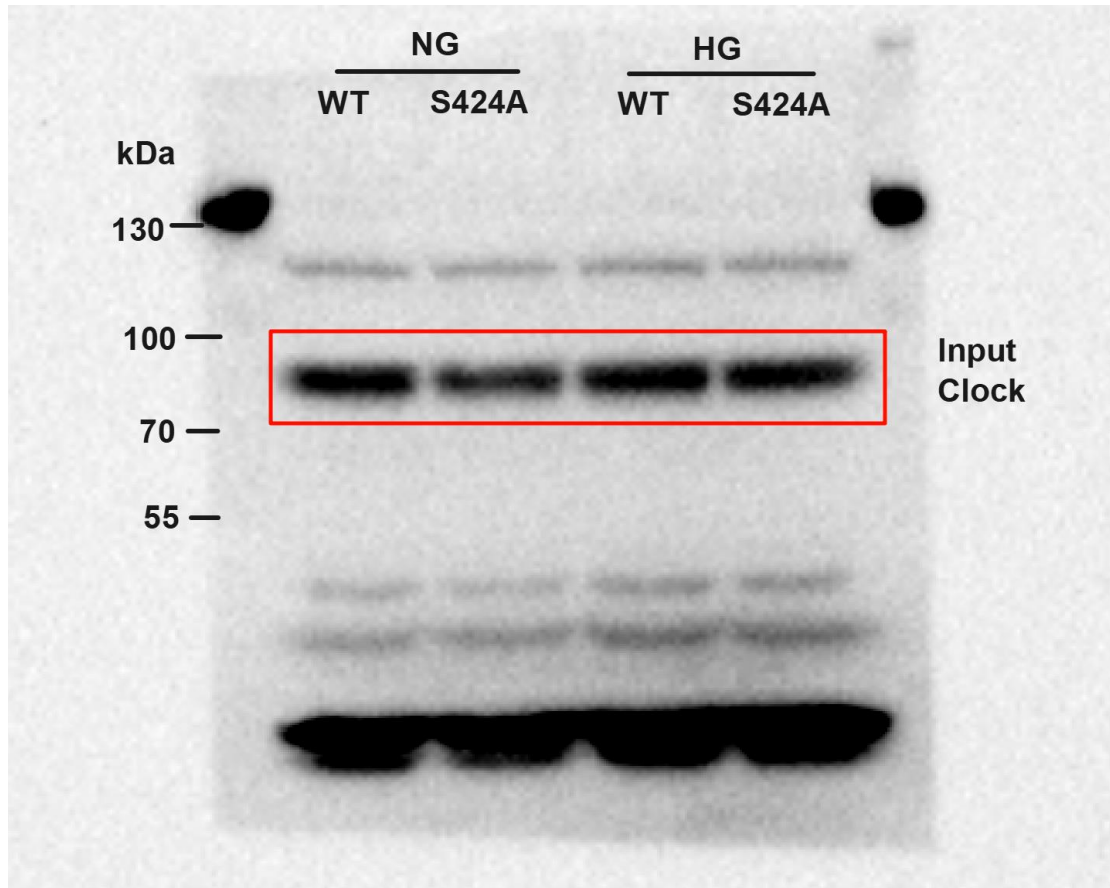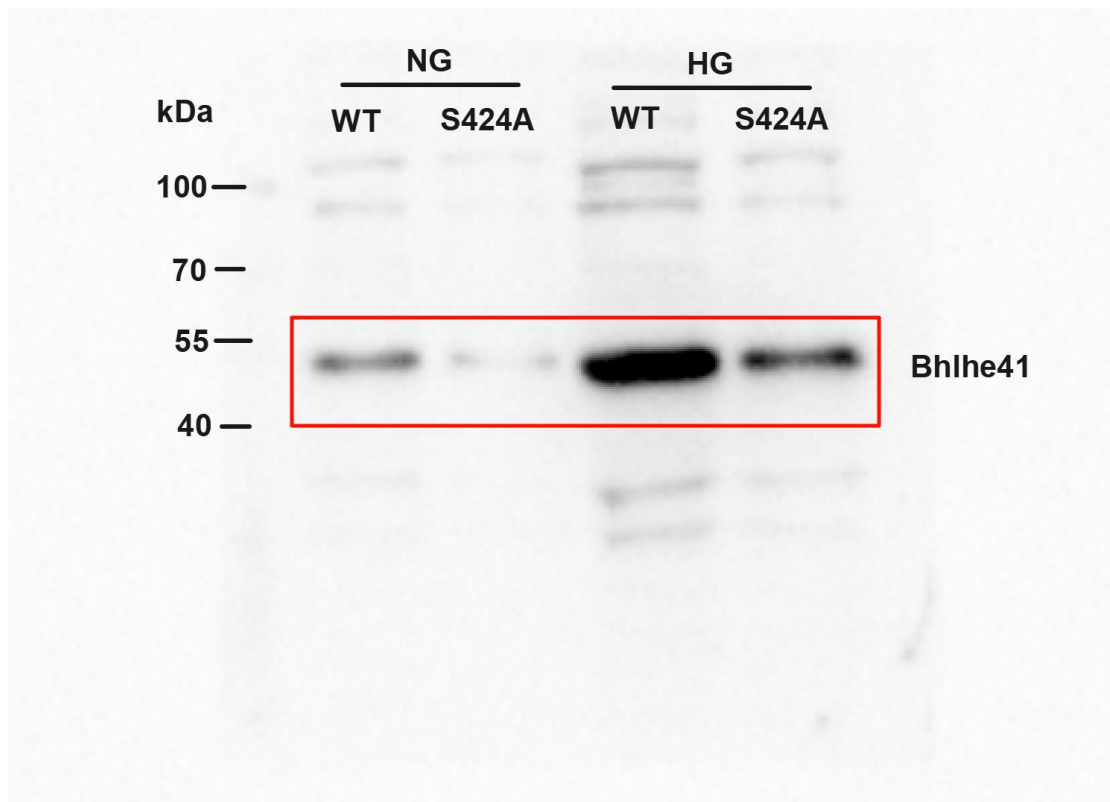

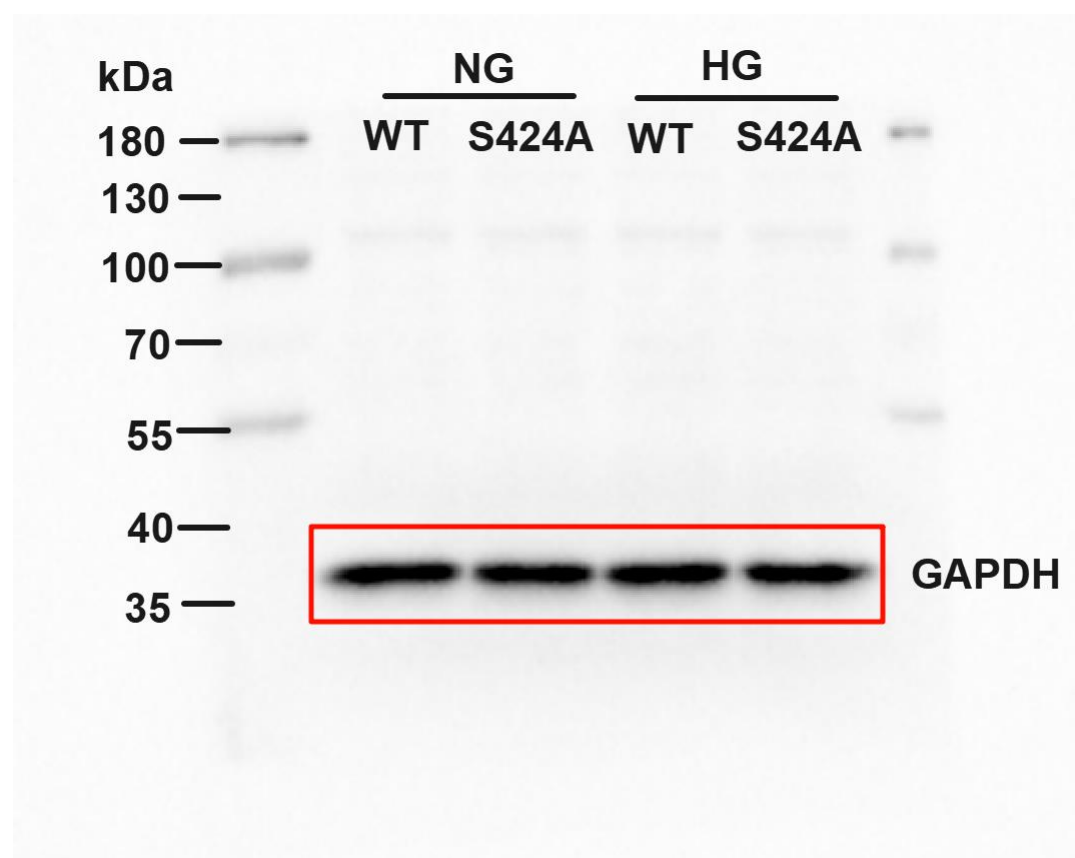

Supplement: Supplementary file 5 — Source data Fig. 3 [file 44318_2024_263_MOESM5_ESM.zip › Figure 3/3G/3G Western blot description for cropped image.pdf]

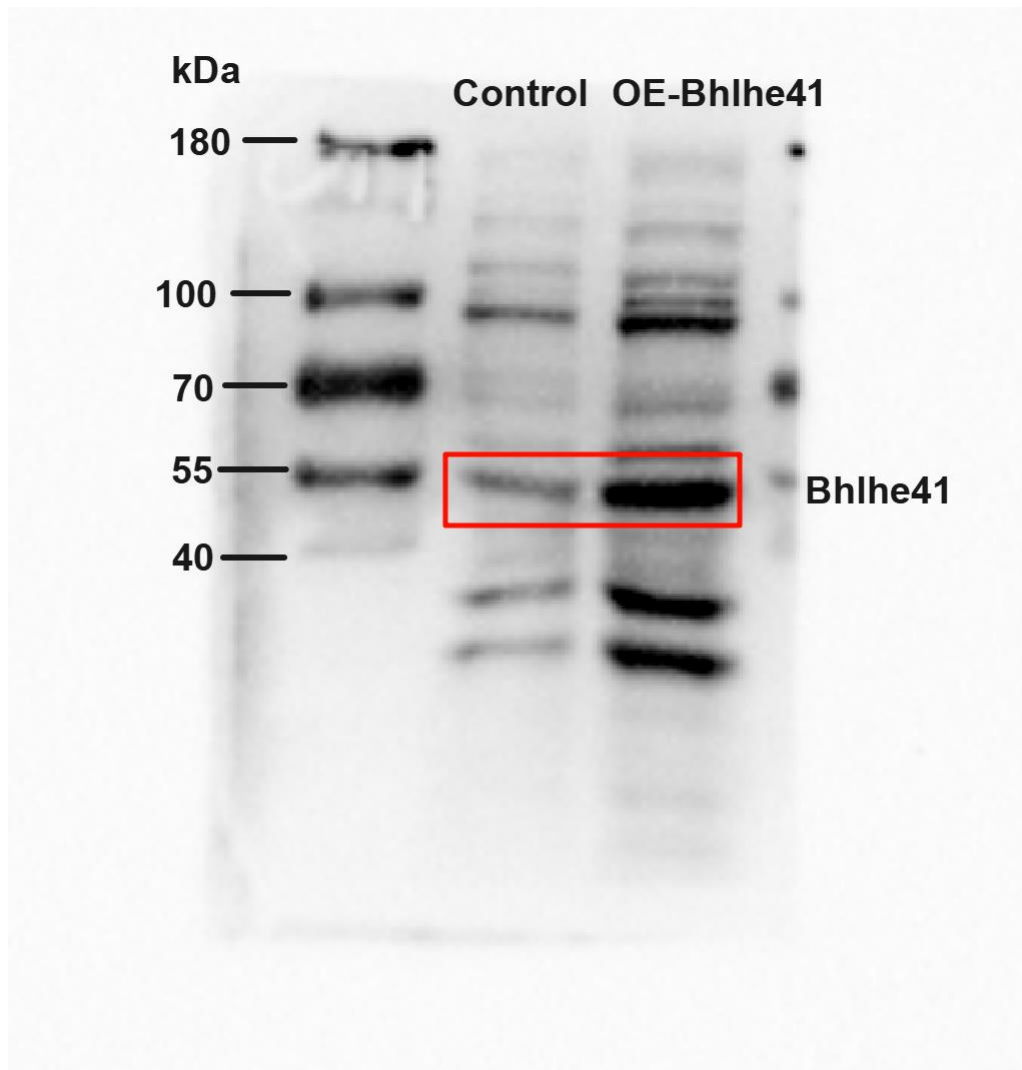

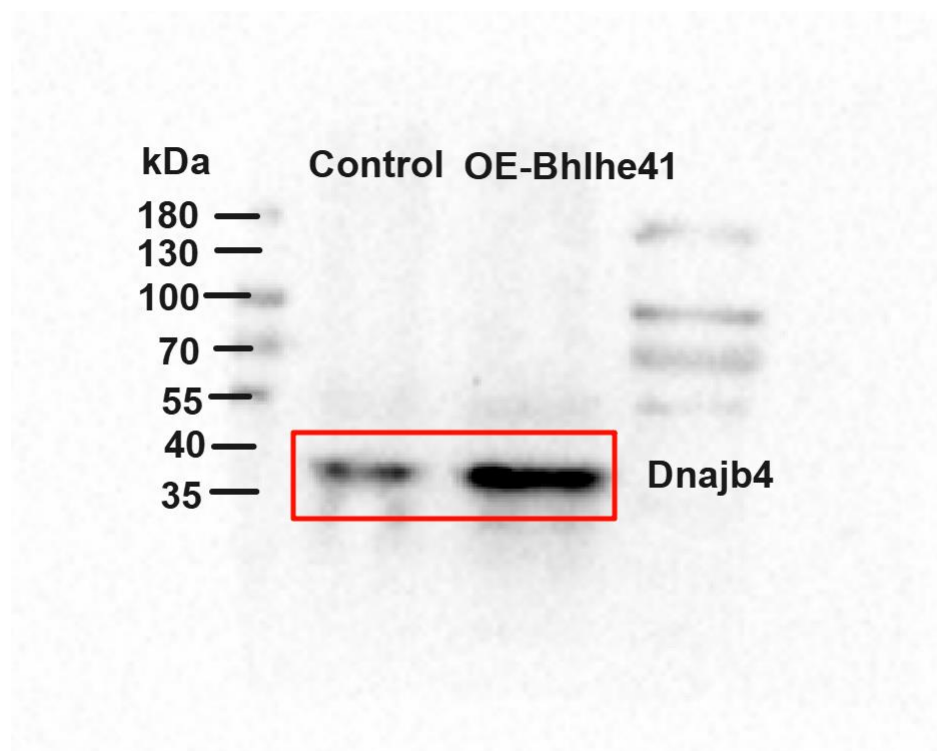

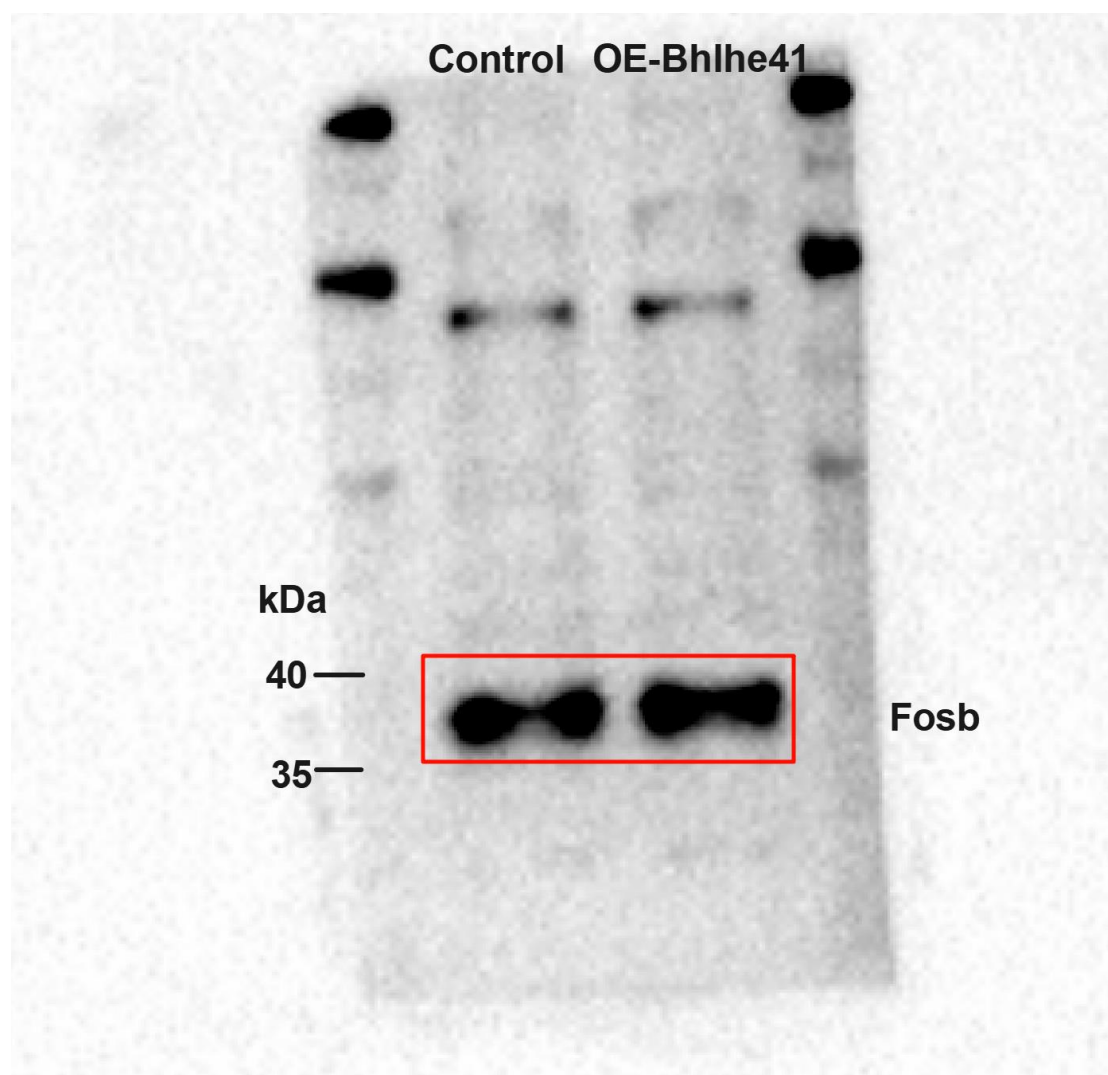

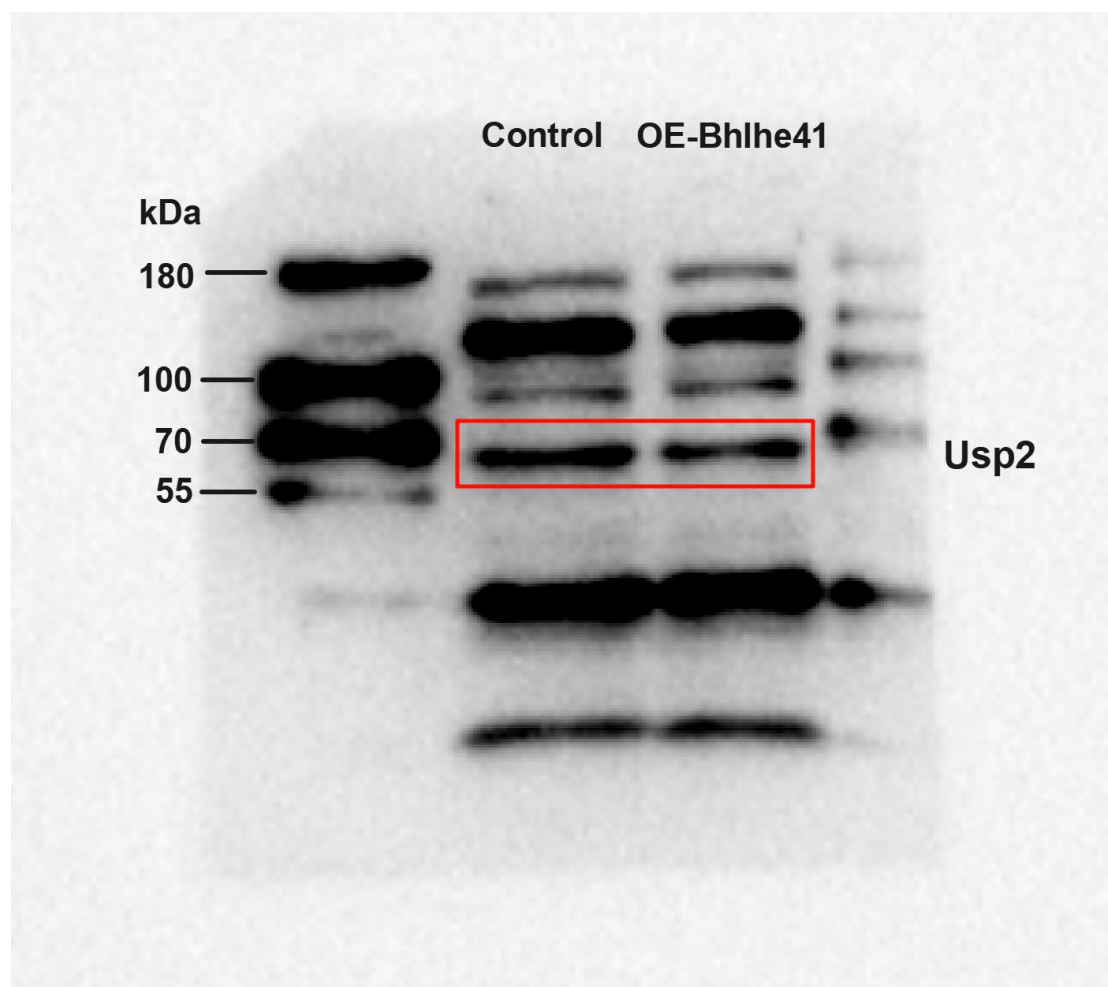

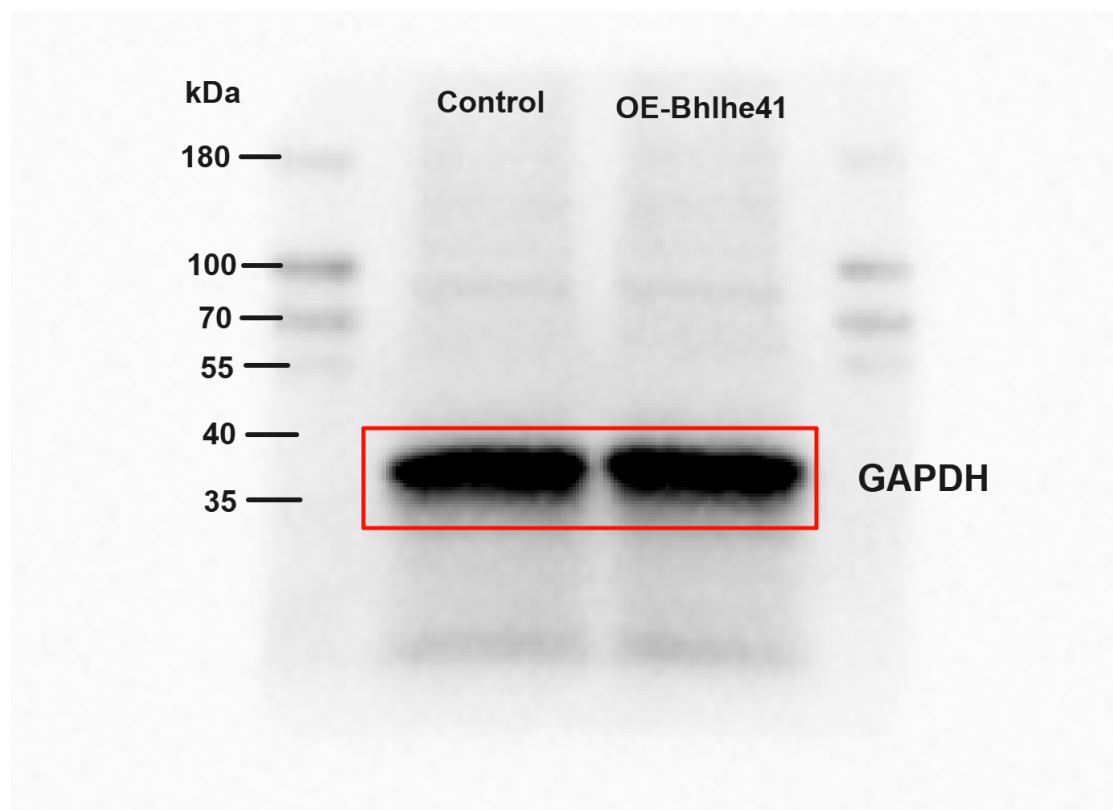

Supplement: Supplementary file 6 — Source data Fig. 4 [file 44318_2024_263_MOESM6_ESM.zip › Figure 4/4E/4E Western blot description for cropped image.pdf]

↵

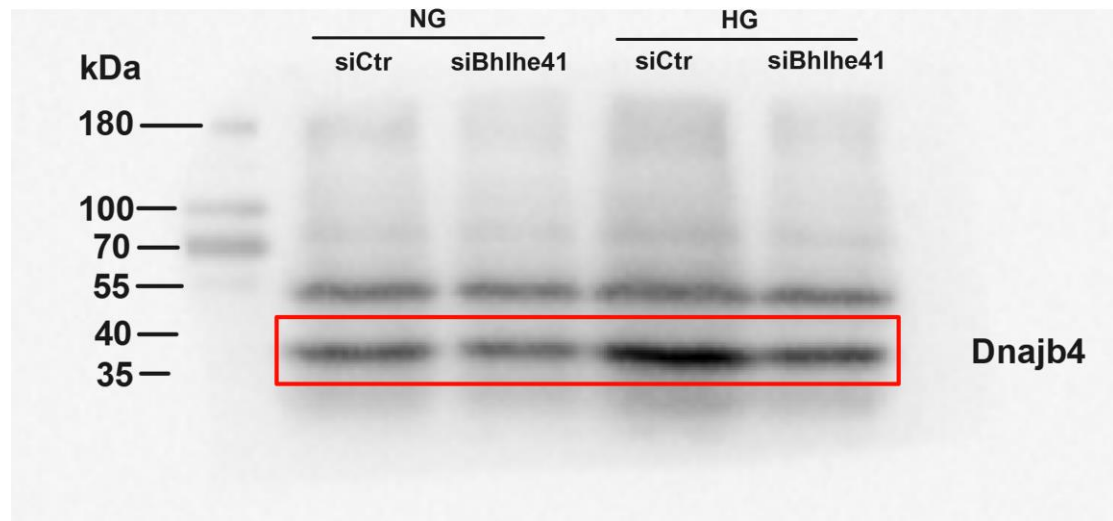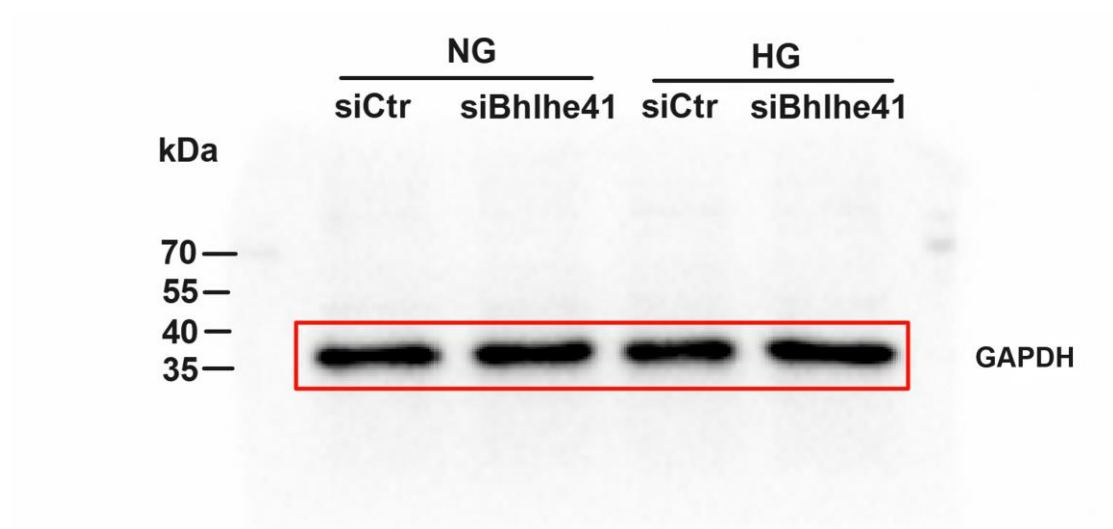

Supplement: Supplementary file 6 — Source data Fig. 4 [file 44318_2024_263_MOESM6_ESM.zip › Figure 4/4I/4I Western blot description for cropped image.pdf]

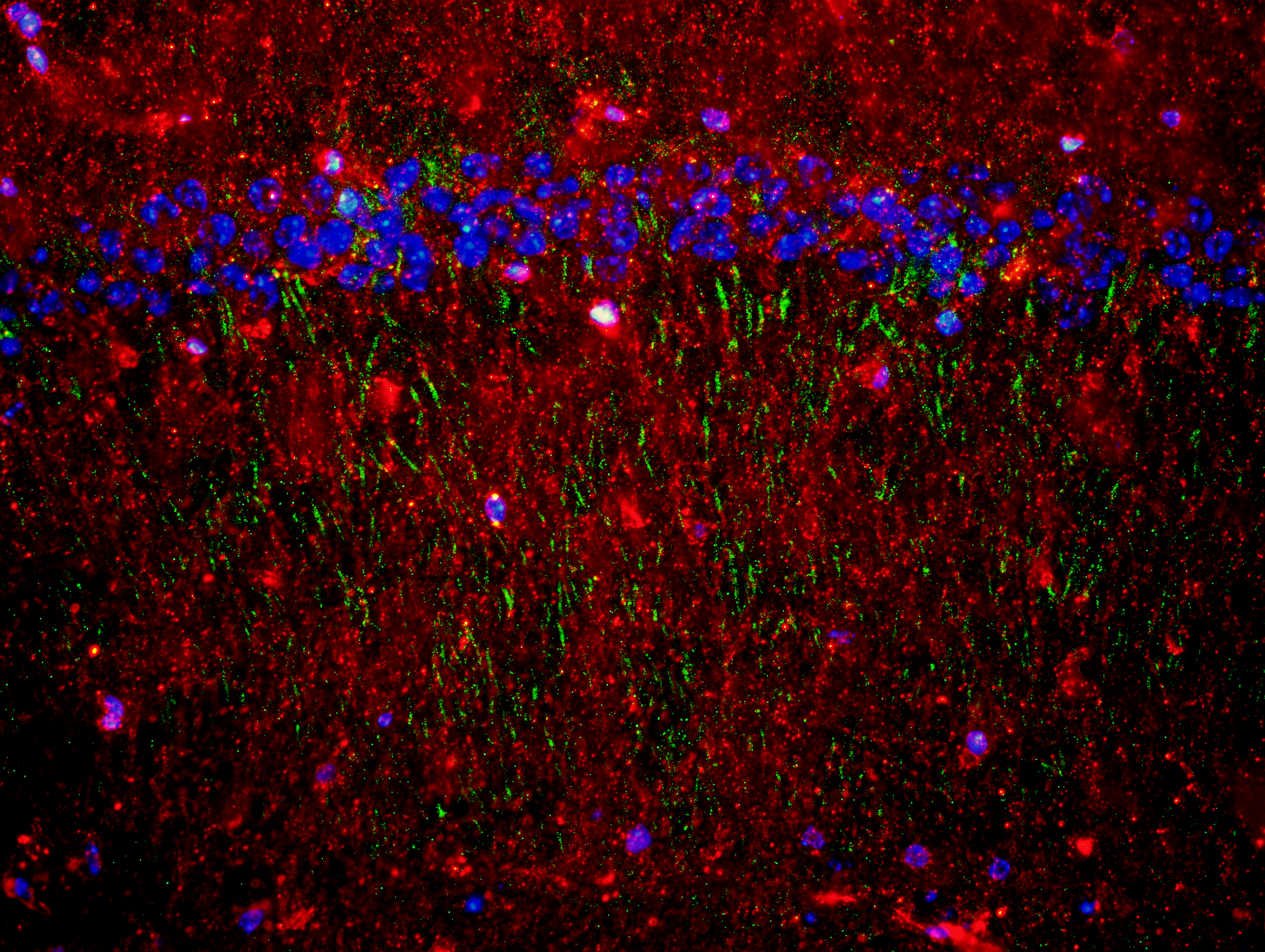

Supplement: Supplementary file 6 — Source data Fig. 4 [file 44318_2024_263_MOESM6_ESM.zip › Figure 4/4G/Fig 4G Immunofluorescence image/dbm+Bhlhe41cKO/dbm+Bhlhe41cKO-Merge.tif]

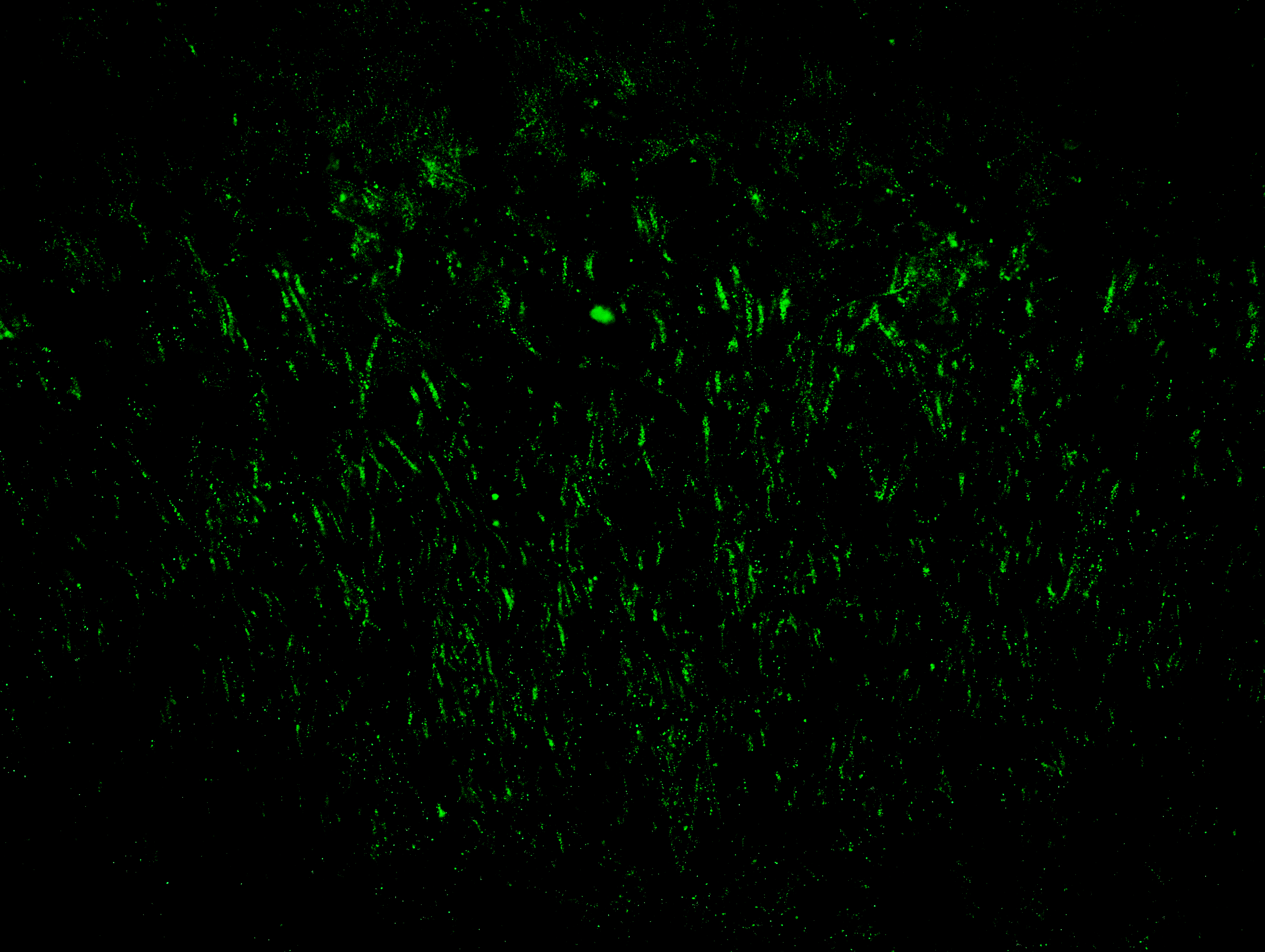

Supplement: Supplementary file 6 — Source data Fig. 4 [file 44318_2024_263_MOESM6_ESM.zip › Figure 4/4G/Fig 4G Immunofluorescence image/dbm+Bhlhe41cKO/dbm+Bhlhe41cKO-Dnajb4.tif]

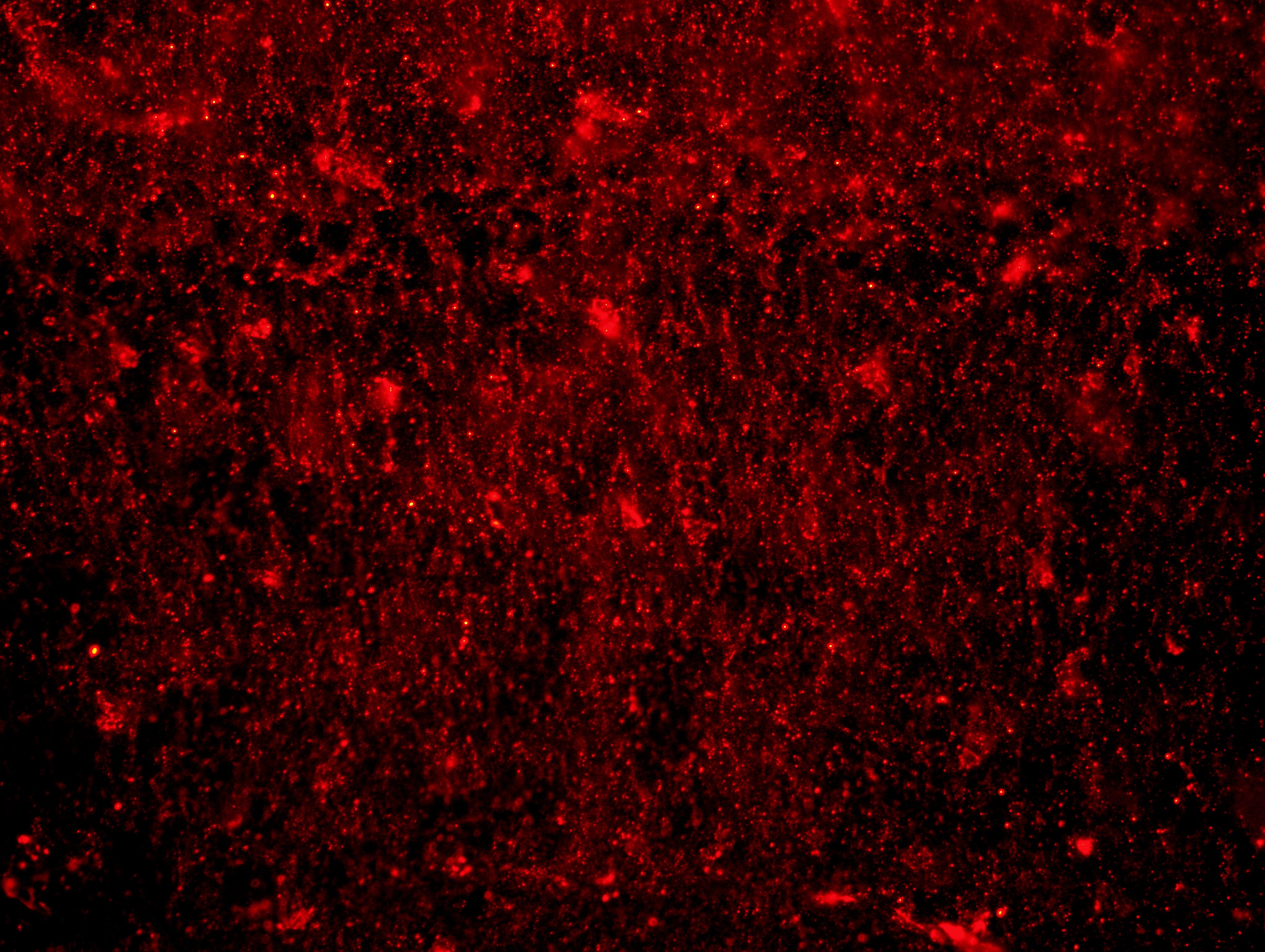

Supplement: Supplementary file 6 — Source data Fig. 4 [file 44318_2024_263_MOESM6_ESM.zip › Figure 4/4G/Fig 4G Immunofluorescence image/dbm+Bhlhe41cKO/dbm+Bhlhe41cKO-Map2.tif]

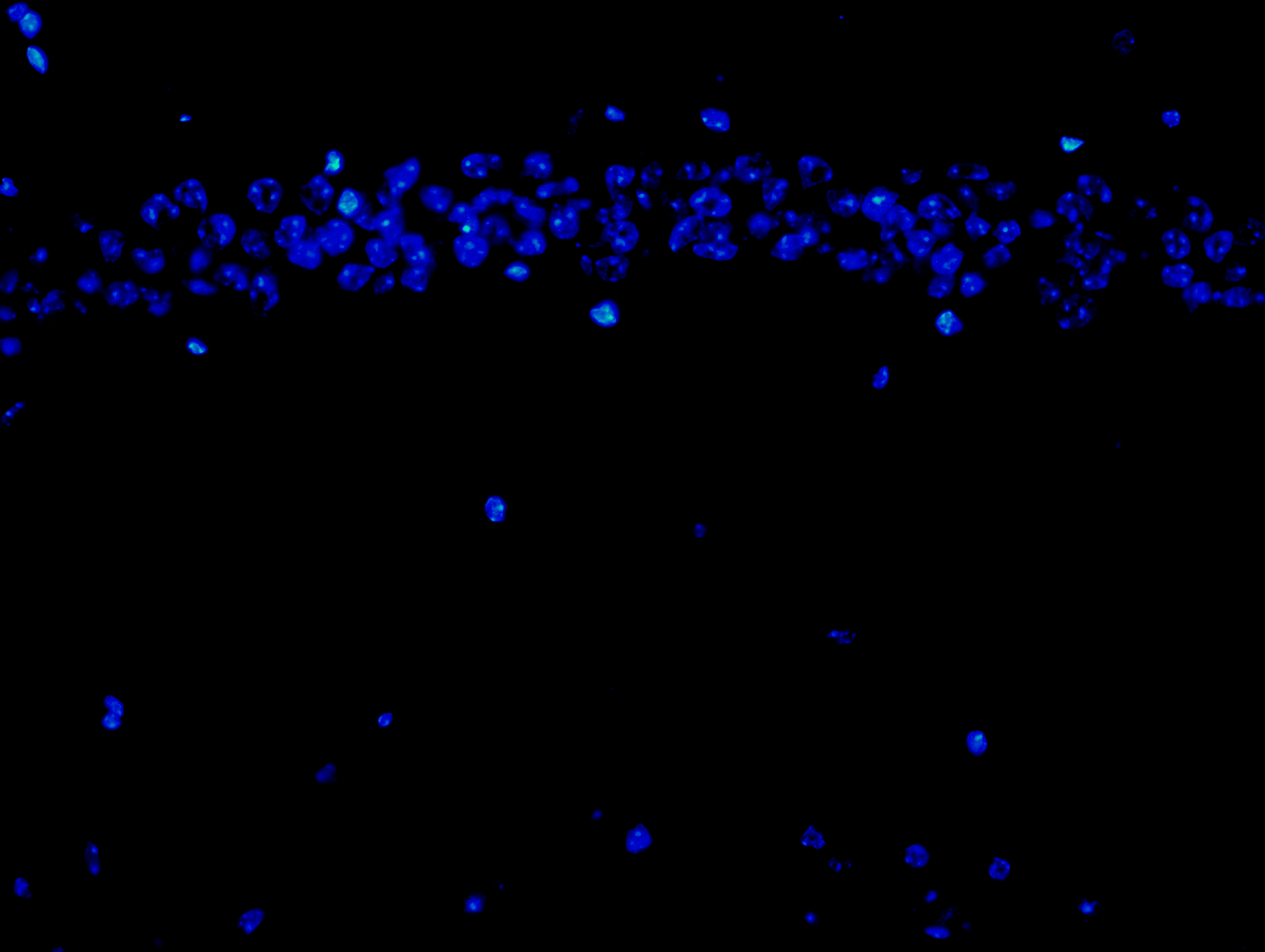

Supplement: Supplementary file 6 — Source data Fig. 4 [file 44318_2024_263_MOESM6_ESM.zip › Figure 4/4G/Fig 4G Immunofluorescence image/dbm+Bhlhe41cKO/dbm+Bhlhe41cKO-DAPI.tif]

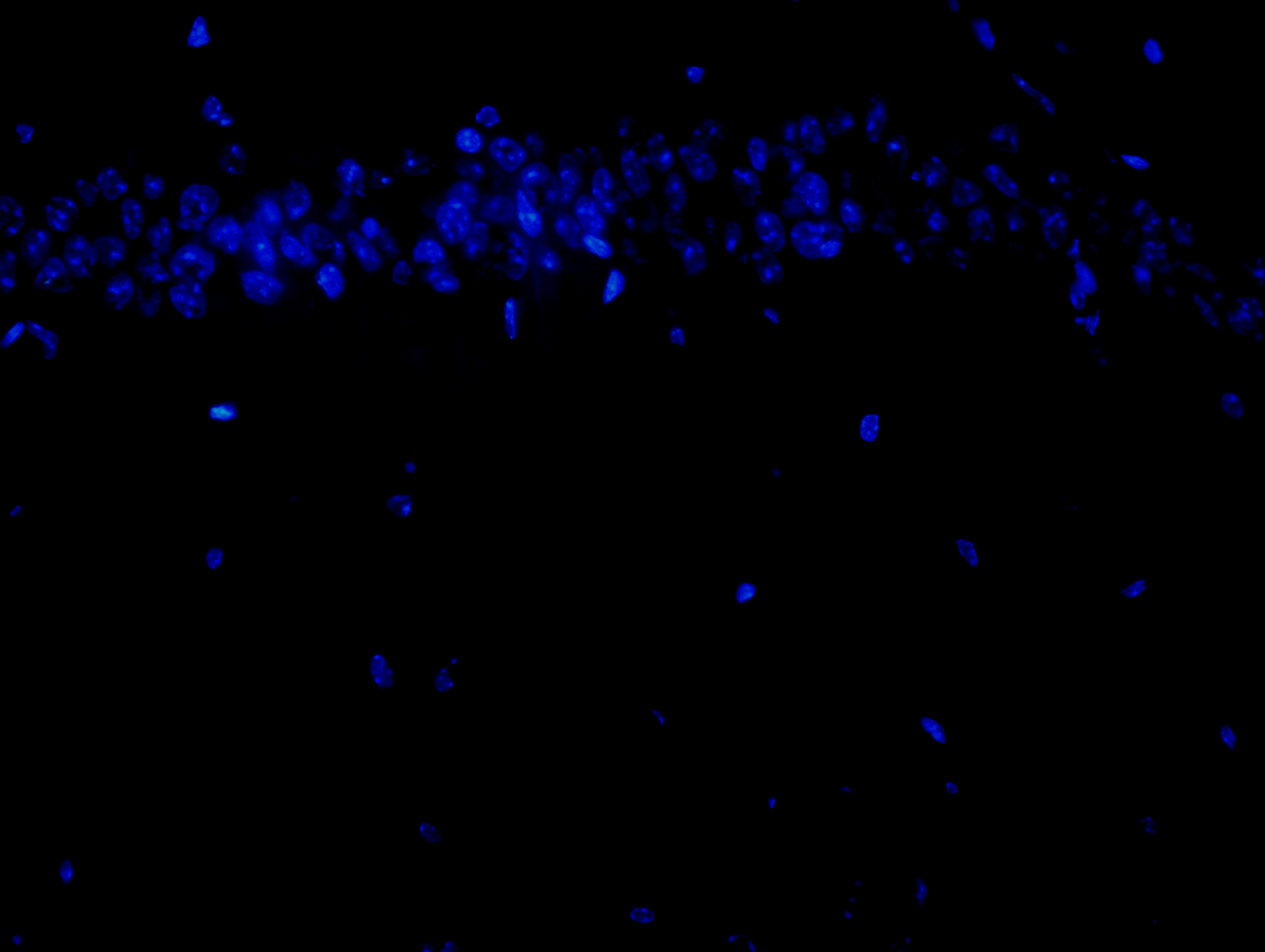

Supplement: Supplementary file 6 — Source data Fig. 4 [file 44318_2024_263_MOESM6_ESM.zip › Figure 4/4G/Fig 4G Immunofluorescence image/dbm+Bhlhe41ff/dbm+Bhlhe41flfl-DAPI.tif]

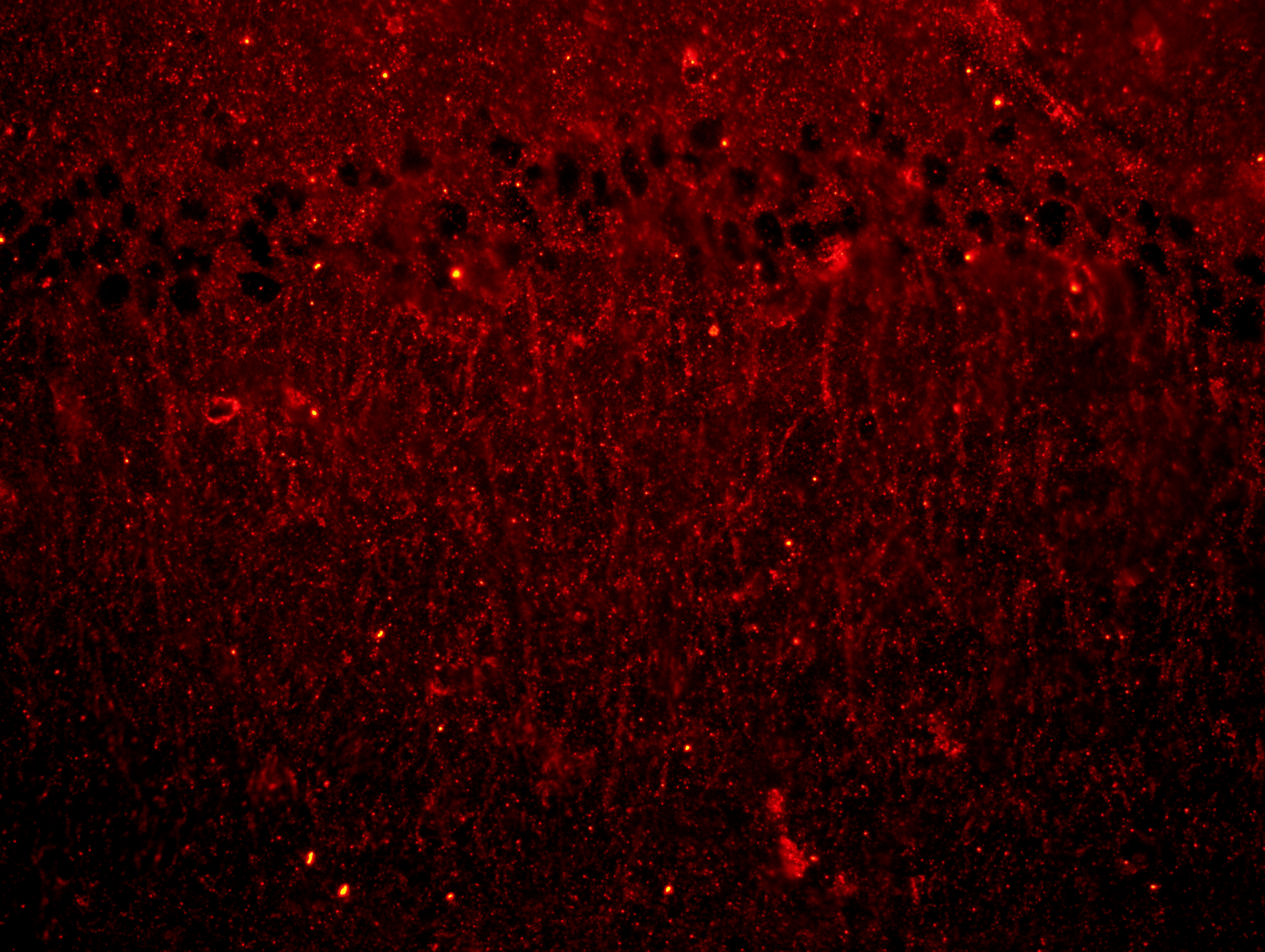

Supplement: Supplementary file 6 — Source data Fig. 4 [file 44318_2024_263_MOESM6_ESM.zip › Figure 4/4G/Fig 4G Immunofluorescence image/dbm+Bhlhe41ff/dbm+Bhlhe41flfl-Map2.tif]

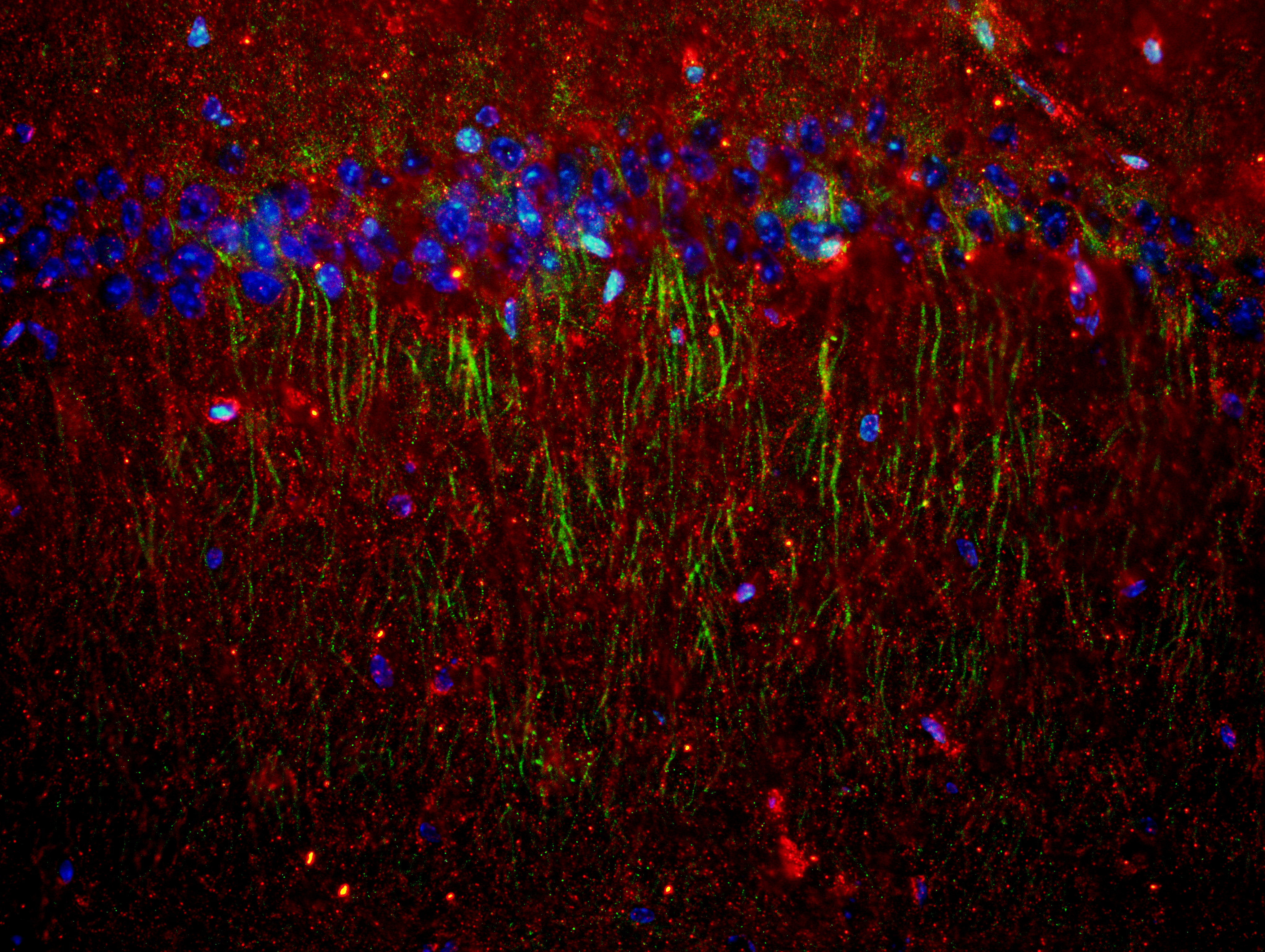

Supplement: Supplementary file 6 — Source data Fig. 4 [file 44318_2024_263_MOESM6_ESM.zip › Figure 4/4G/Fig 4G Immunofluorescence image/dbm+Bhlhe41ff/dbm+Bhlhe41flfl-Dnajb4.tif]

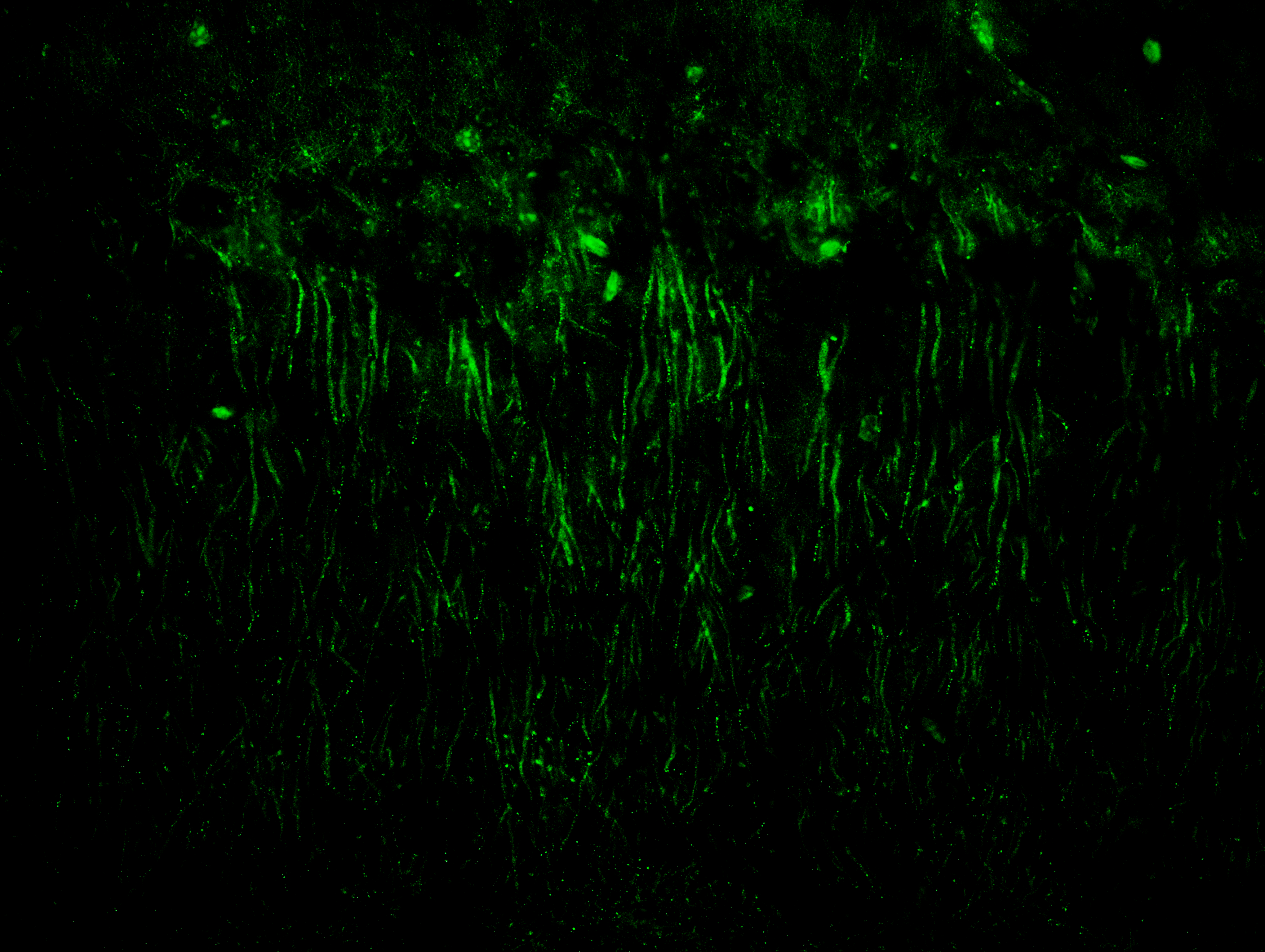

Supplement: Supplementary file 6 — Source data Fig. 4 [file 44318_2024_263_MOESM6_ESM.zip › Figure 4/4G/Fig 4G Immunofluorescence image/dbm+Bhlhe41ff/dbm+Bhlhe41flfl-Merge.tif]

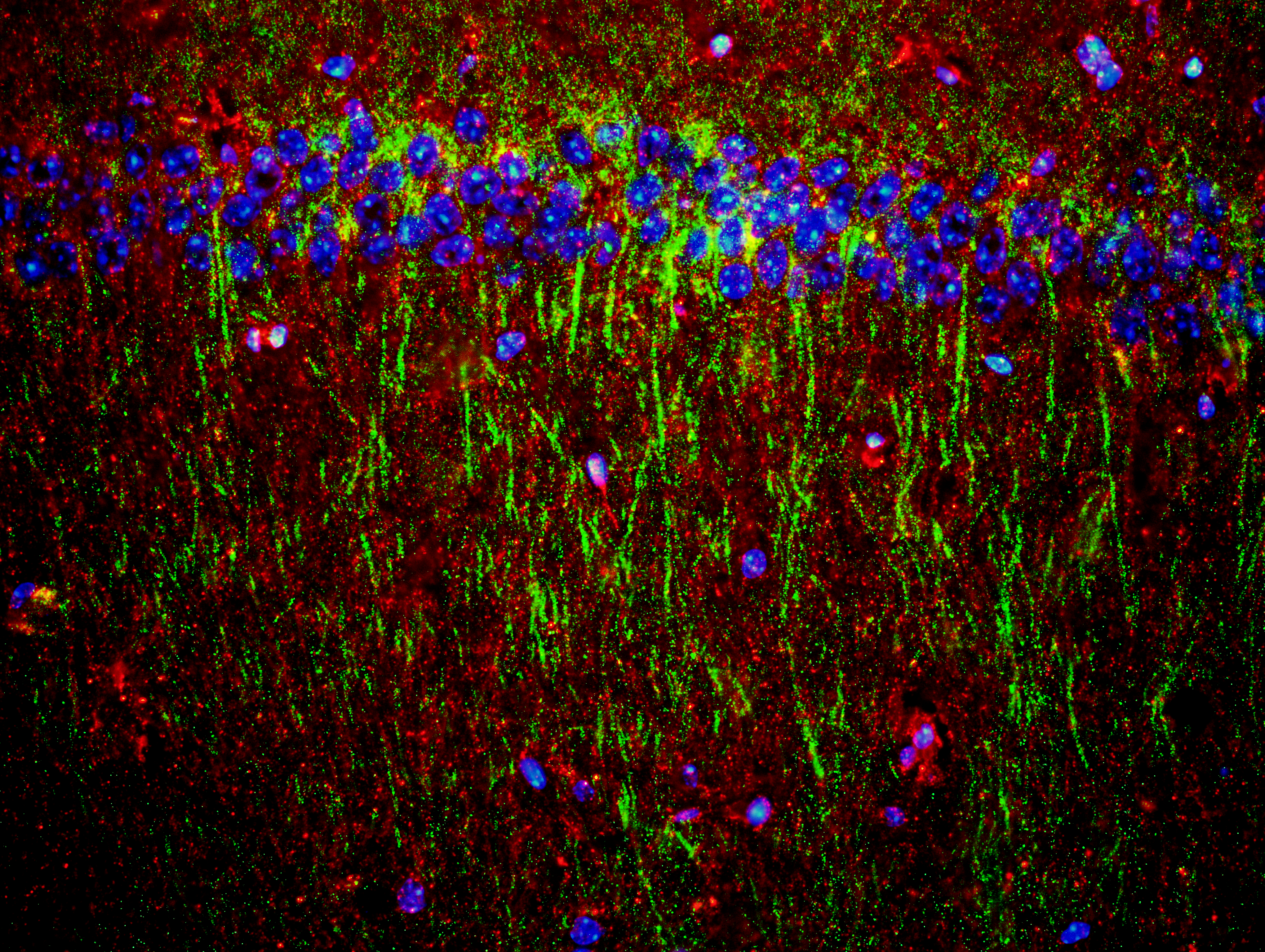

Supplement: Supplementary file 6 — Source data Fig. 4 [file 44318_2024_263_MOESM6_ESM.zip › Figure 4/4G/Fig 4G Immunofluorescence image/dbdb+Bhlhe41ff/dbdb+Bhlhe41flfl-Merge.tif]

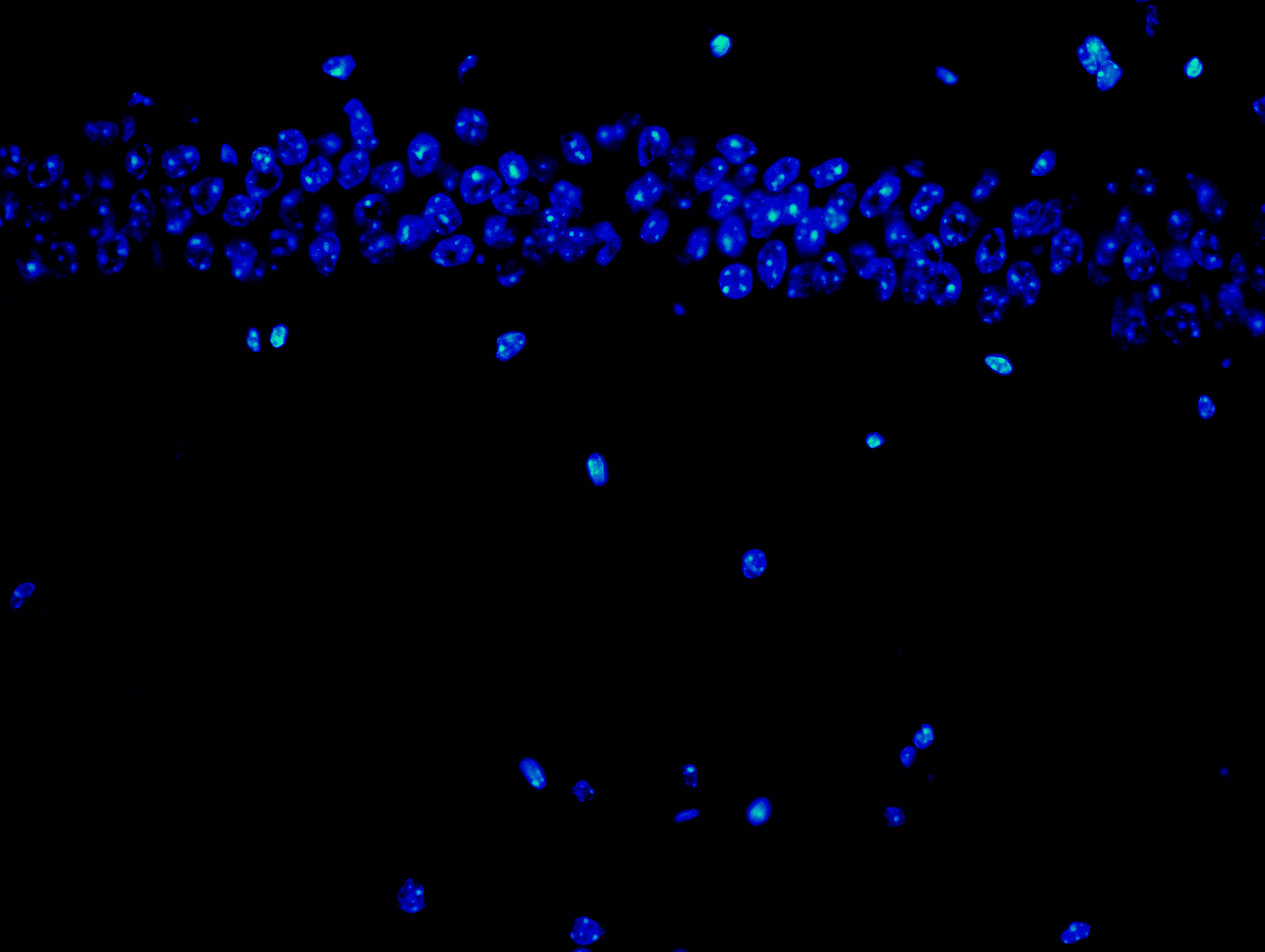

Supplement: Supplementary file 6 — Source data Fig. 4 [file 44318_2024_263_MOESM6_ESM.zip › Figure 4/4G/Fig 4G Immunofluorescence image/dbdb+Bhlhe41ff/dbdb+Bhlhe41flfl-DAPI.tif]

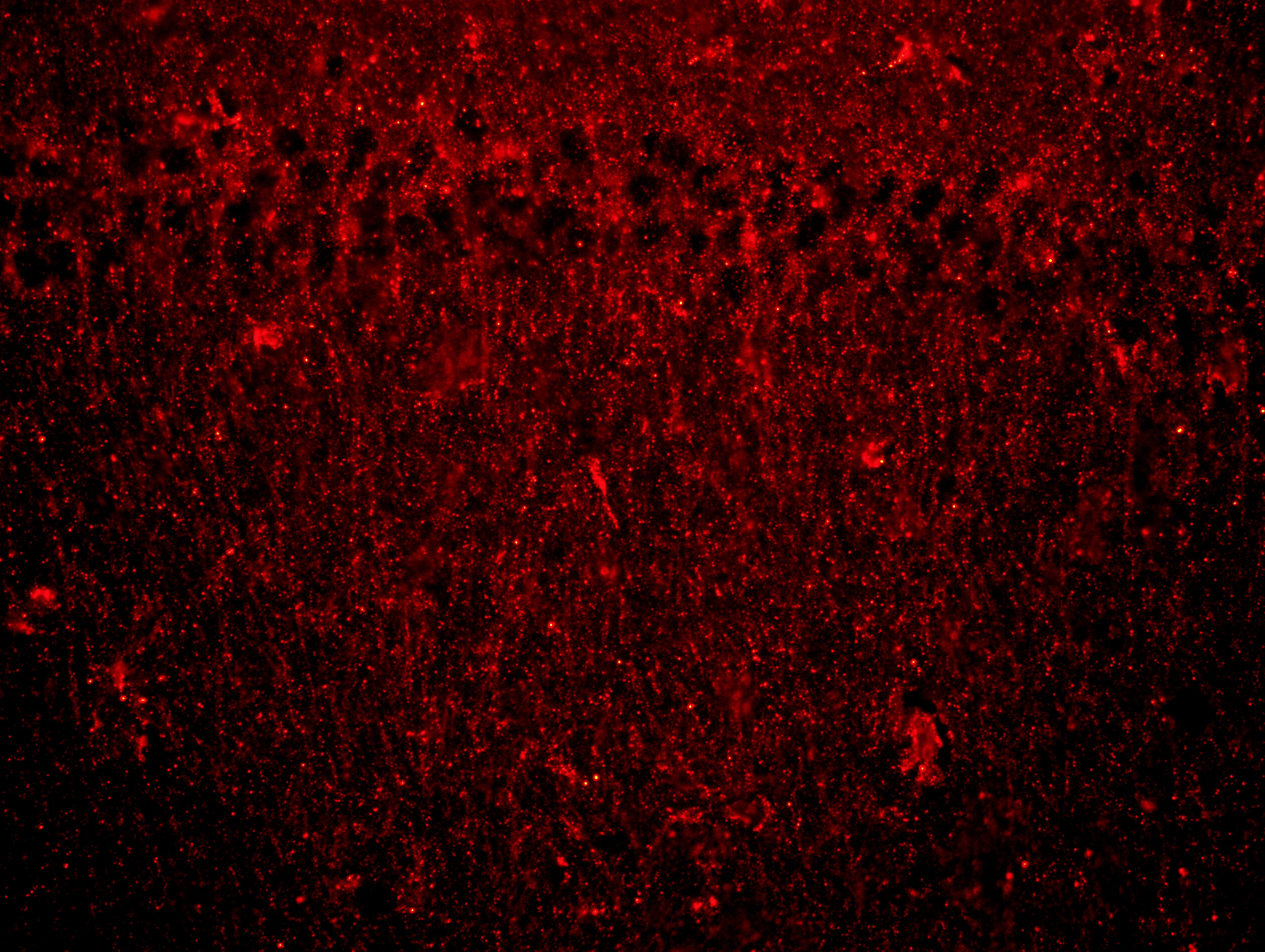

Supplement: Supplementary file 6 — Source data Fig. 4 [file 44318_2024_263_MOESM6_ESM.zip › Figure 4/4G/Fig 4G Immunofluorescence image/dbdb+Bhlhe41ff/dbdb+Bhlhe41flfl-Map2.tif]

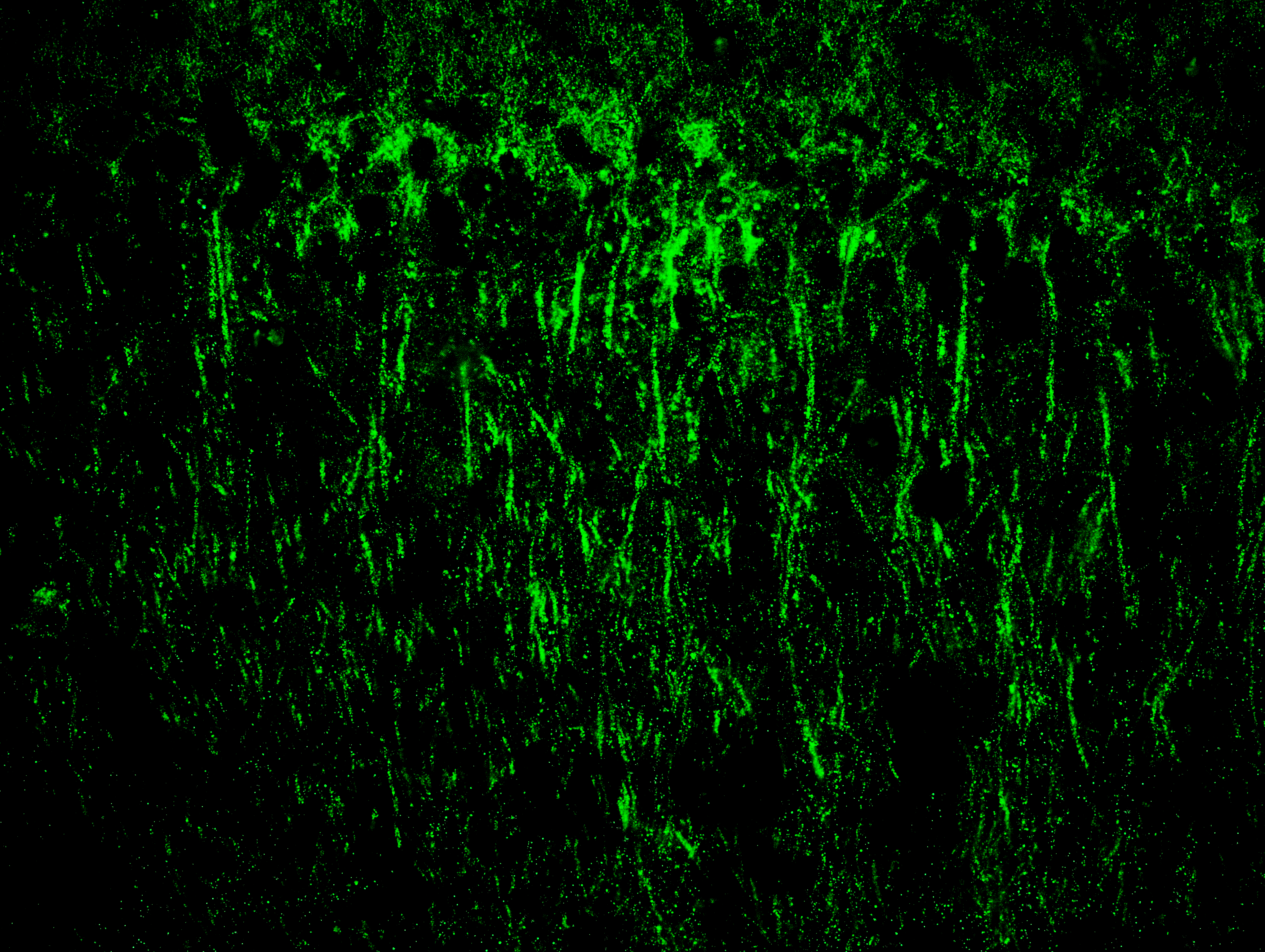

Supplement: Supplementary file 6 — Source data Fig. 4 [file 44318_2024_263_MOESM6_ESM.zip › Figure 4/4G/Fig 4G Immunofluorescence image/dbdb+Bhlhe41ff/dbdb+Bhlhe41flfl-Dnajb4.tif]

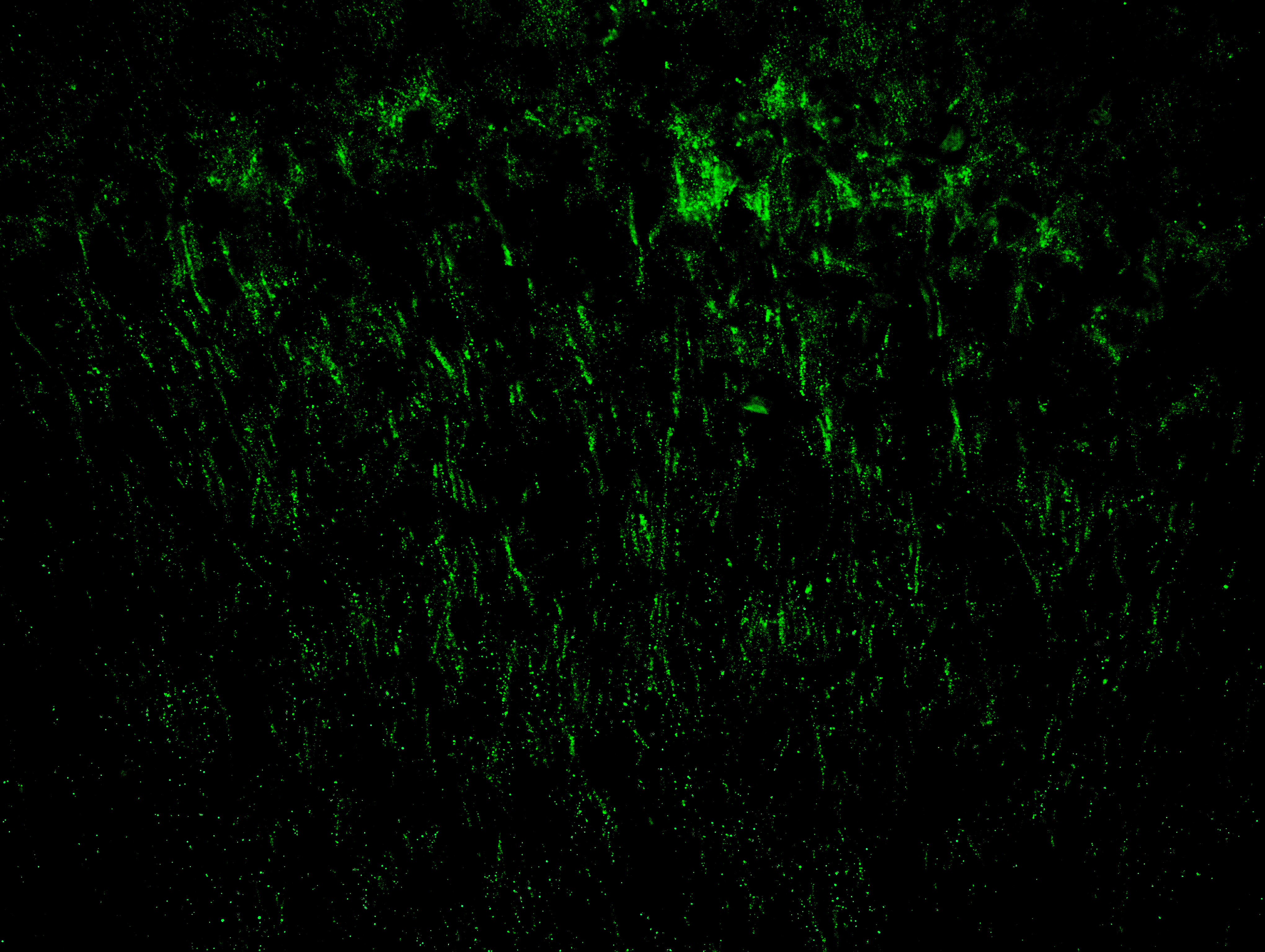

Supplement: Supplementary file 6 — Source data Fig. 4 [file 44318_2024_263_MOESM6_ESM.zip › Figure 4/4G/Fig 4G Immunofluorescence image/dbdb+Bhlhe41cKO/dbdb+Bhlhe41cKO-Dnajb4.tif]

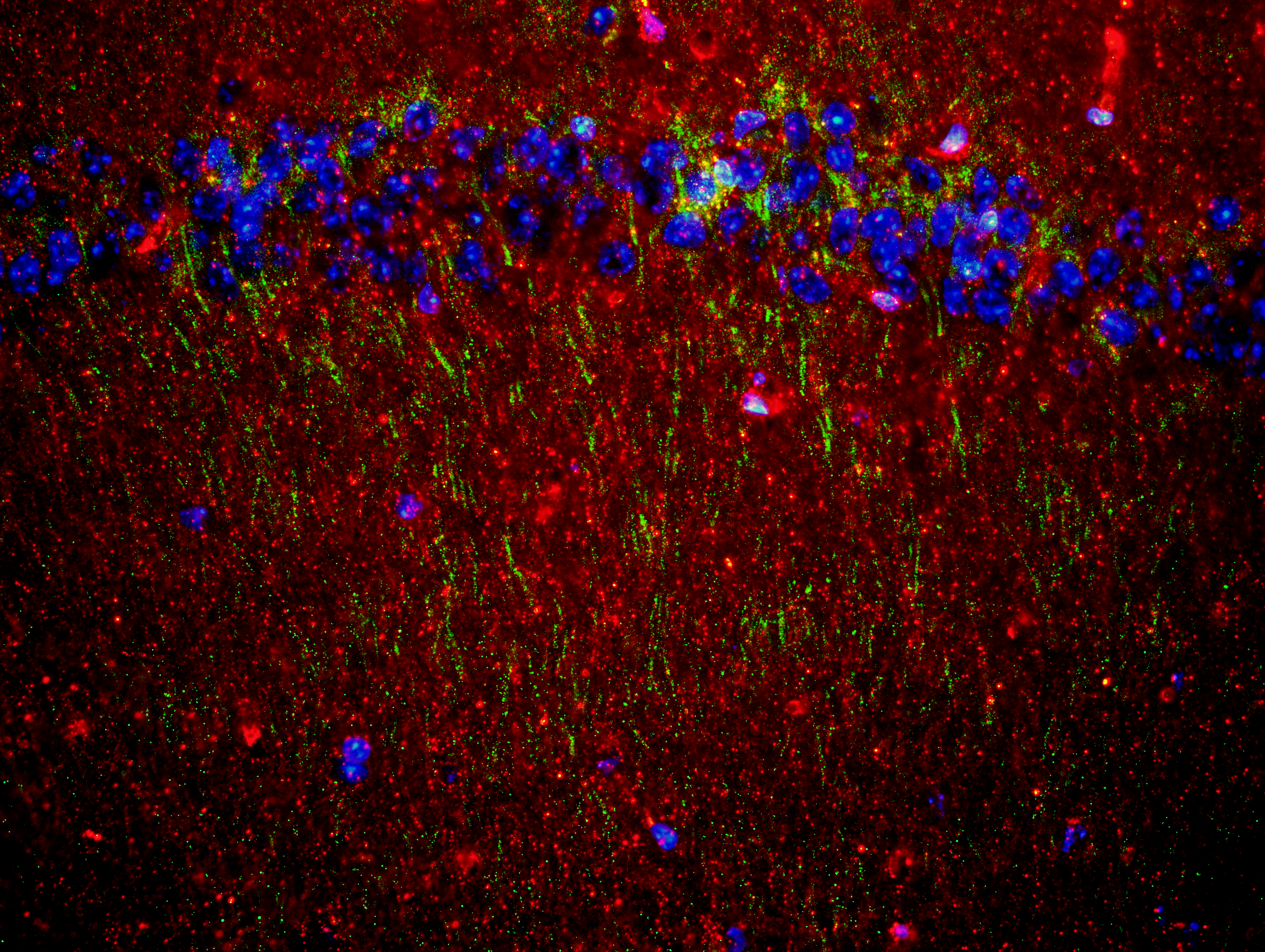

Supplement: Supplementary file 6 — Source data Fig. 4 [file 44318_2024_263_MOESM6_ESM.zip › Figure 4/4G/Fig 4G Immunofluorescence image/dbdb+Bhlhe41cKO/dbdb+Bhlhe41cKO-Merge.tif]

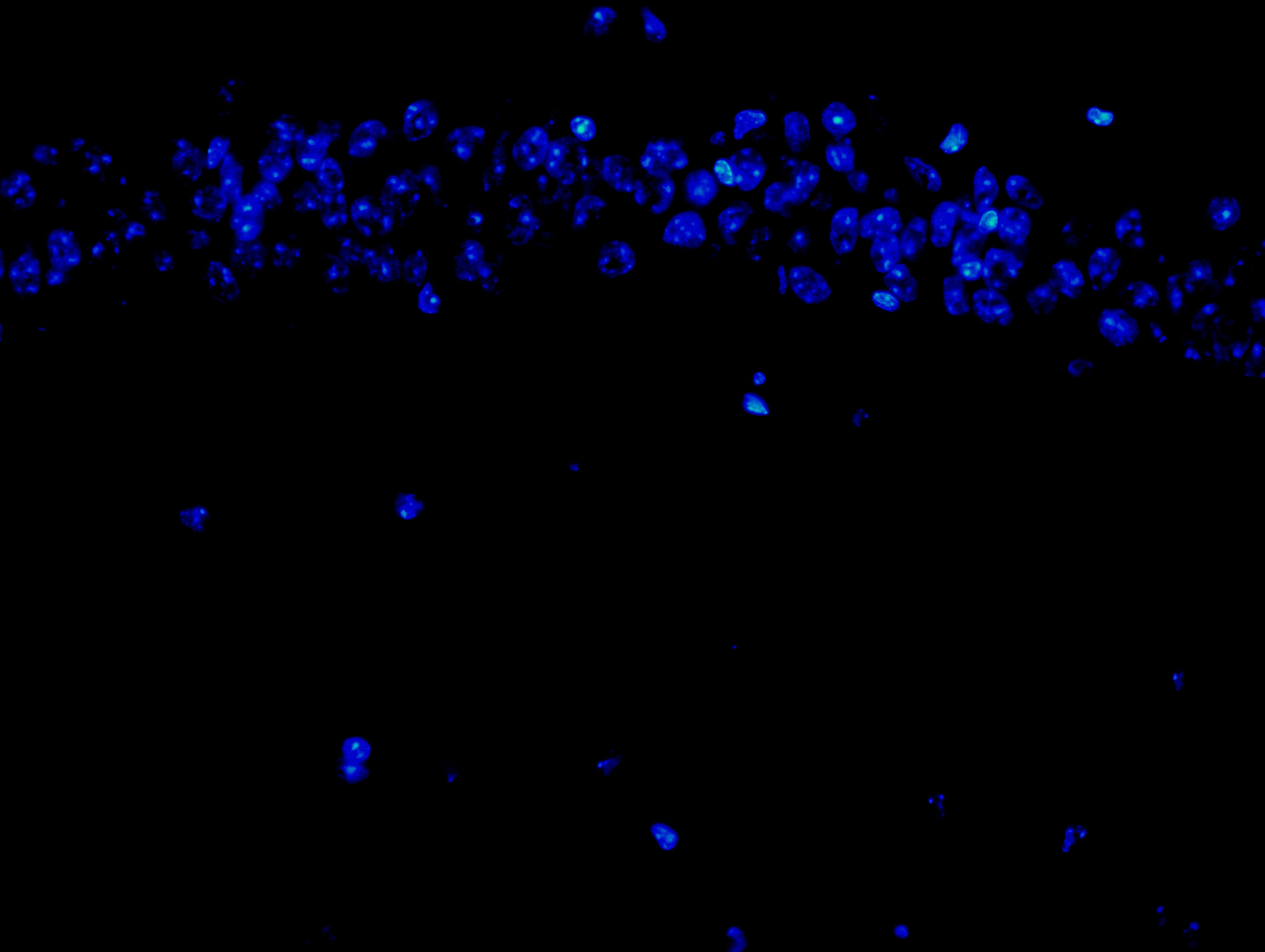

Supplement: Supplementary file 6 — Source data Fig. 4 [file 44318_2024_263_MOESM6_ESM.zip › Figure 4/4G/Fig 4G Immunofluorescence image/dbdb+Bhlhe41cKO/dbdb+Bhlhe41cKO-DAPI.tif]

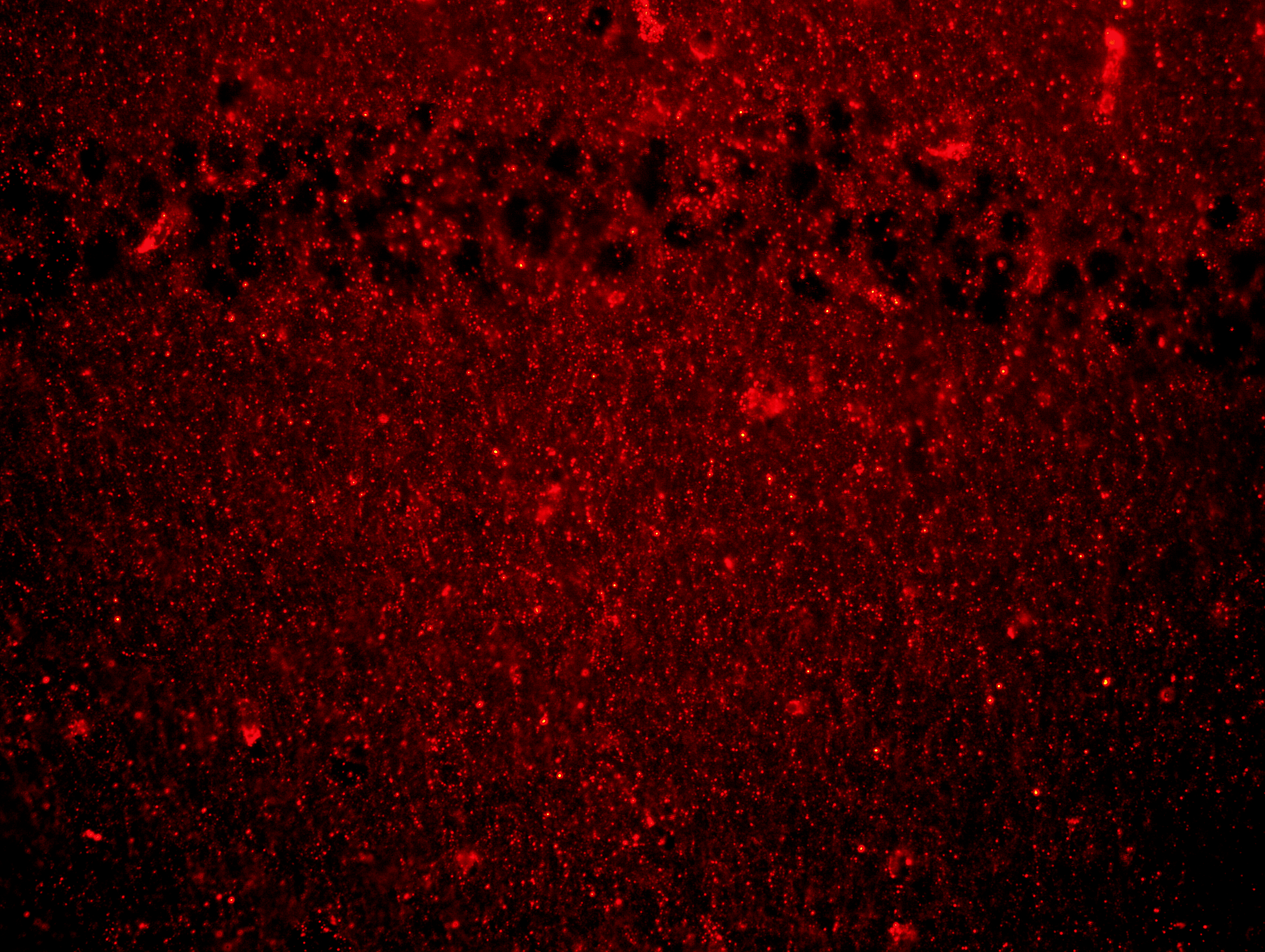

Supplement: Supplementary file 6 — Source data Fig. 4 [file 44318_2024_263_MOESM6_ESM.zip › Figure 4/4G/Fig 4G Immunofluorescence image/dbdb+Bhlhe41cKO/dbdb+Bhlhe41cKO-Map2.tif]

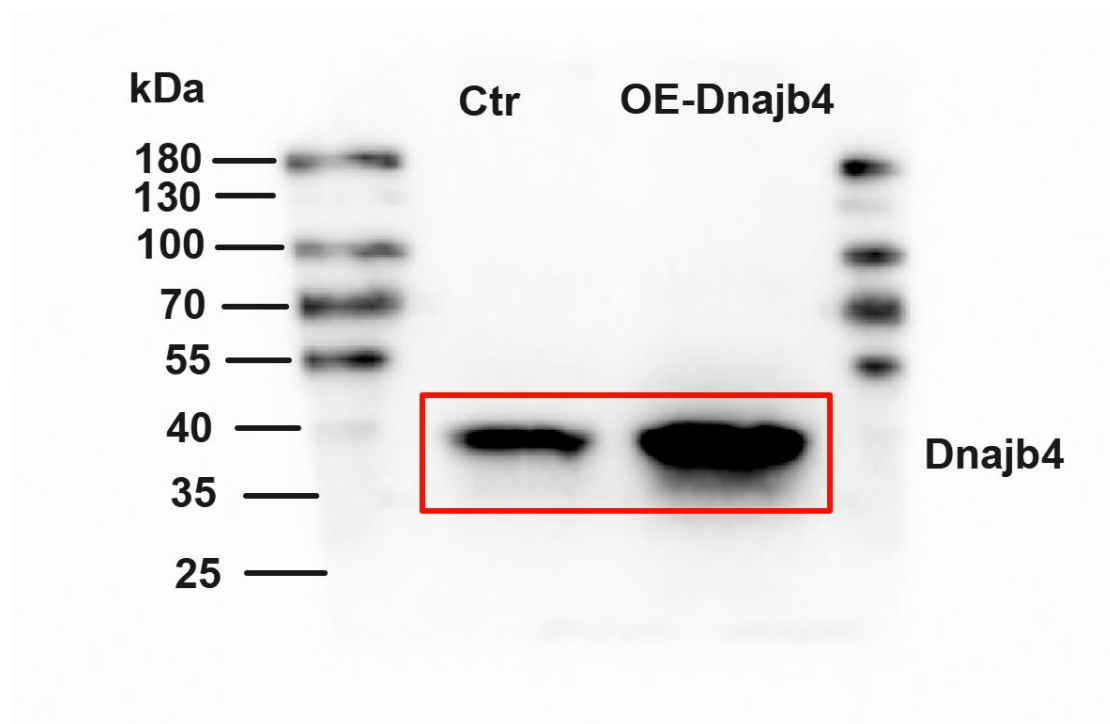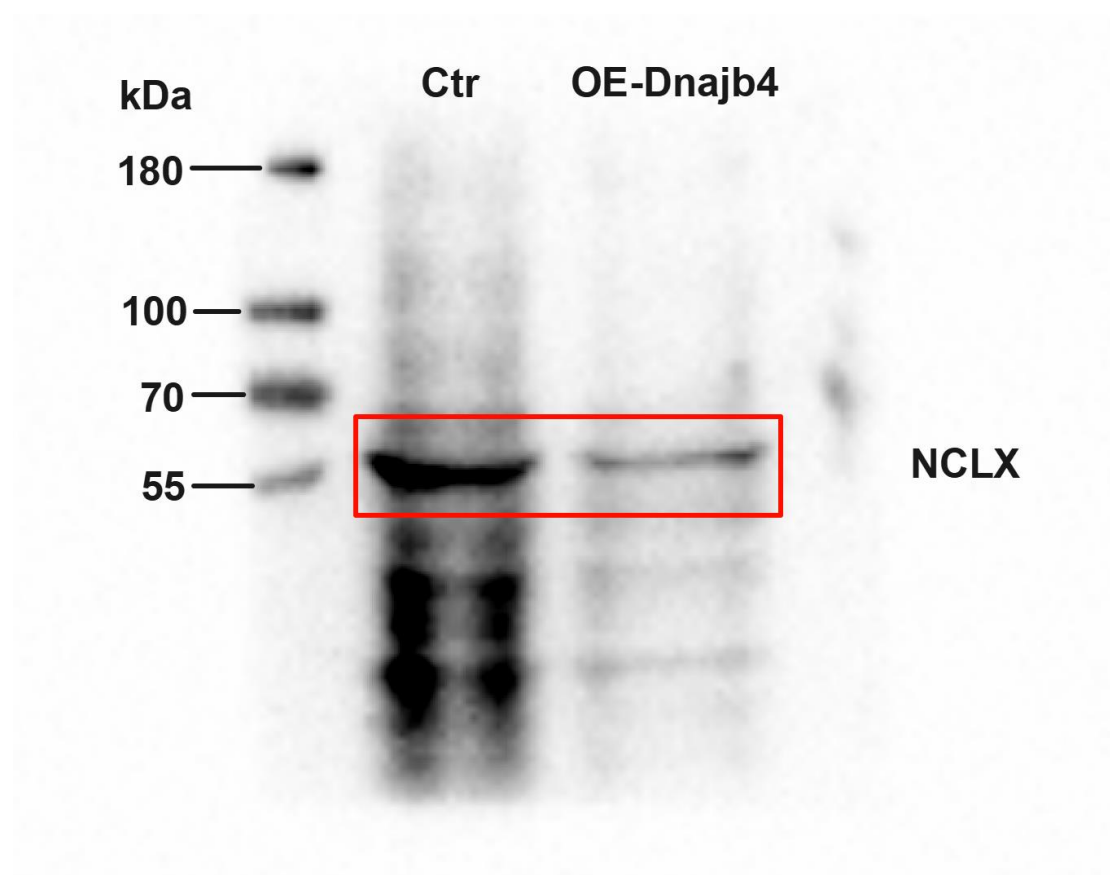

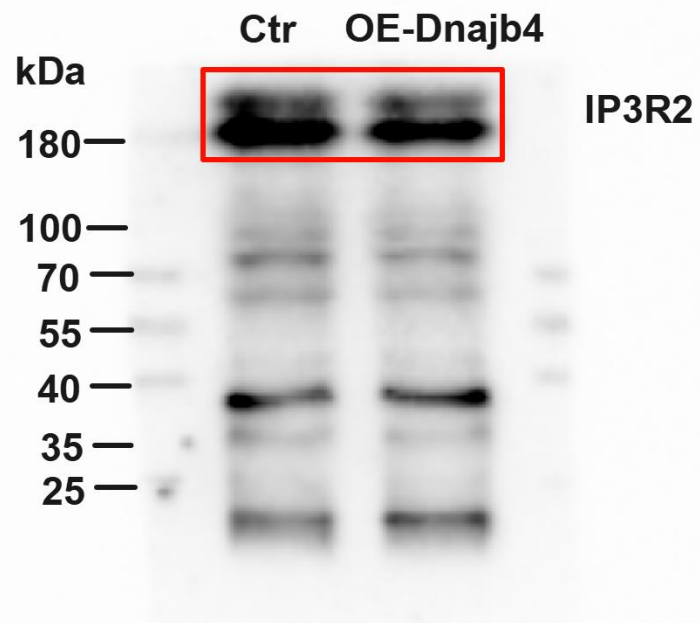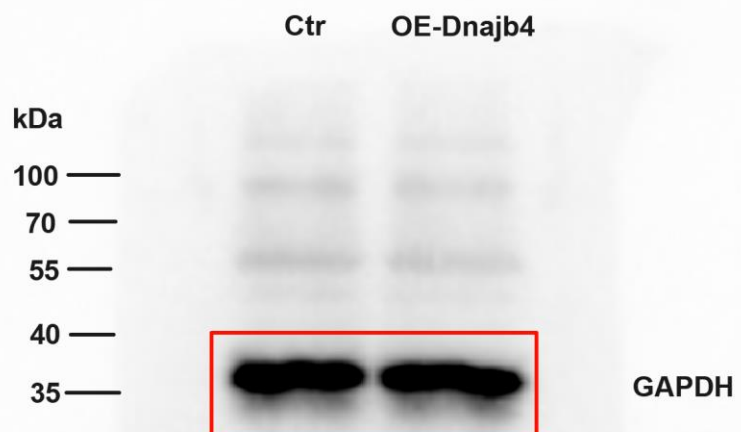

Supplement: Supplementary file 7 — Source data Fig. 5 [file 44318_2024_263_MOESM7_ESM.zip › Figure 5/5G/5G Western blot description for cropped image.pdf]

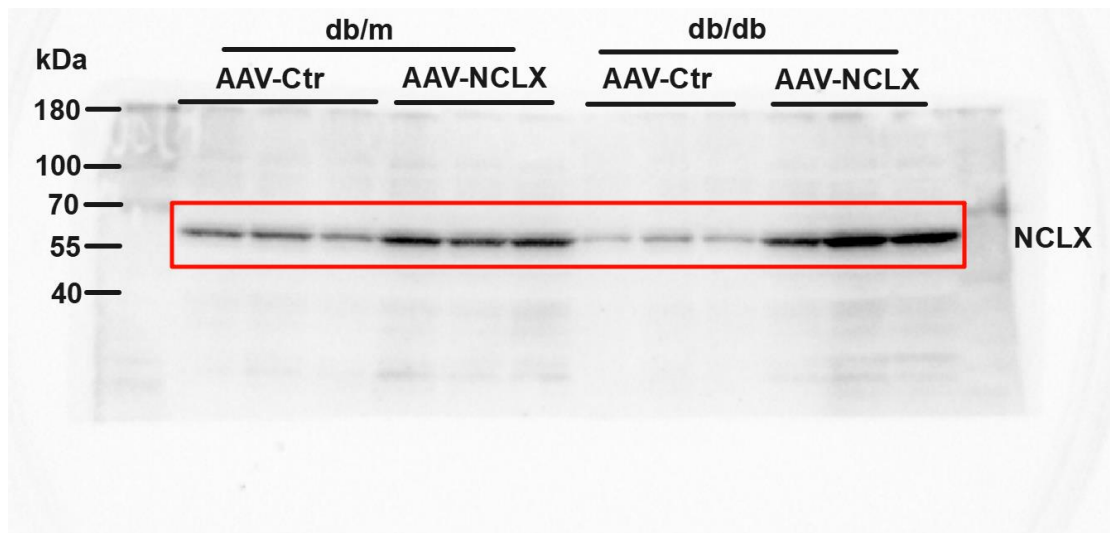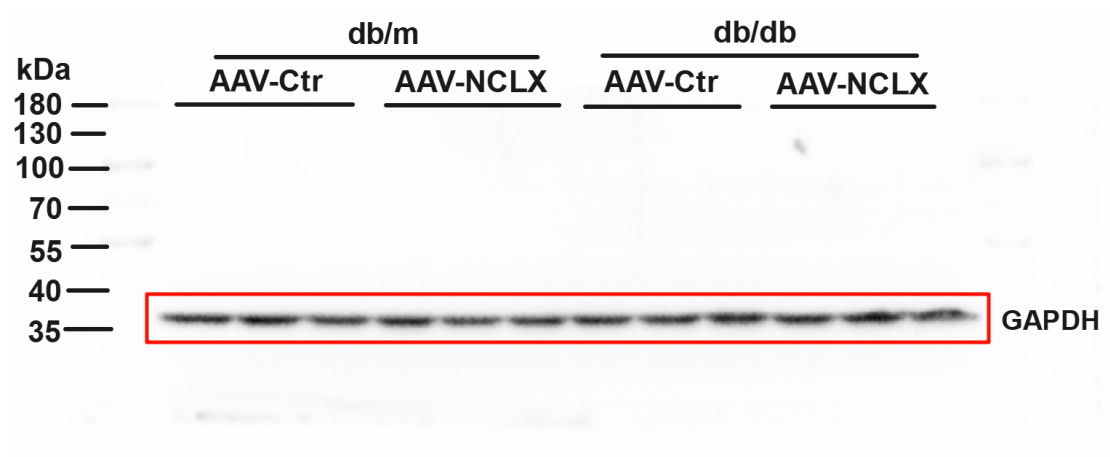

Supplement: Supplementary file 7 — Source data Fig. 5 [file 44318_2024_263_MOESM7_ESM.zip › Figure 5/5A/5A Western blot description for cropped image.pdf]

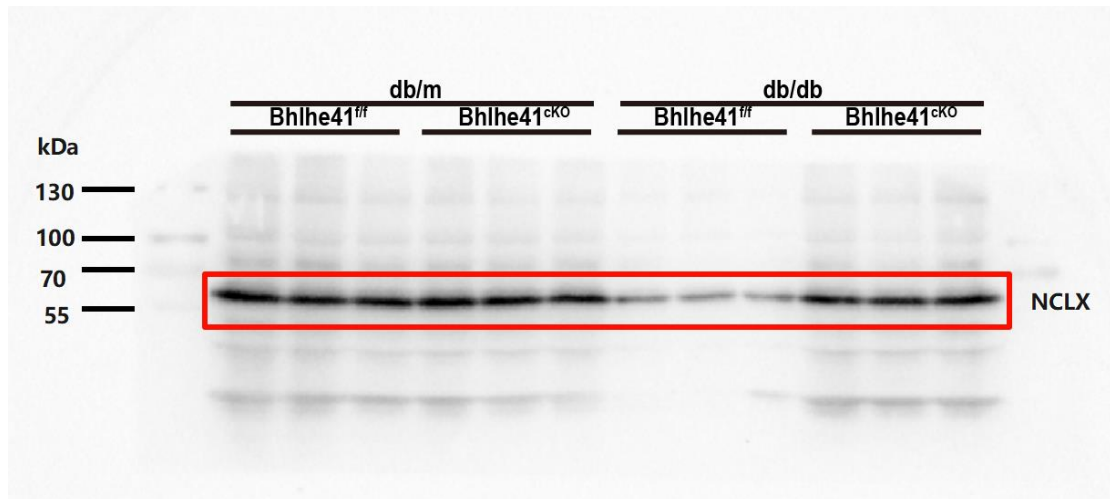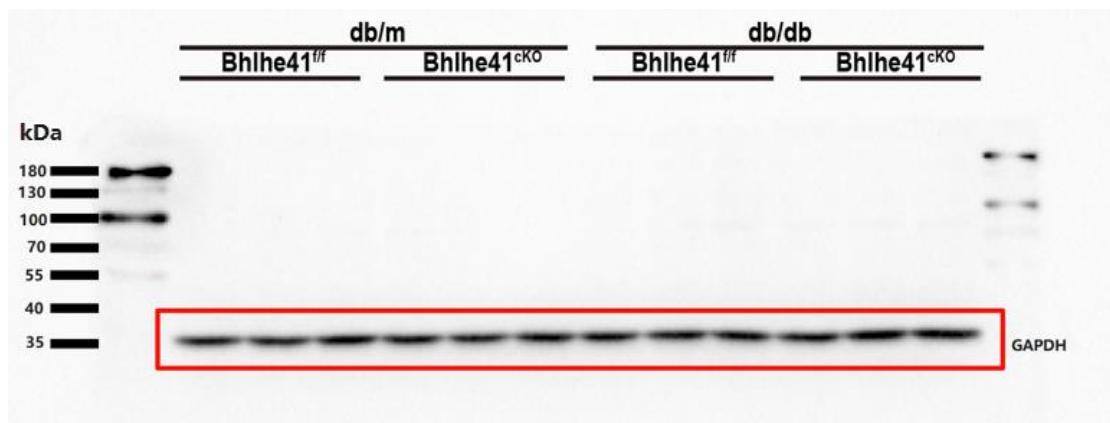

Supplement: Supplementary file 7 — Source data Fig. 5 [file 44318_2024_263_MOESM7_ESM.zip › Figure 5/5M/5M Western blot description for cropped image.pdf]

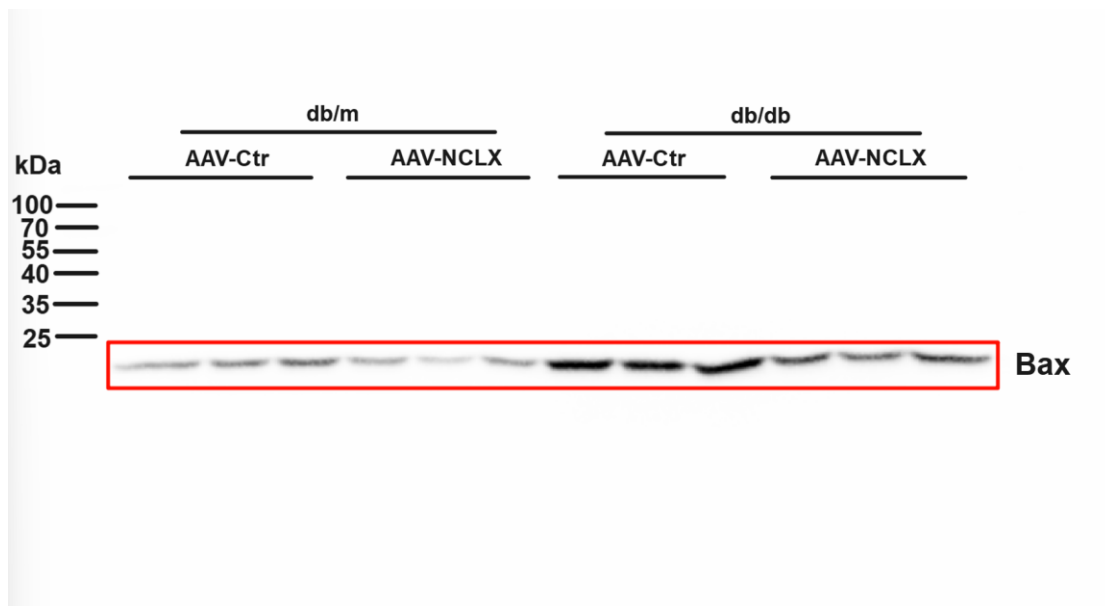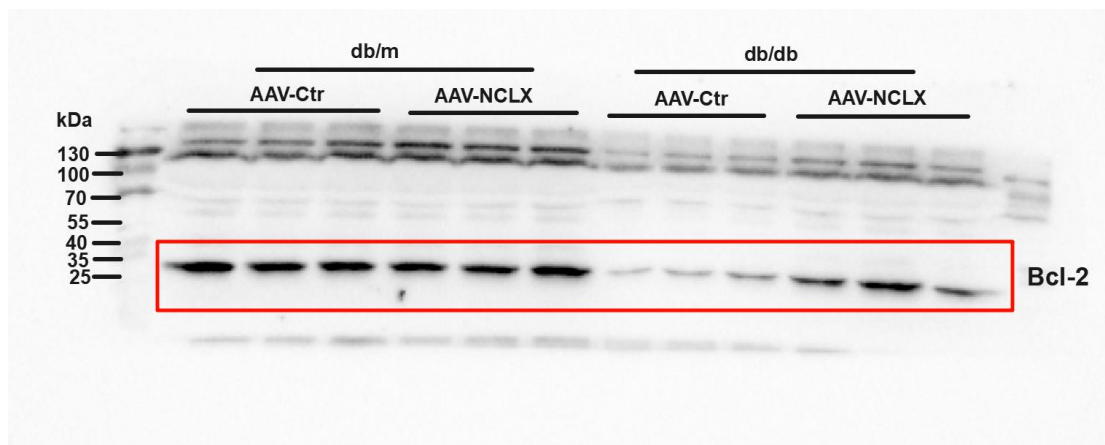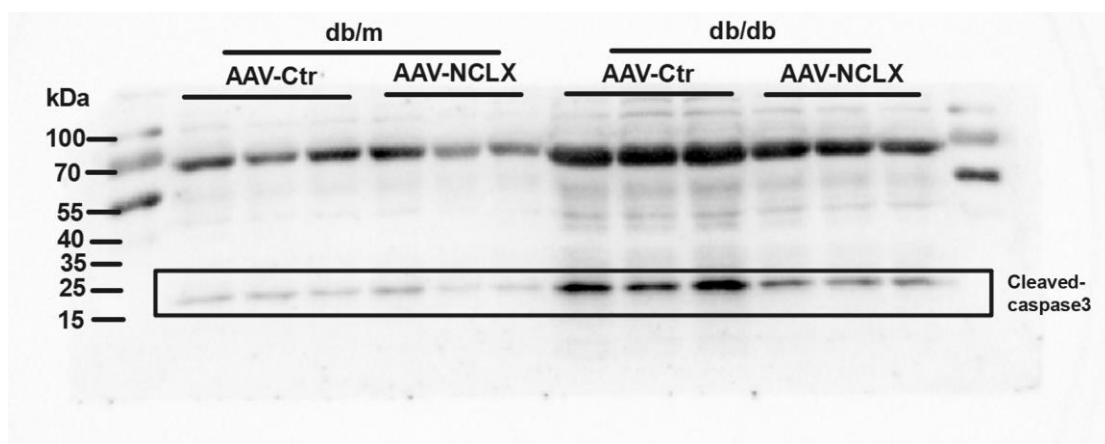

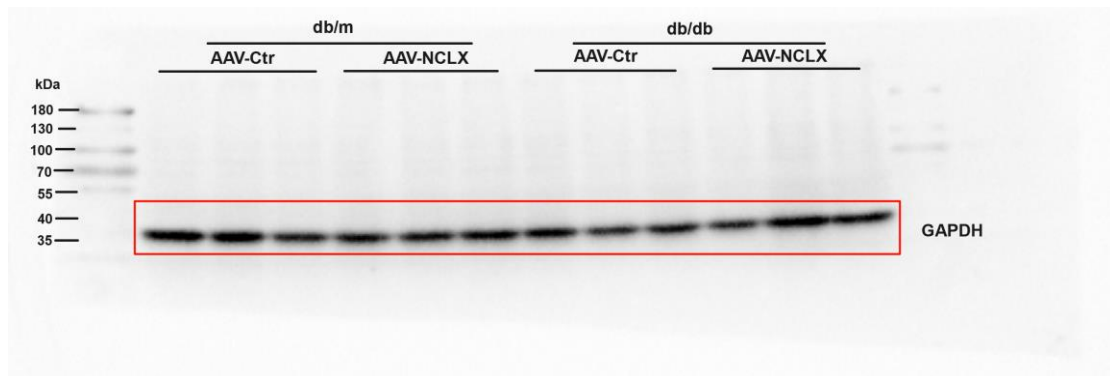

Supplement: Supplementary file 7 — Source data Fig. 5 [file 44318_2024_263_MOESM7_ESM.zip › Figure 5/5E/5E Western blot description for cropped image.pdf]

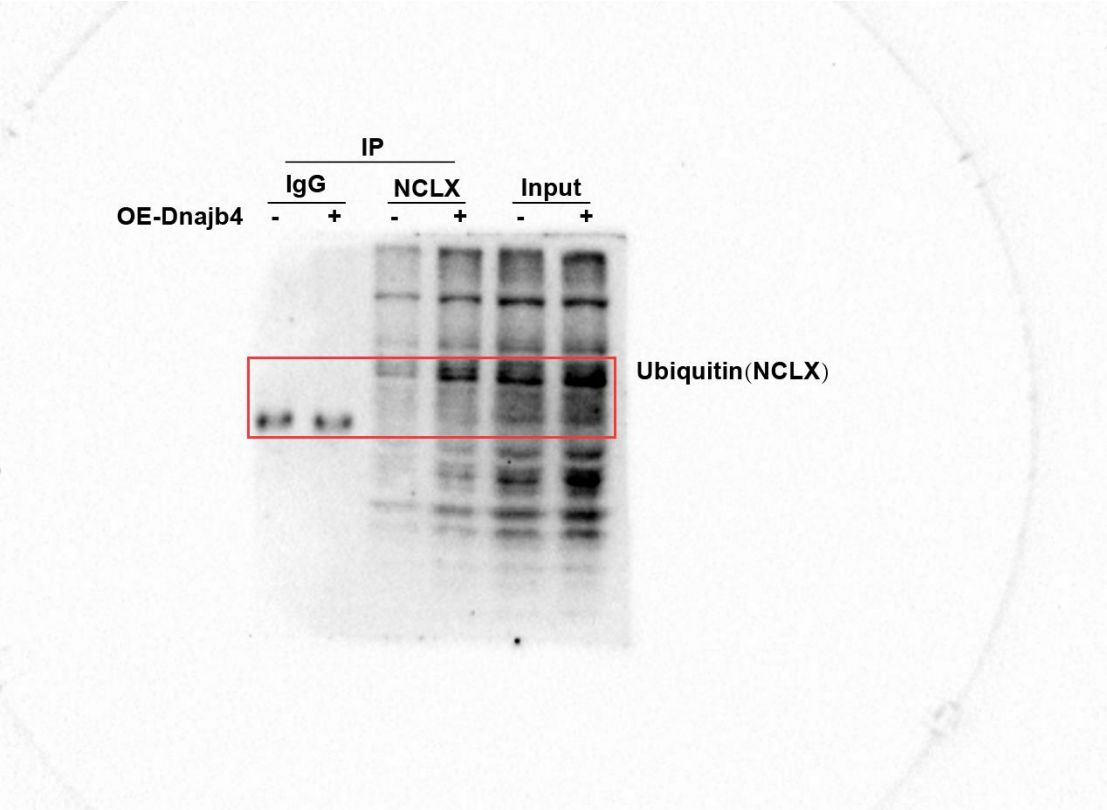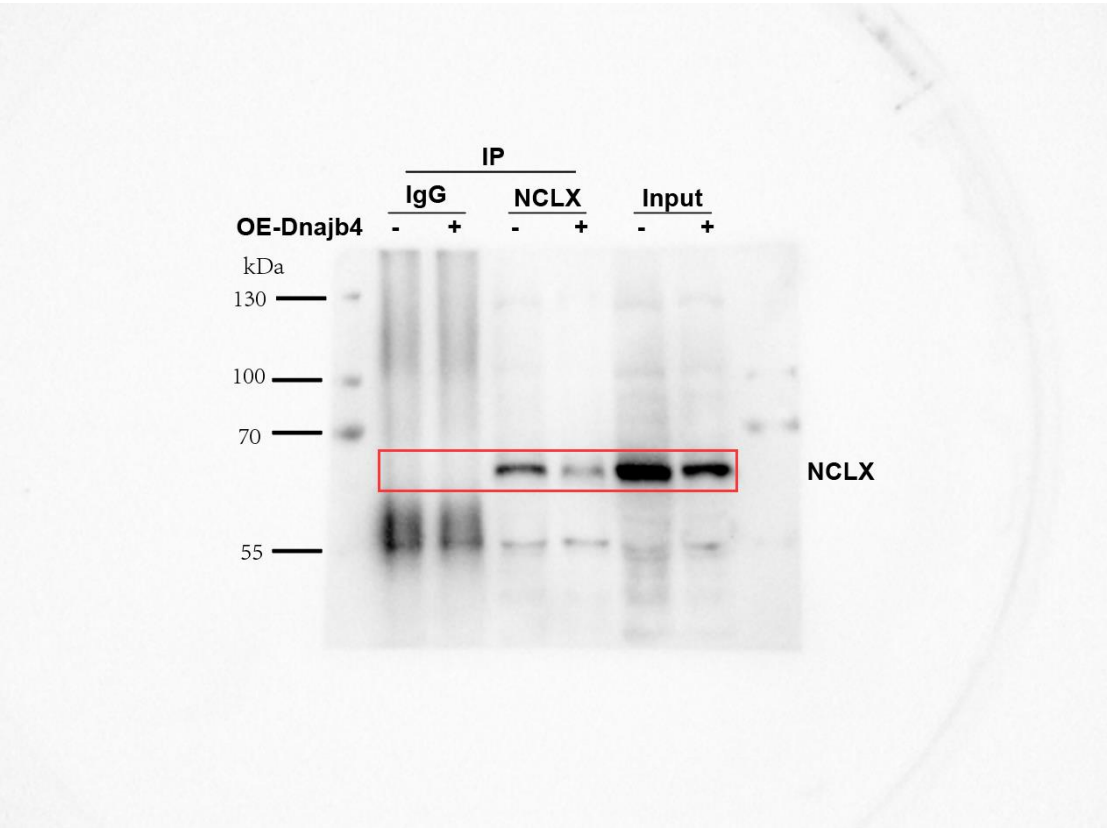

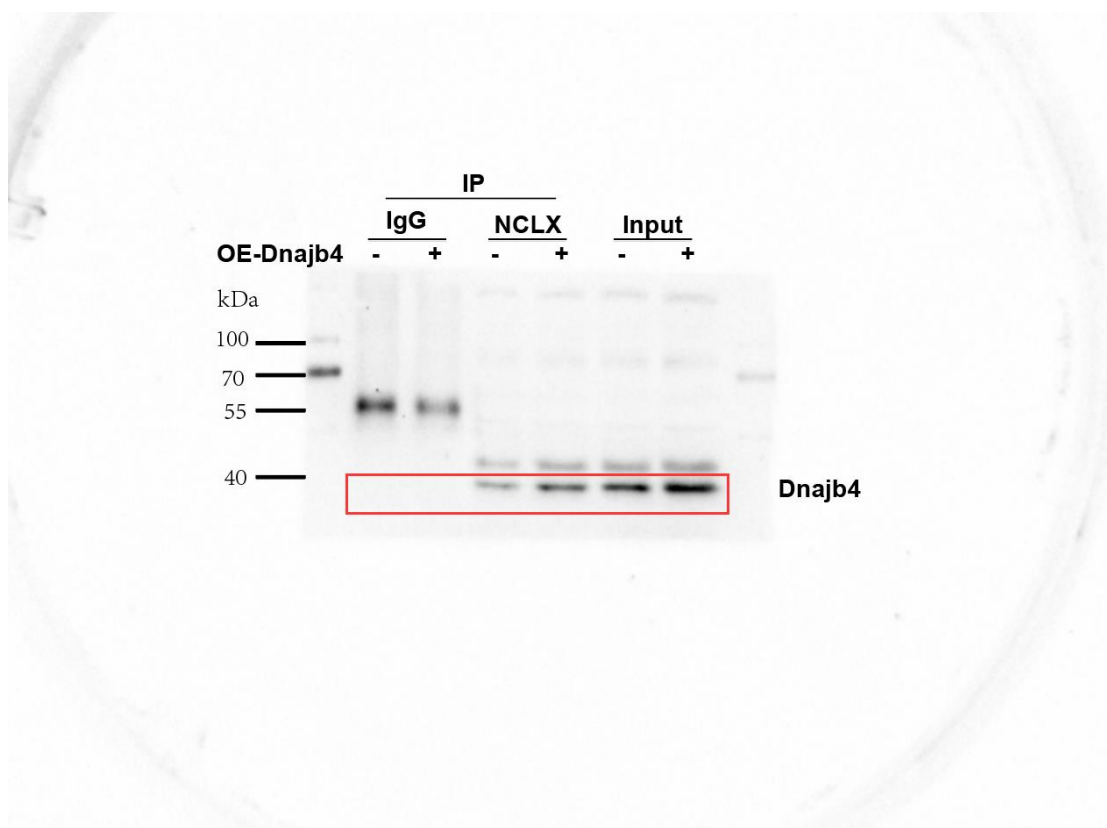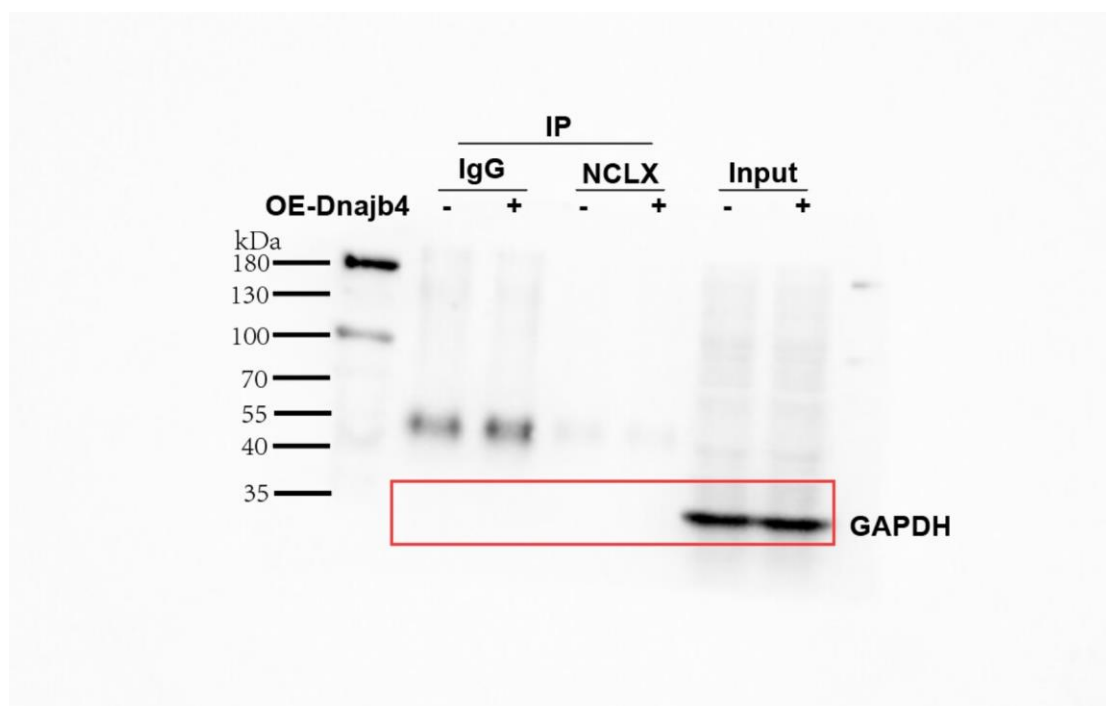

Supplement: Supplementary file 7 — Source data Fig. 5 [file 44318_2024_263_MOESM7_ESM.zip › Figure 5/5K/5K Western blot description for cropped image.pdf]

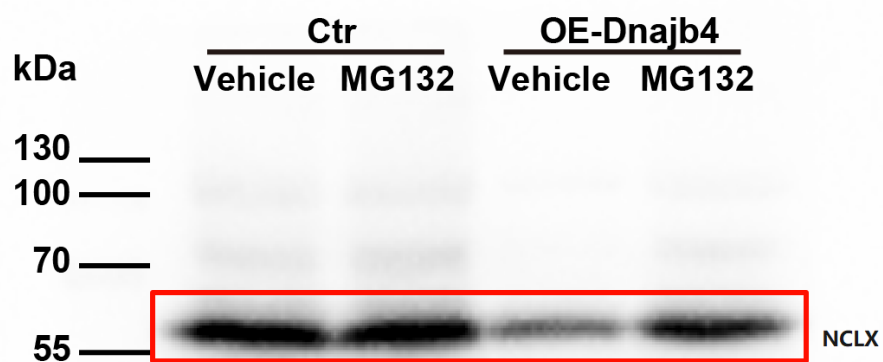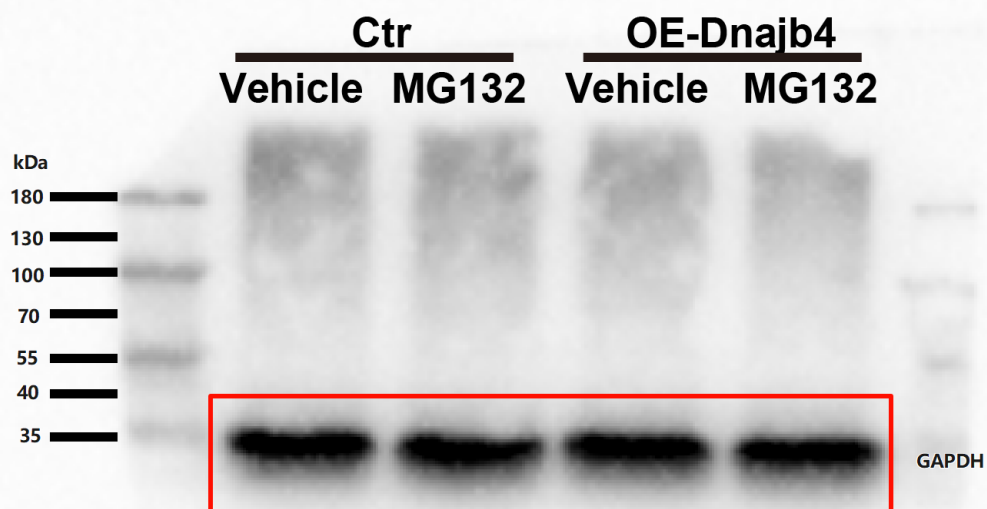

Supplement: Supplementary file 7 — Source data Fig. 5 [file 44318_2024_263_MOESM7_ESM.zip › Figure 5/5L/5L Western blot description for cropped image.pdf]

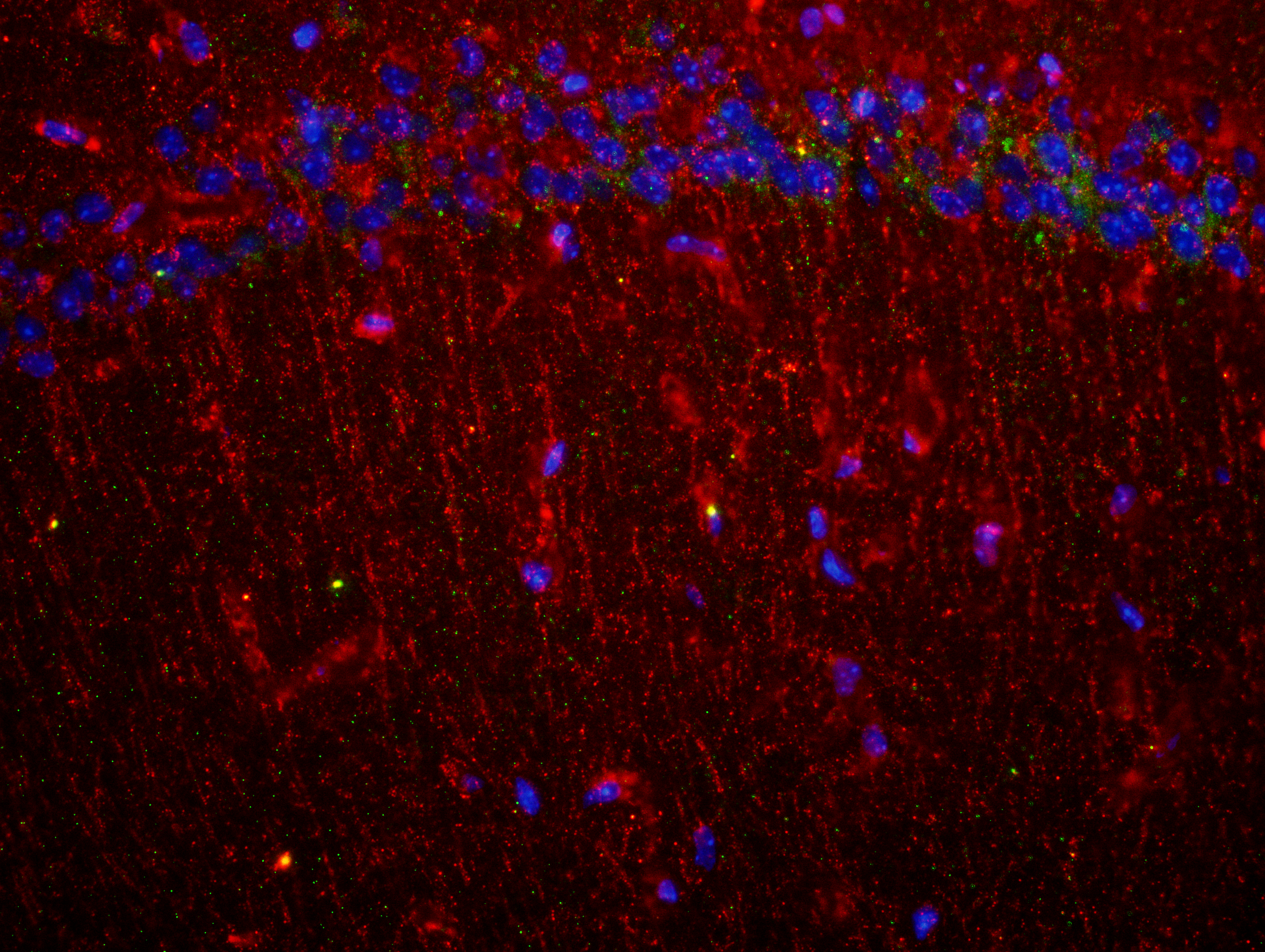

Supplement: Supplementary file 7 — Source data Fig. 5 [file 44318_2024_263_MOESM7_ESM.zip › Figure 5/5B/Fig 5B Immunofluorescence image/dbdb;AAV-Ctr/dbdb;AAV-Ctr Merge.tif]

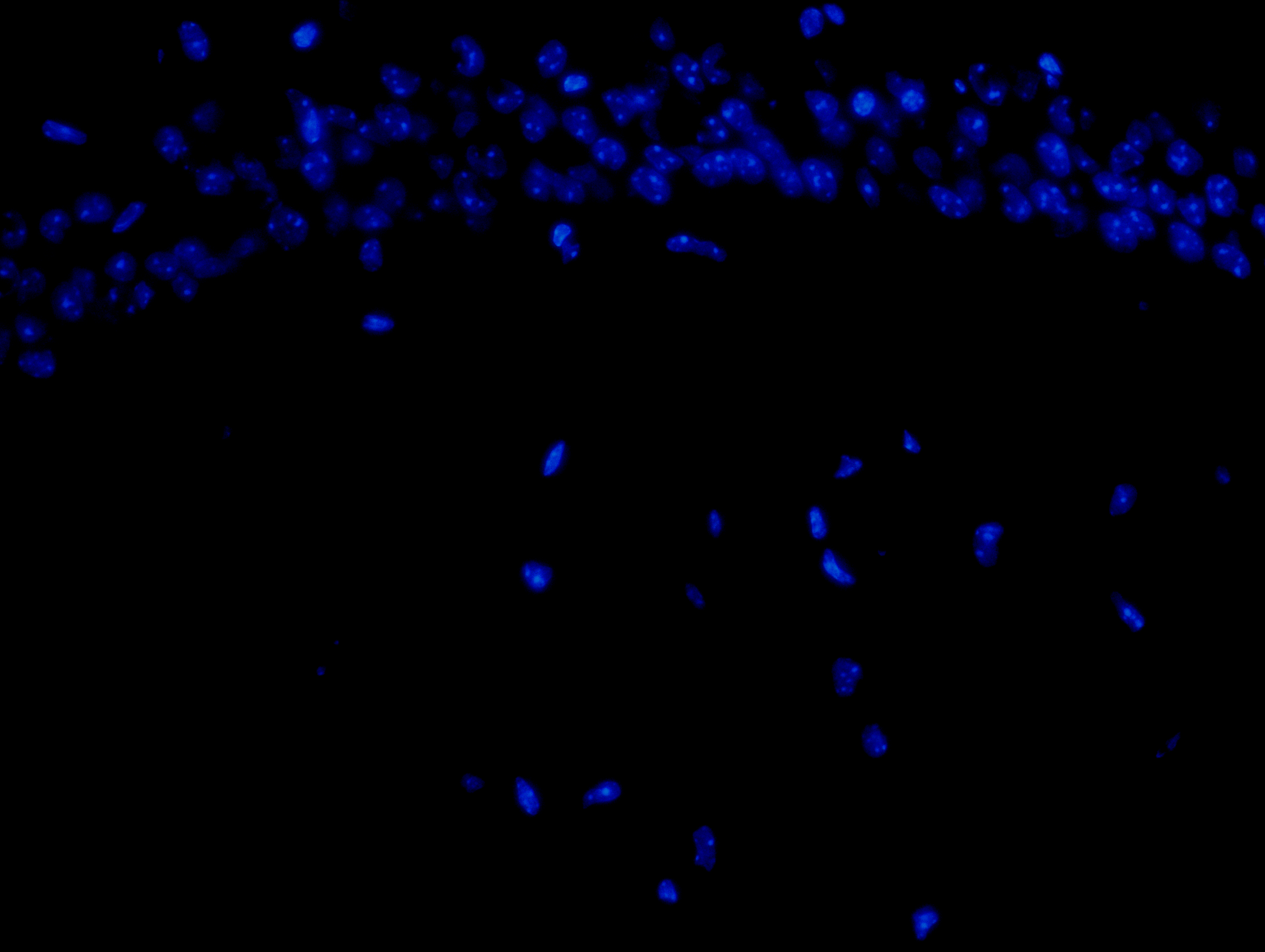

Supplement: Supplementary file 7 — Source data Fig. 5 [file 44318_2024_263_MOESM7_ESM.zip › Figure 5/5B/Fig 5B Immunofluorescence image/dbdb;AAV-Ctr/dbdb;AAV-Ctr DAPI.tif]

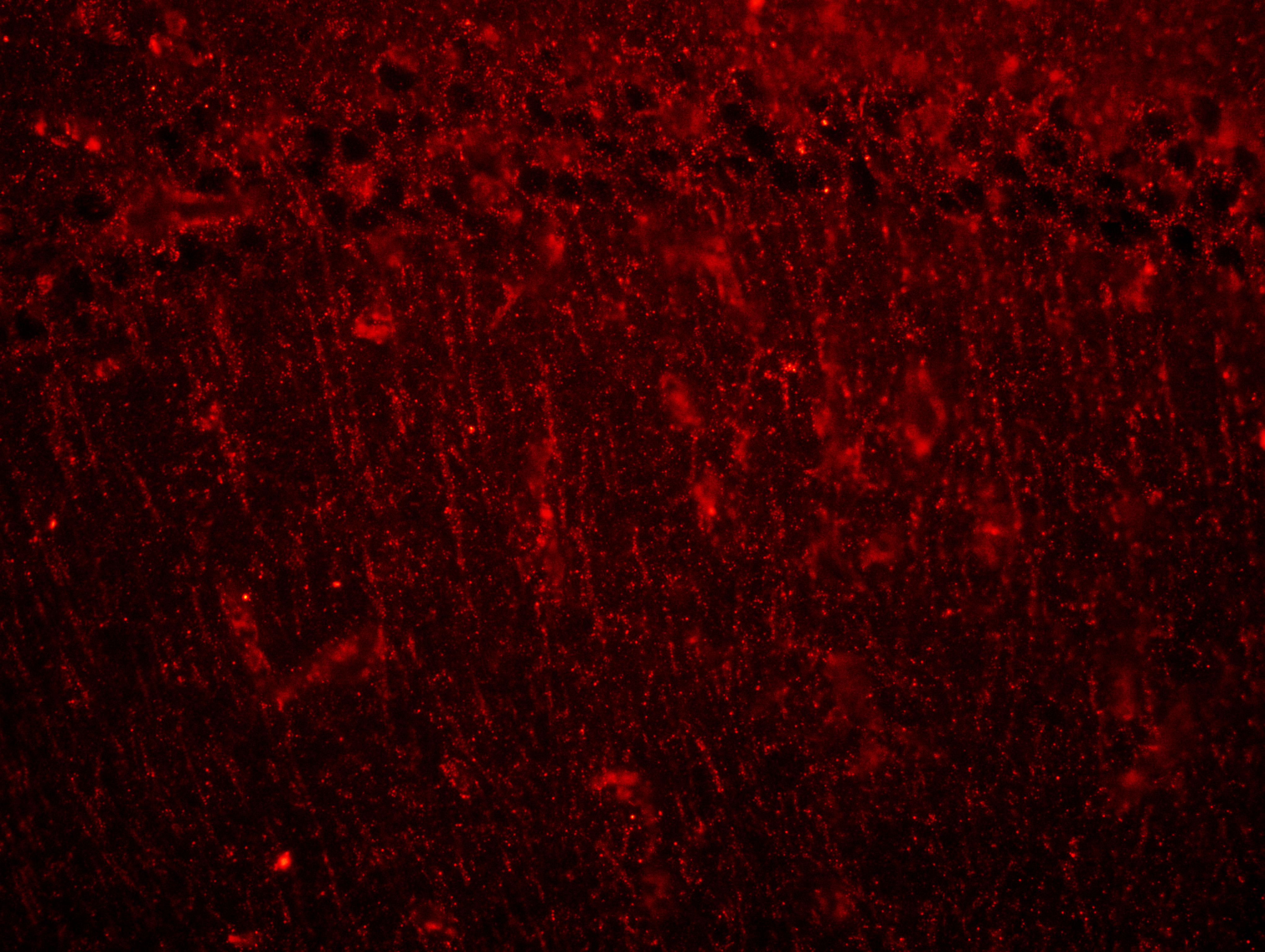

Supplement: Supplementary file 7 — Source data Fig. 5 [file 44318_2024_263_MOESM7_ESM.zip › Figure 5/5B/Fig 5B Immunofluorescence image/dbdb;AAV-Ctr/dbdb;AAV-Ctr Map2.tif]

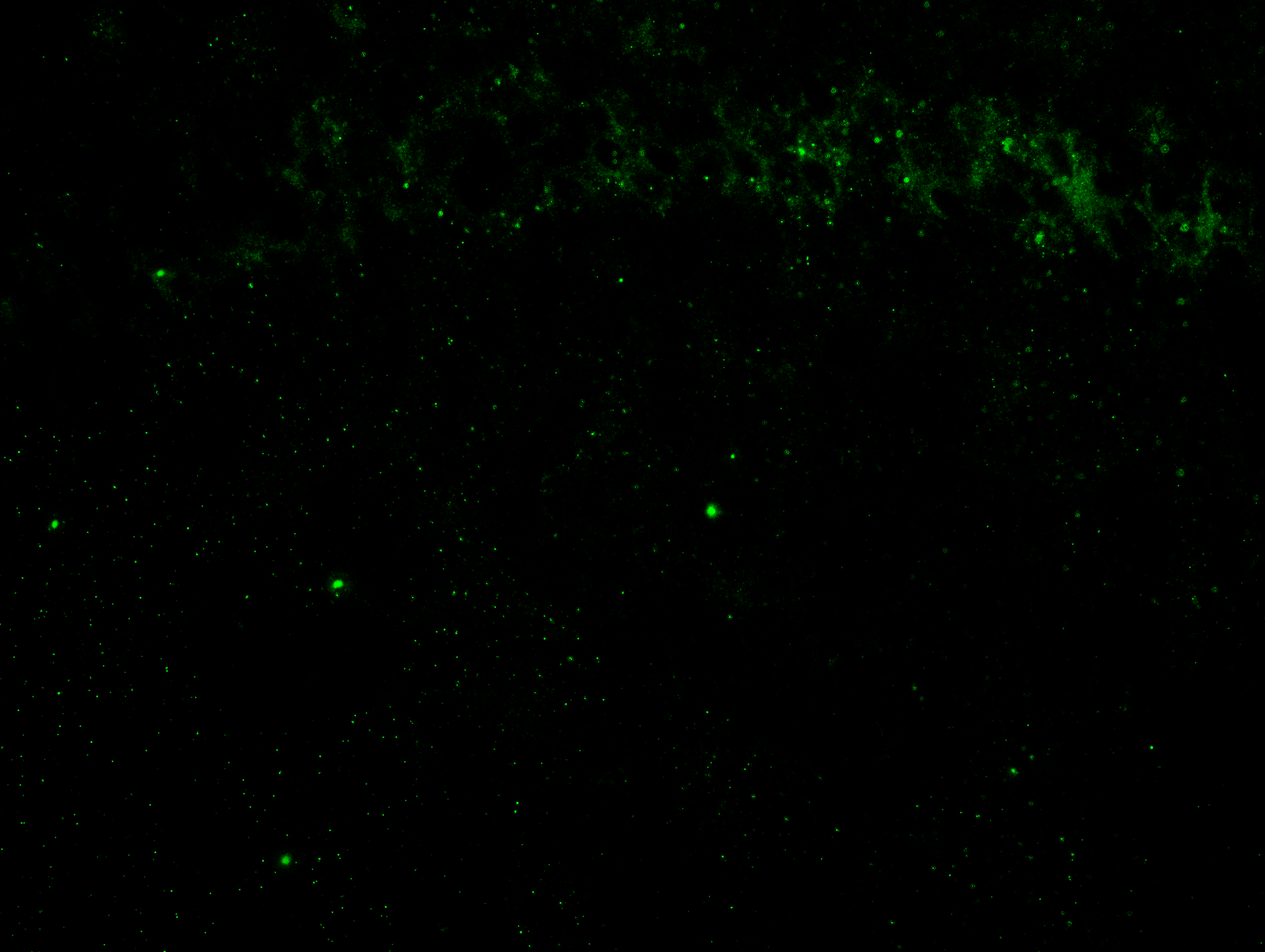

Supplement: Supplementary file 7 — Source data Fig. 5 [file 44318_2024_263_MOESM7_ESM.zip › Figure 5/5B/Fig 5B Immunofluorescence image/dbdb;AAV-Ctr/dbdb;AAV-Ctr NCLX.tif]

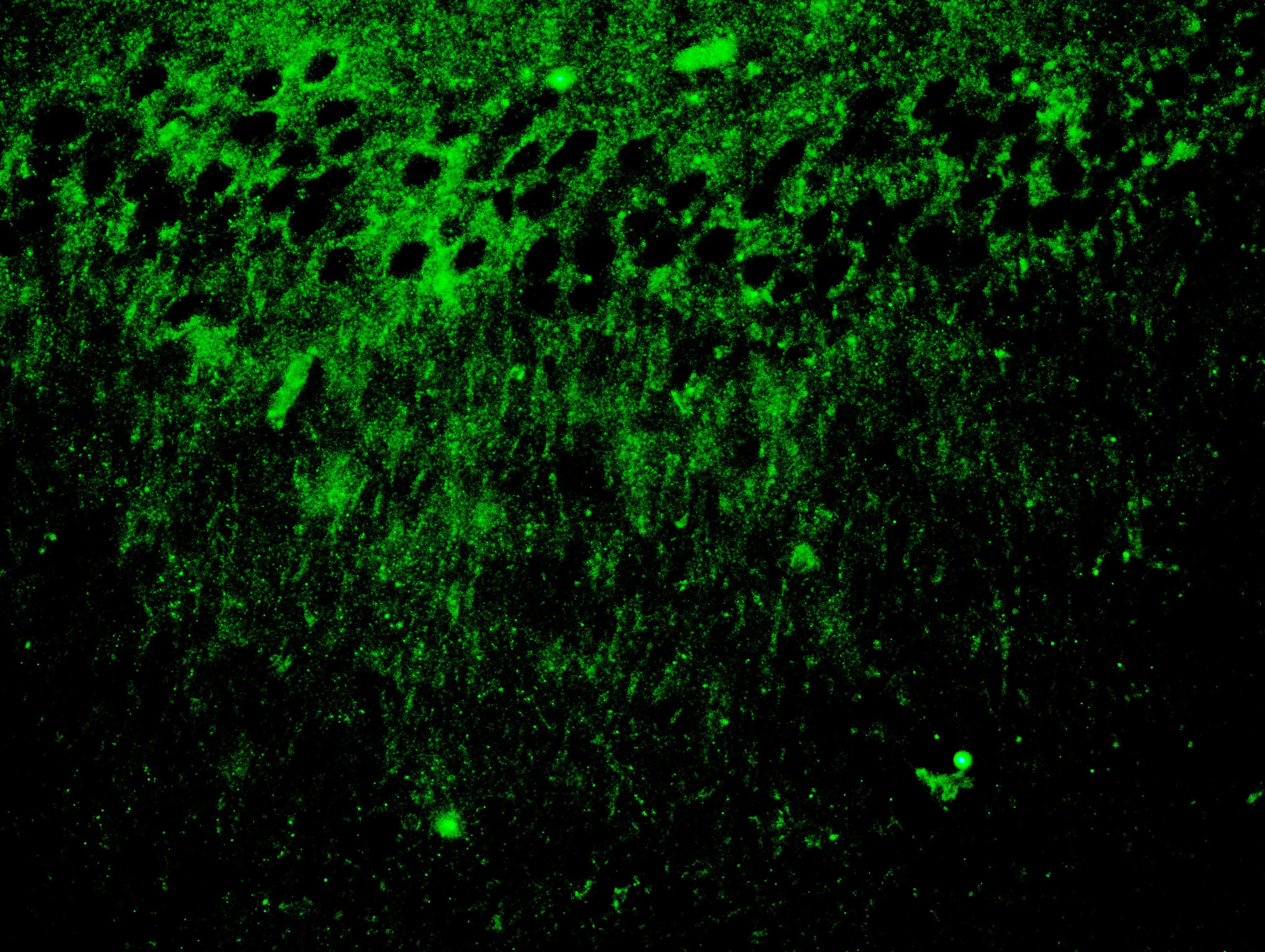

Supplement: Supplementary file 7 — Source data Fig. 5 [file 44318_2024_263_MOESM7_ESM.zip › Figure 5/5B/Fig 5B Immunofluorescence image/dbm;AAV-NCLX/dbm;AAV-NCLX NCLX.tif]

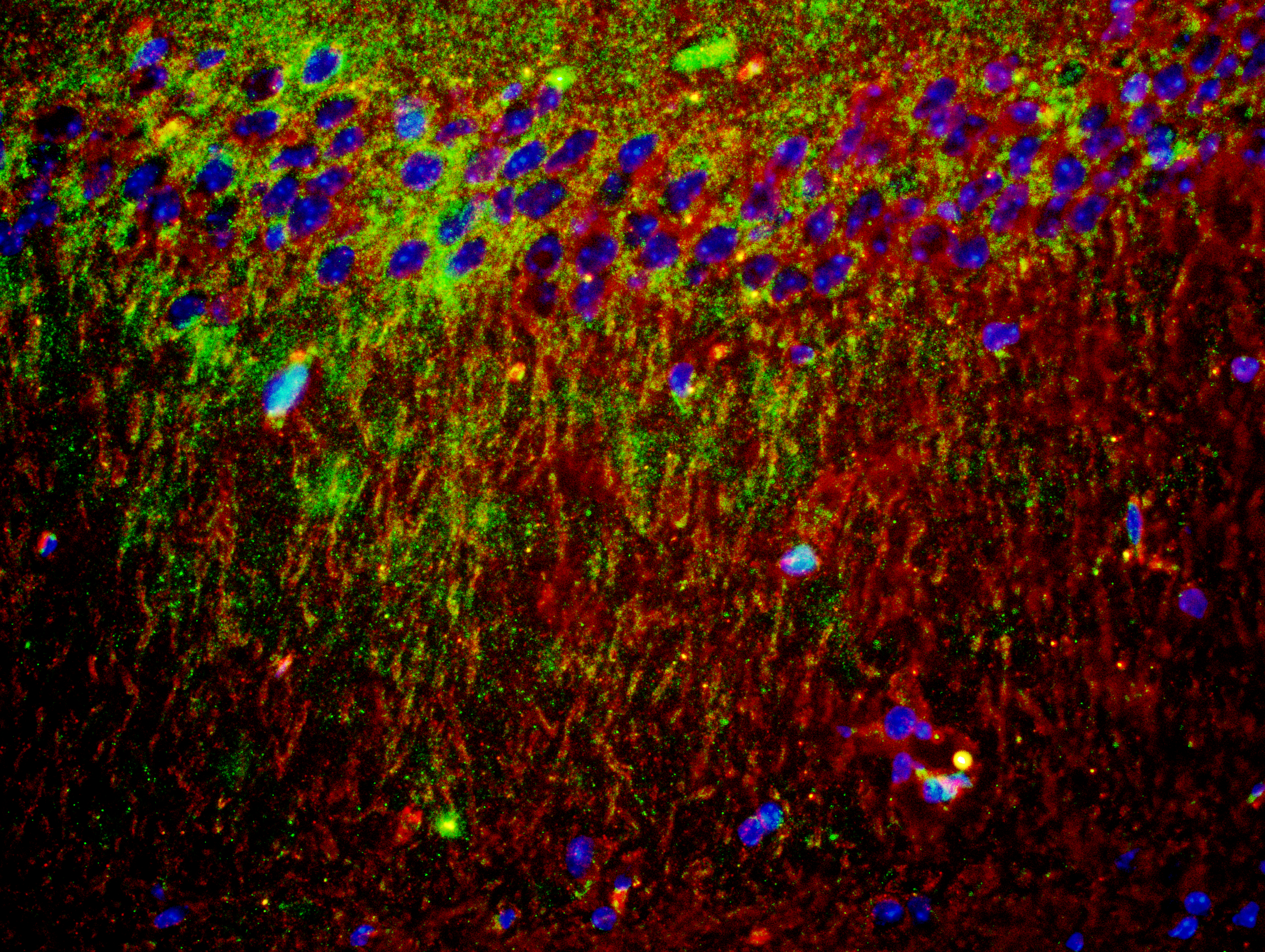

Supplement: Supplementary file 7 — Source data Fig. 5 [file 44318_2024_263_MOESM7_ESM.zip › Figure 5/5B/Fig 5B Immunofluorescence image/dbm;AAV-NCLX/dbm;AAV-NCLX Merge.tif]

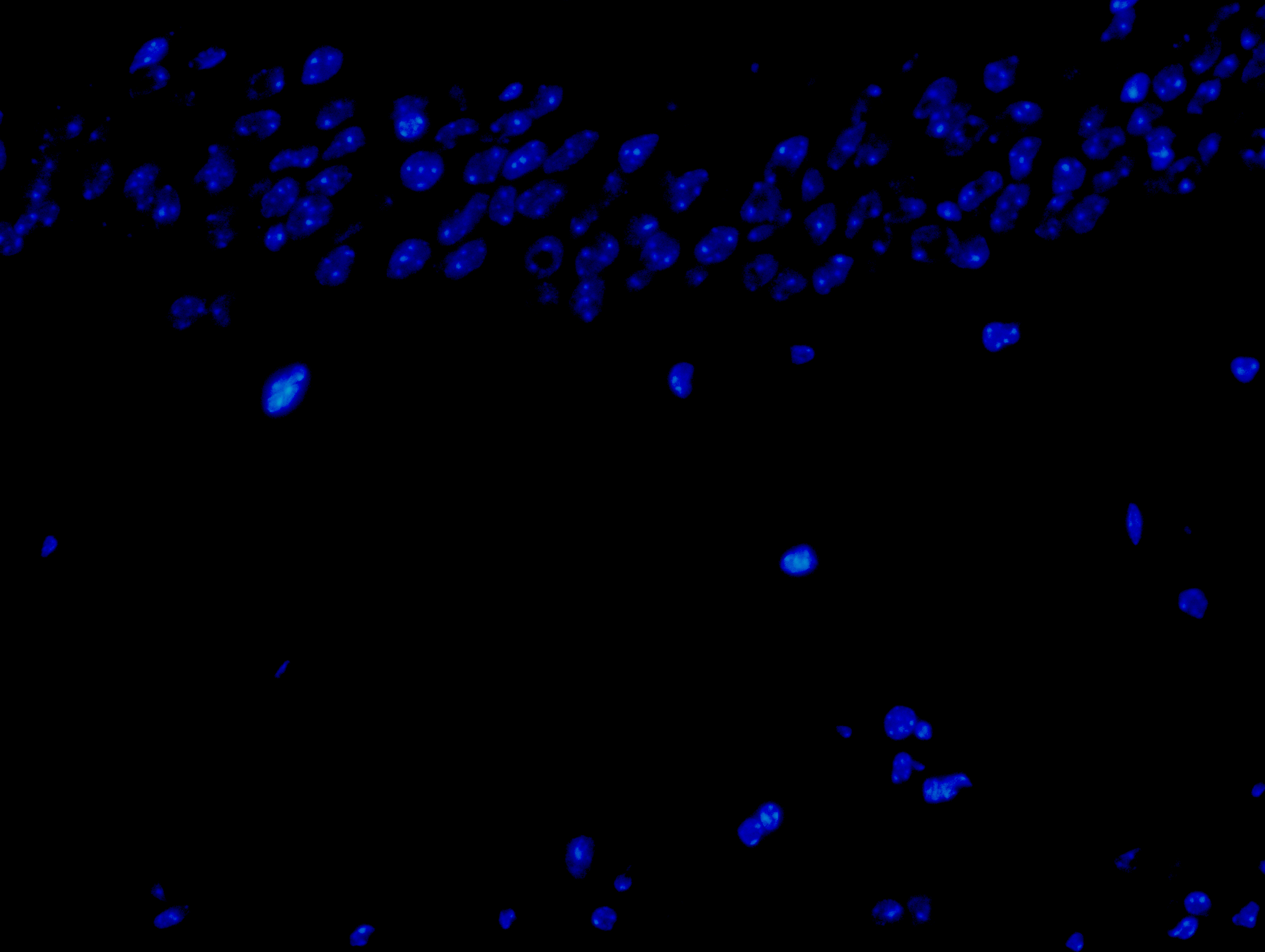

Supplement: Supplementary file 7 — Source data Fig. 5 [file 44318_2024_263_MOESM7_ESM.zip › Figure 5/5B/Fig 5B Immunofluorescence image/dbm;AAV-NCLX/dbm;AAV-NCLX DAPI.tif]

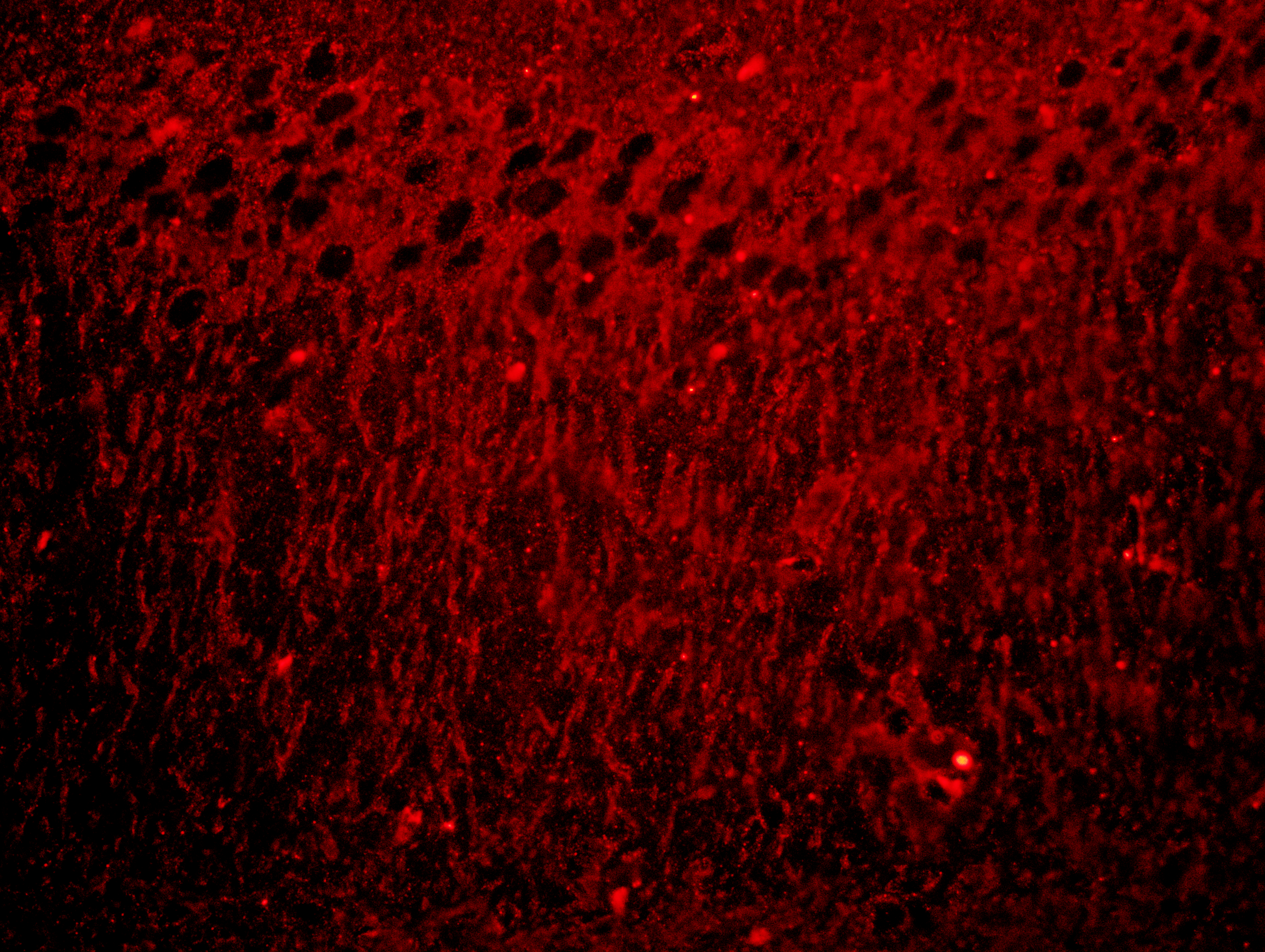

Supplement: Supplementary file 7 — Source data Fig. 5 [file 44318_2024_263_MOESM7_ESM.zip › Figure 5/5B/Fig 5B Immunofluorescence image/dbm;AAV-NCLX/dbm;AAV-NCLX Map2.tif]

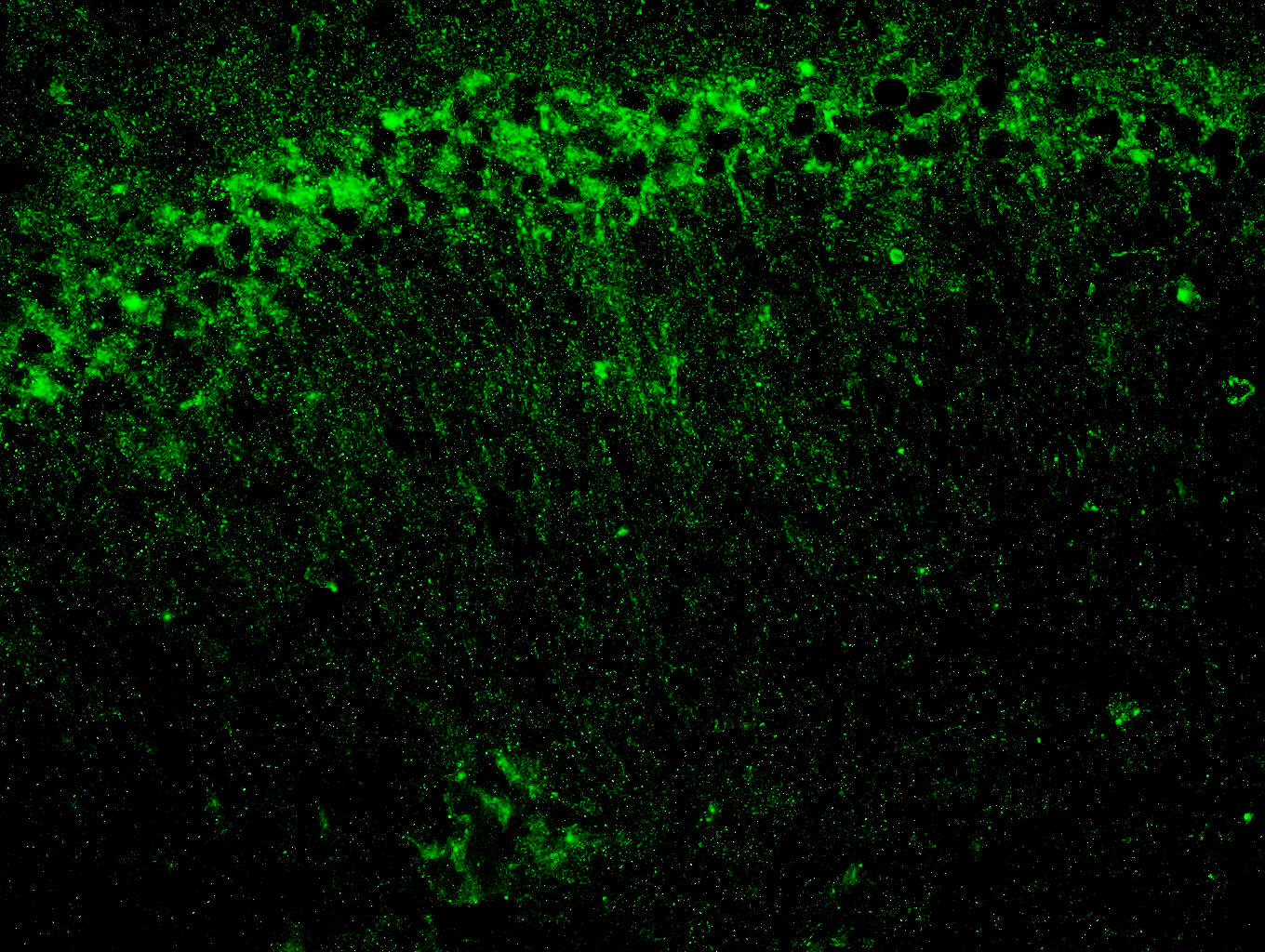

Supplement: Supplementary file 7 — Source data Fig. 5 [file 44318_2024_263_MOESM7_ESM.zip › Figure 5/5B/Fig 5B Immunofluorescence image/dbm;AAV-Ctr/dbm;AAV-Ctr NCLX.tif]

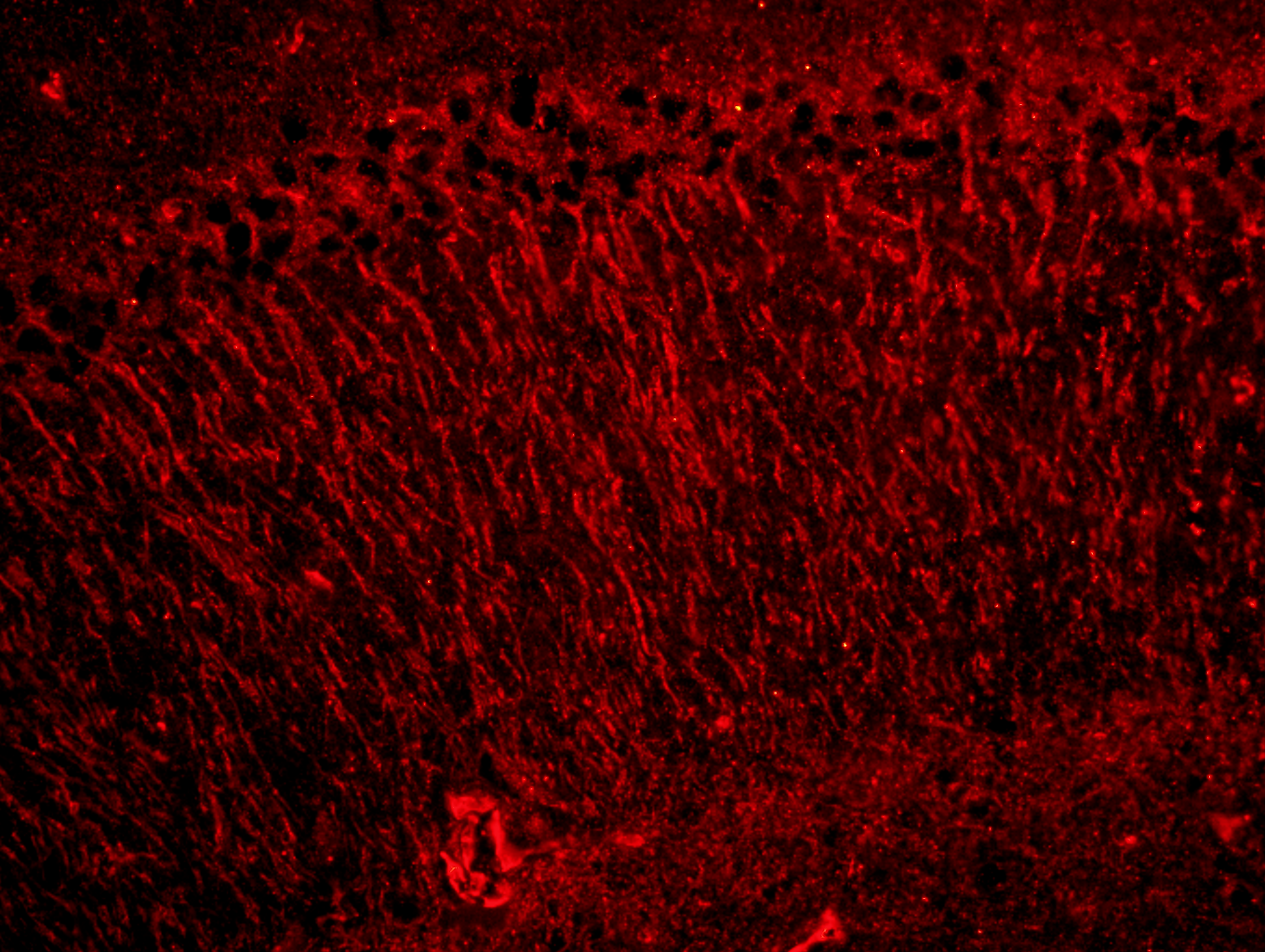

Supplement: Supplementary file 7 — Source data Fig. 5 [file 44318_2024_263_MOESM7_ESM.zip › Figure 5/5B/Fig 5B Immunofluorescence image/dbm;AAV-Ctr/dbm;AAV-Ctr Map2.tif]

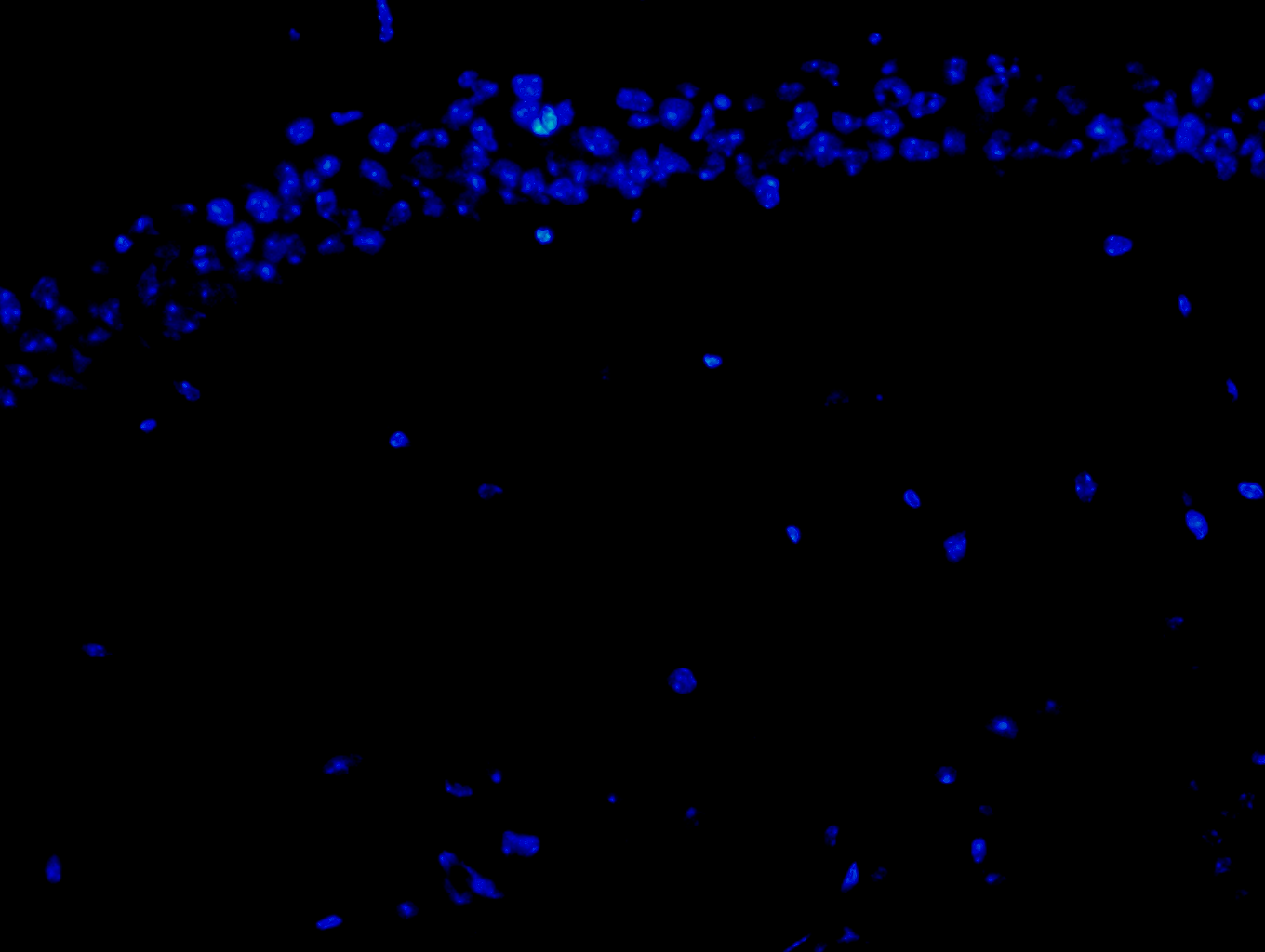

Supplement: Supplementary file 7 — Source data Fig. 5 [file 44318_2024_263_MOESM7_ESM.zip › Figure 5/5B/Fig 5B Immunofluorescence image/dbm;AAV-Ctr/dbm;AAV-Ctr DAPI.tif]

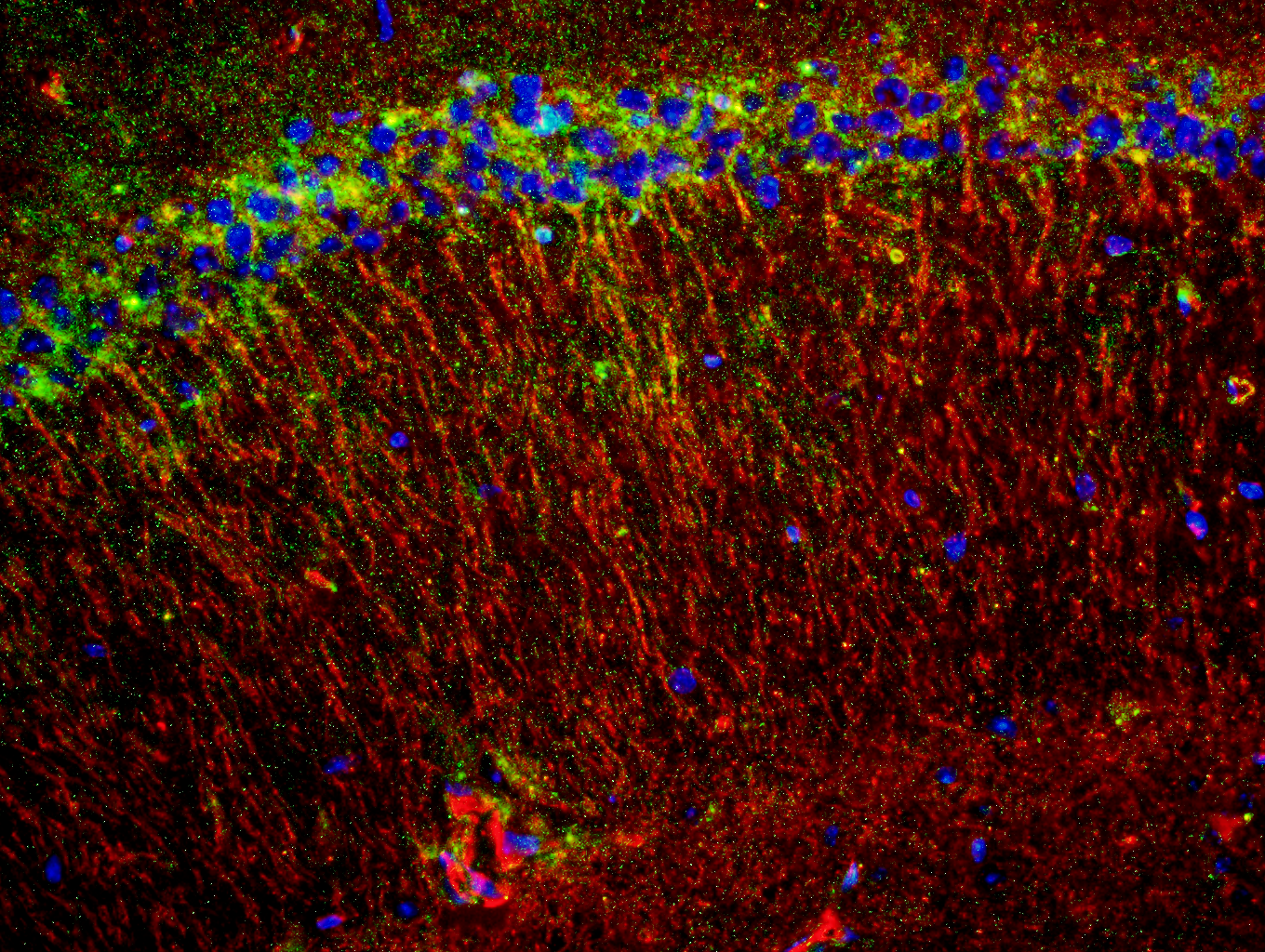

Supplement: Supplementary file 7 — Source data Fig. 5 [file 44318_2024_263_MOESM7_ESM.zip › Figure 5/5B/Fig 5B Immunofluorescence image/dbm;AAV-Ctr/dbm;AAV-Ctr Merge.tif]

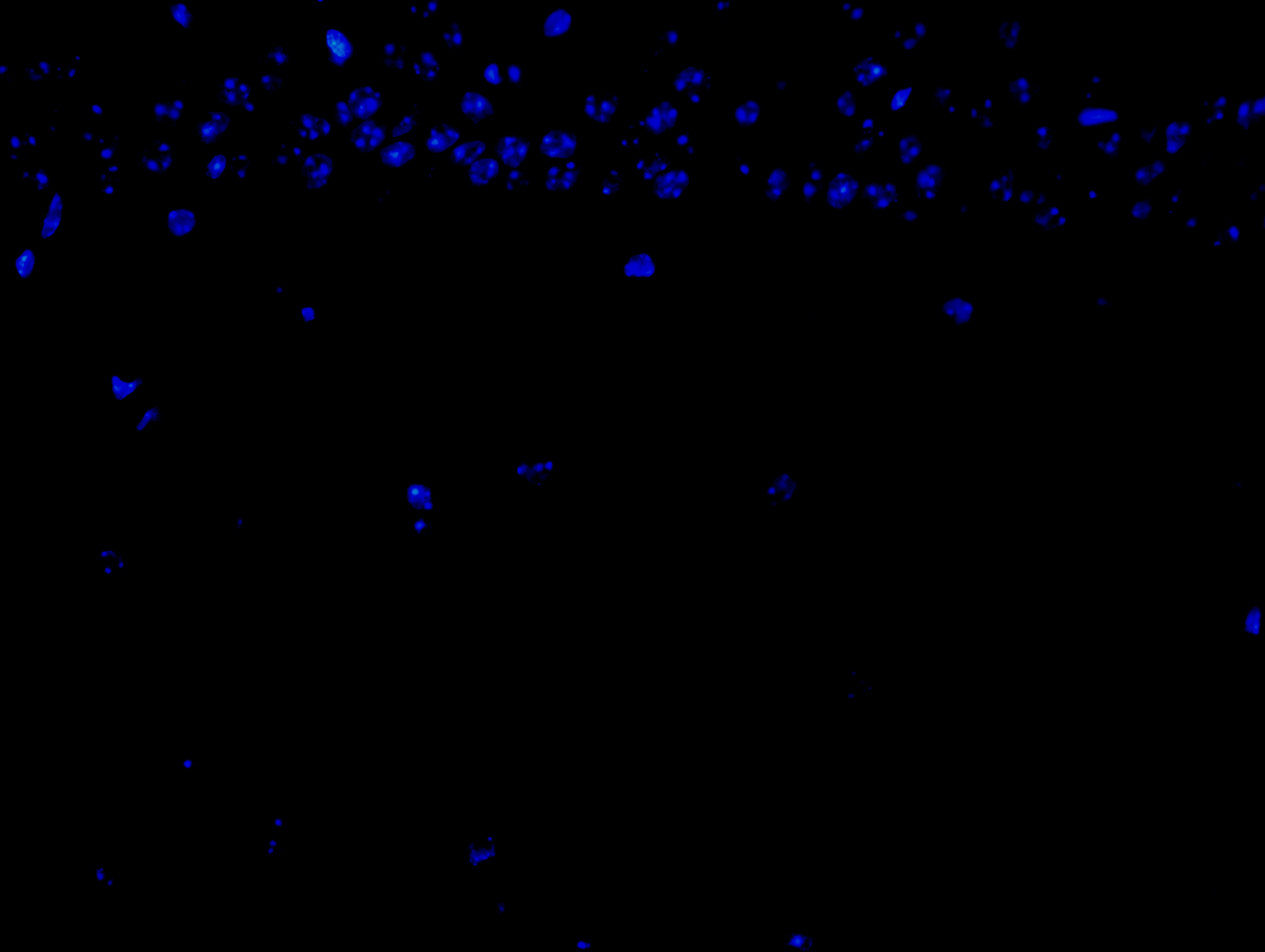

Supplement: Supplementary file 7 — Source data Fig. 5 [file 44318_2024_263_MOESM7_ESM.zip › Figure 5/5B/Fig 5B Immunofluorescence image/dbdb;AAV-NCLX/dbdb;AAV-NCLX DAPI.tif]

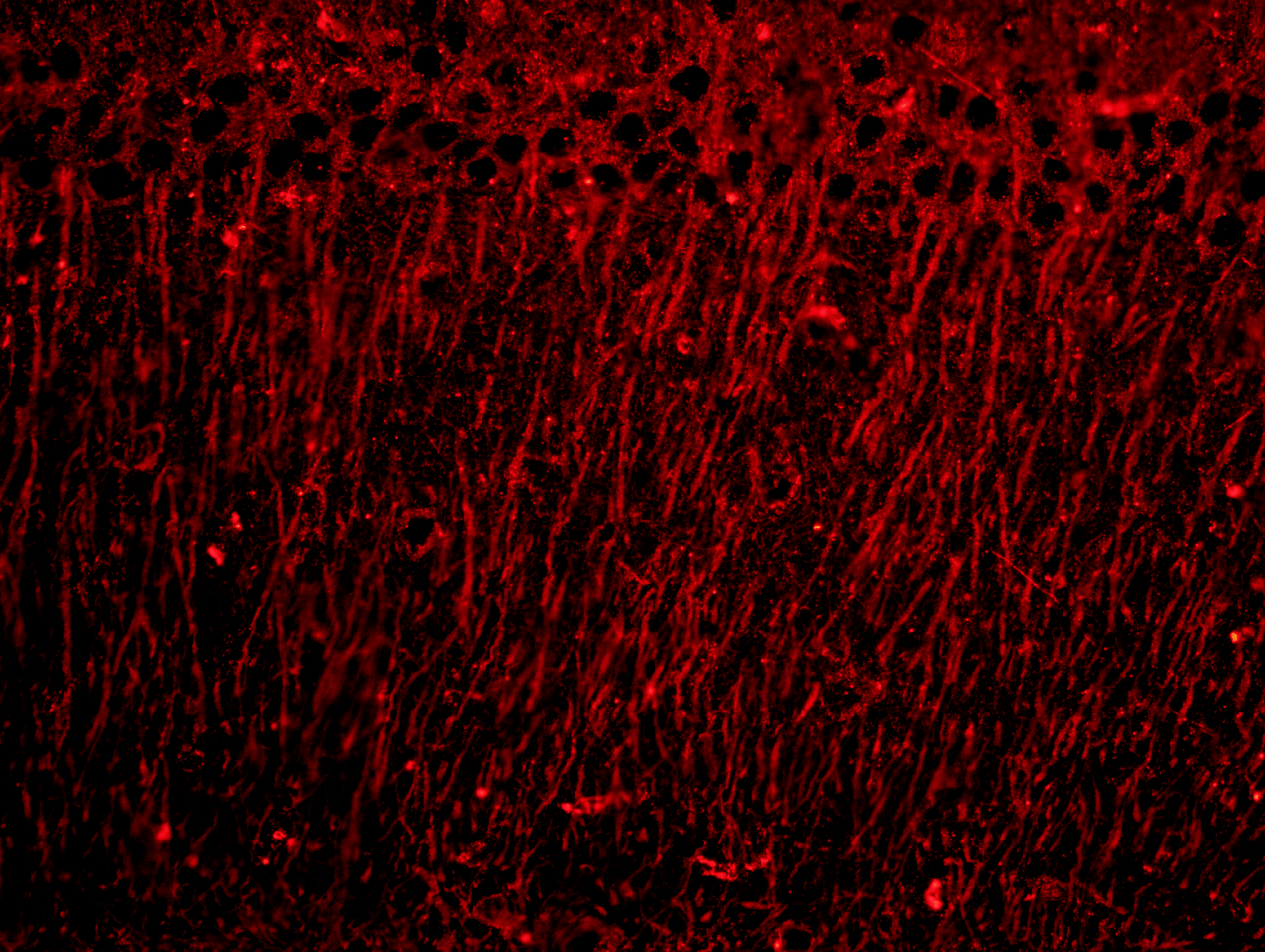

Supplement: Supplementary file 7 — Source data Fig. 5 [file 44318_2024_263_MOESM7_ESM.zip › Figure 5/5B/Fig 5B Immunofluorescence image/dbdb;AAV-NCLX/dbdb;AAV-NCLX Map2.tif]

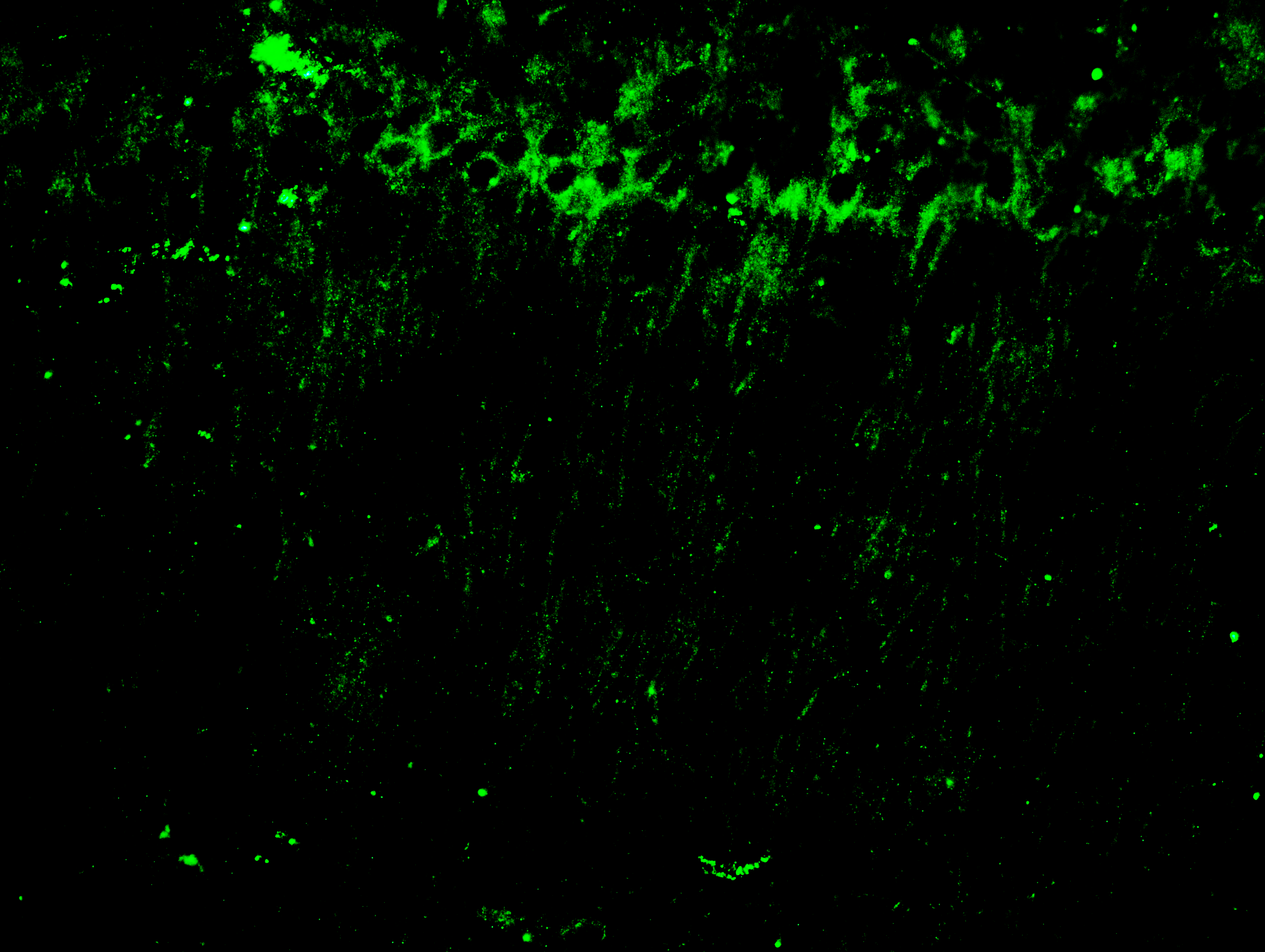

Supplement: Supplementary file 7 — Source data Fig. 5 [file 44318_2024_263_MOESM7_ESM.zip › Figure 5/5B/Fig 5B Immunofluorescence image/dbdb;AAV-NCLX/dbdb;AAV-NCLX NCLX .tif]

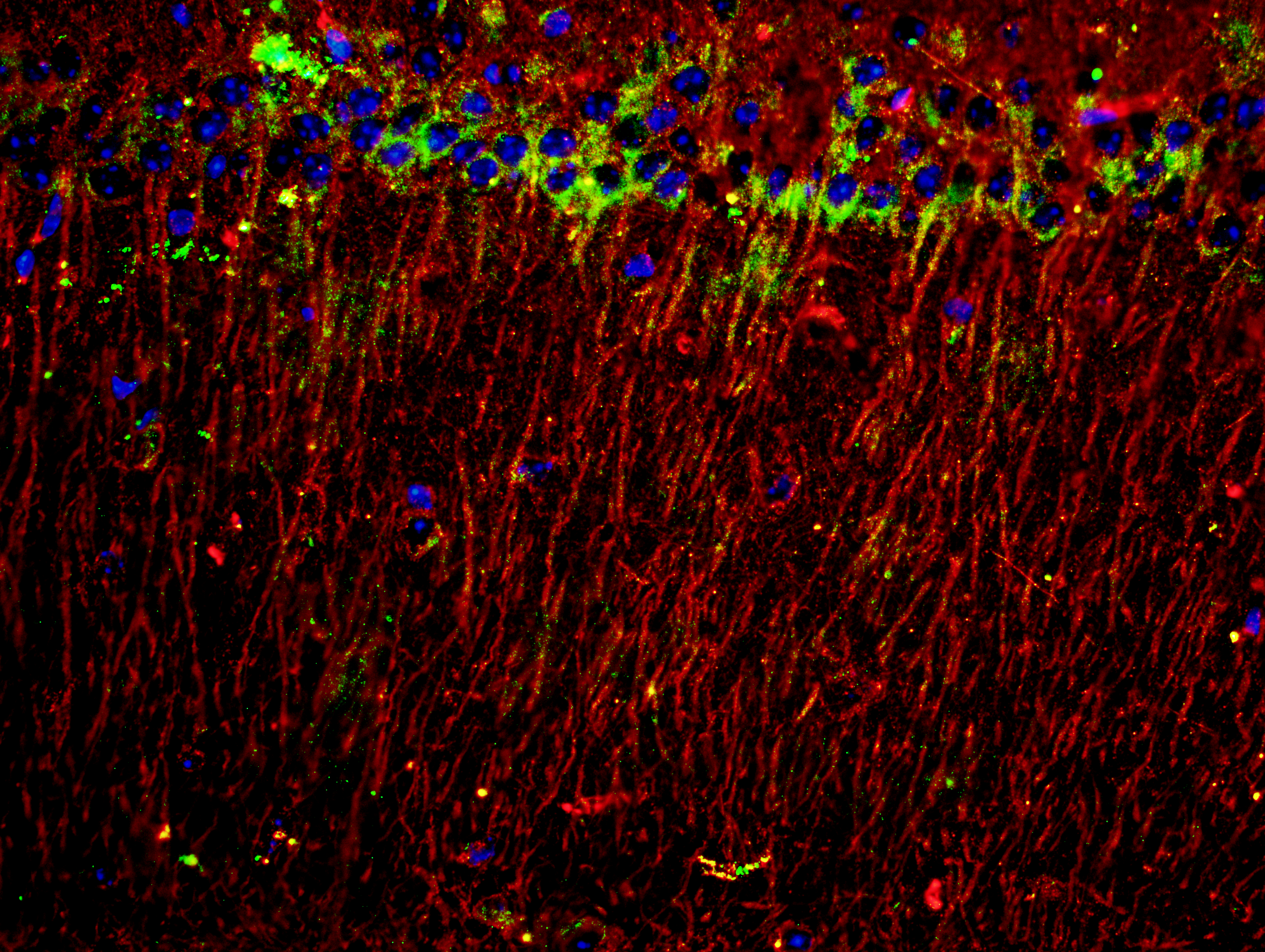

Supplement: Supplementary file 7 — Source data Fig. 5 [file 44318_2024_263_MOESM7_ESM.zip › Figure 5/5B/Fig 5B Immunofluorescence image/dbdb;AAV-NCLX/dbdb;AAV-NCLX Merge.tif]

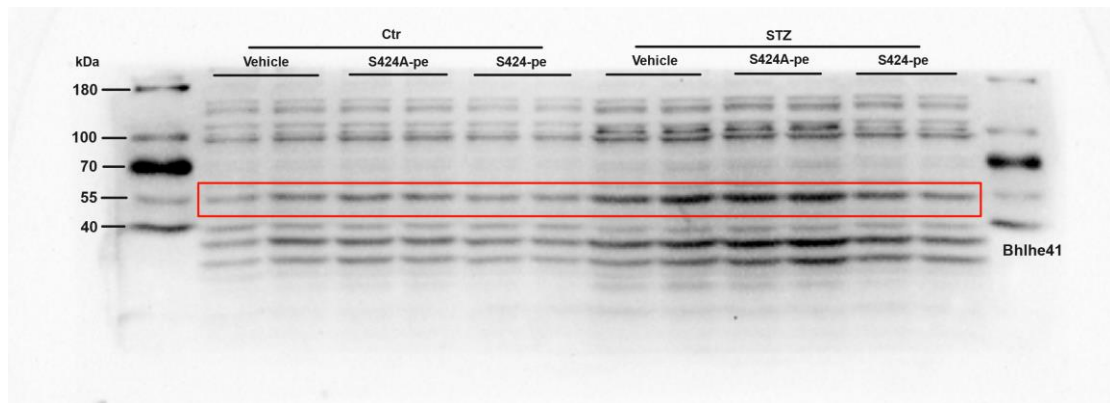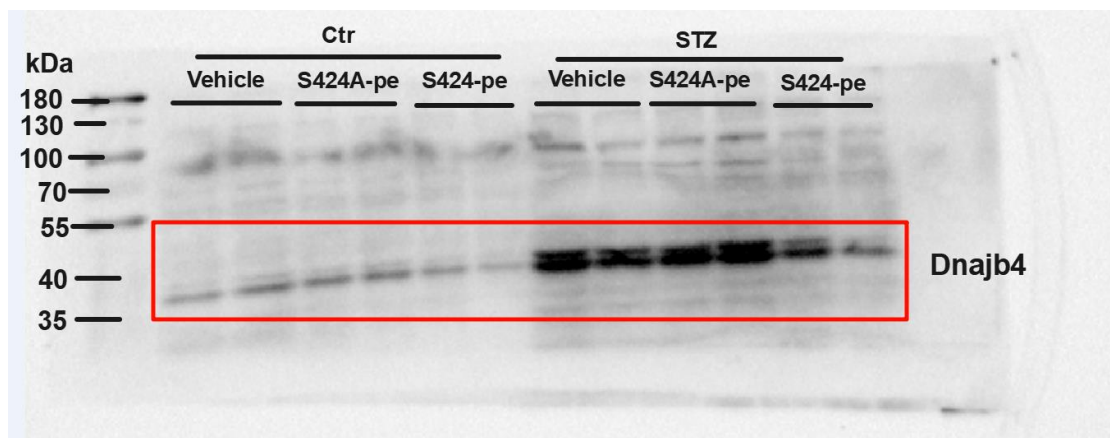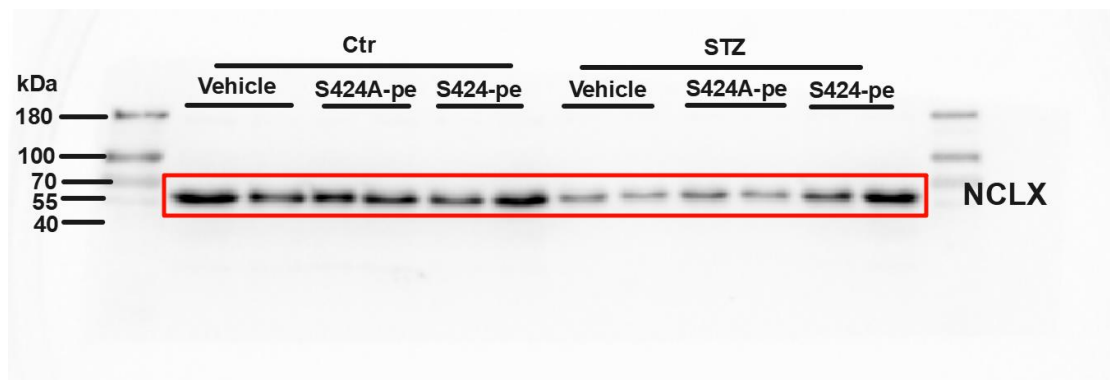

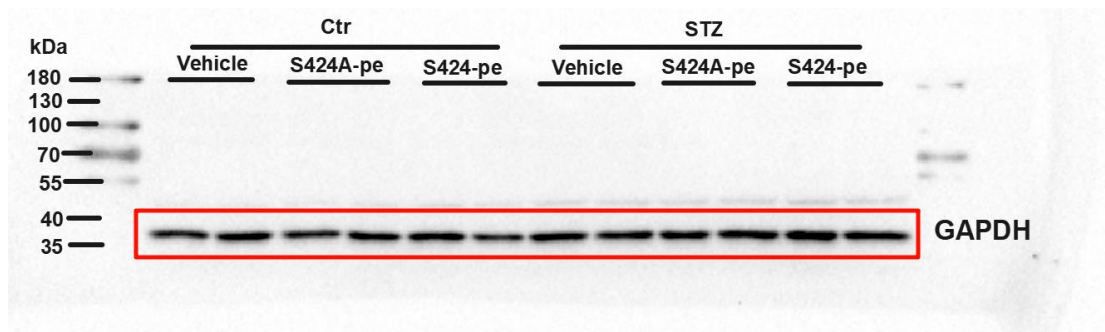

Supplement: Supplementary file 8 — Source data Fig. 6 [file 44318_2024_263_MOESM8_ESM.zip › Figure 6/6F/6F Western blot description for cropped image.pdf]

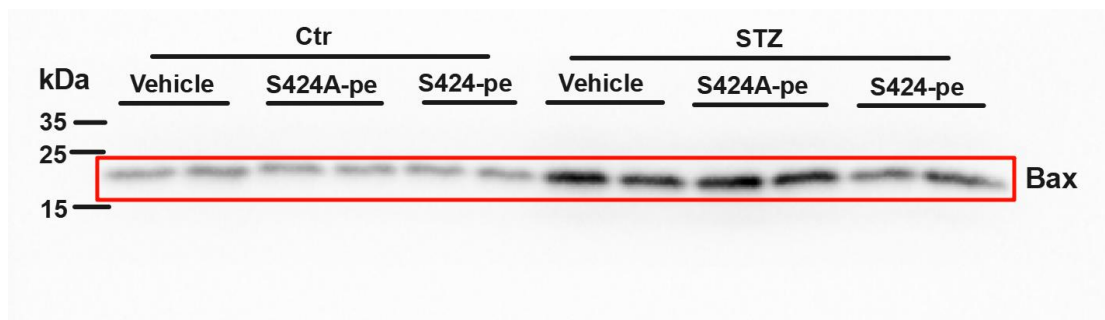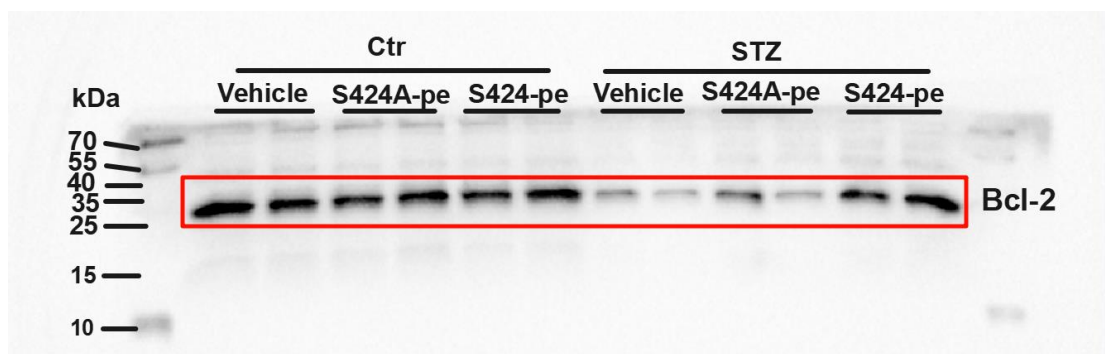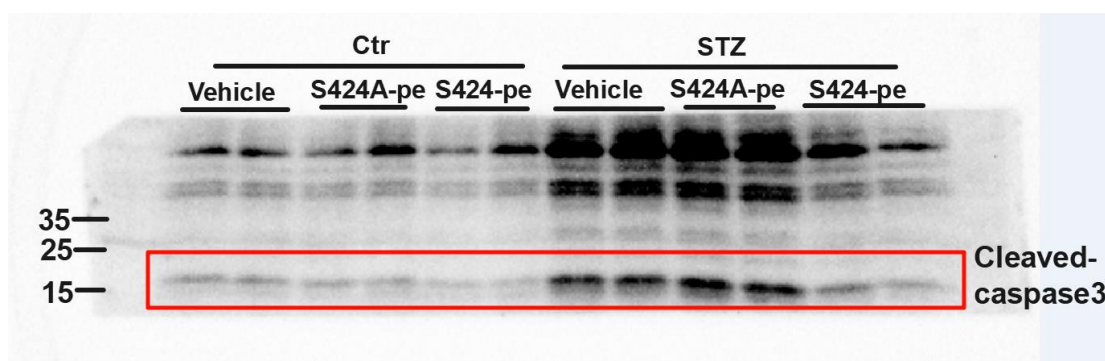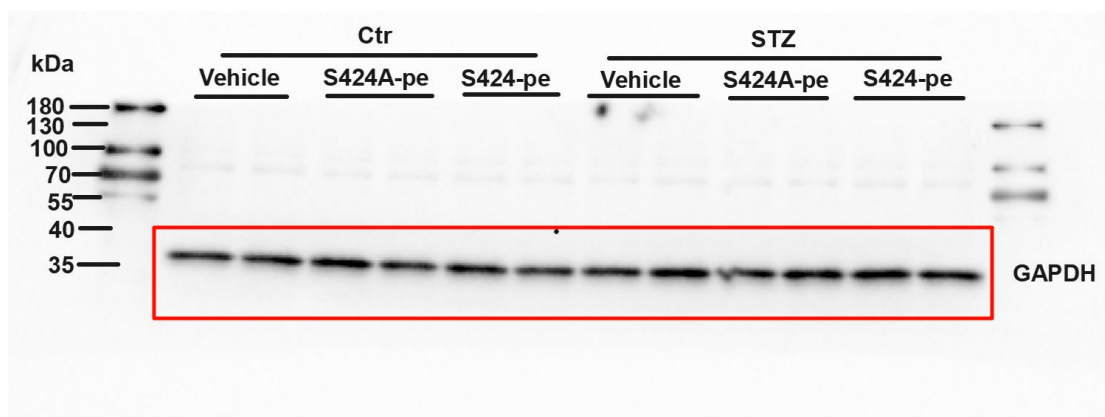

Supplement: Supplementary file 8 — Source data Fig. 6 [file 44318_2024_263_MOESM8_ESM.zip › Figure 6/6H/6H Western blot description for cropped image.pdf]

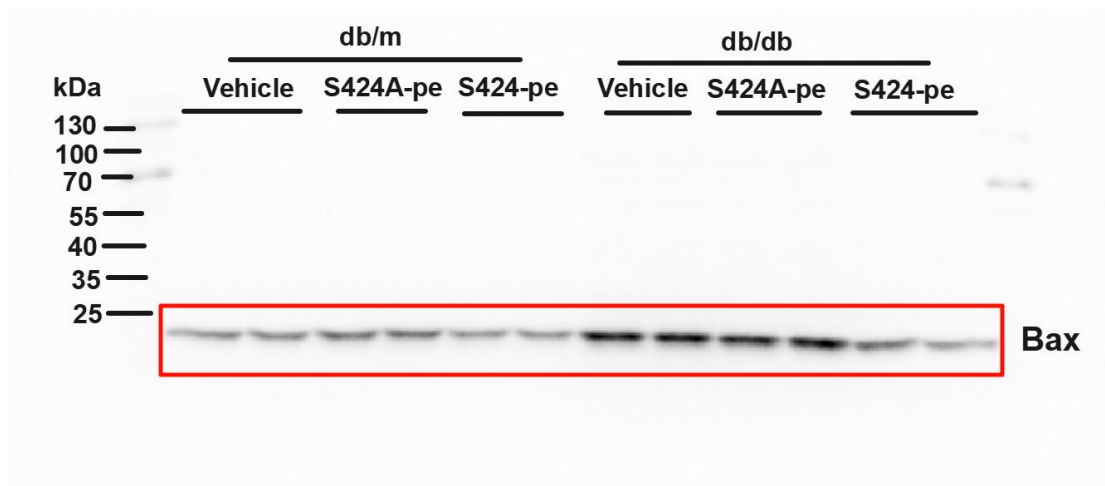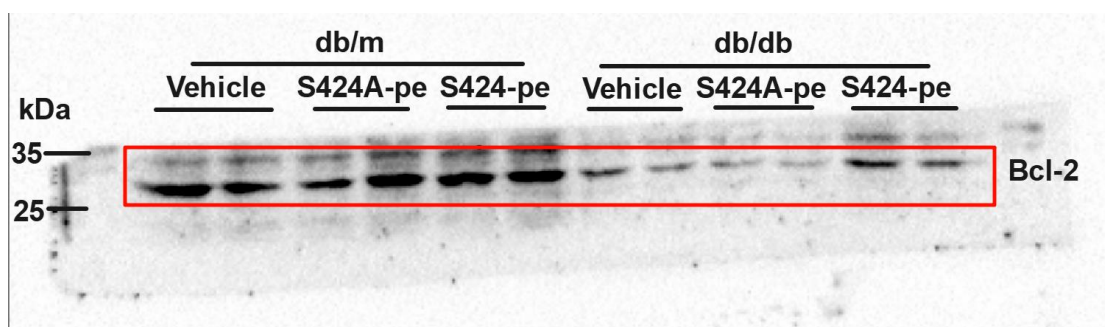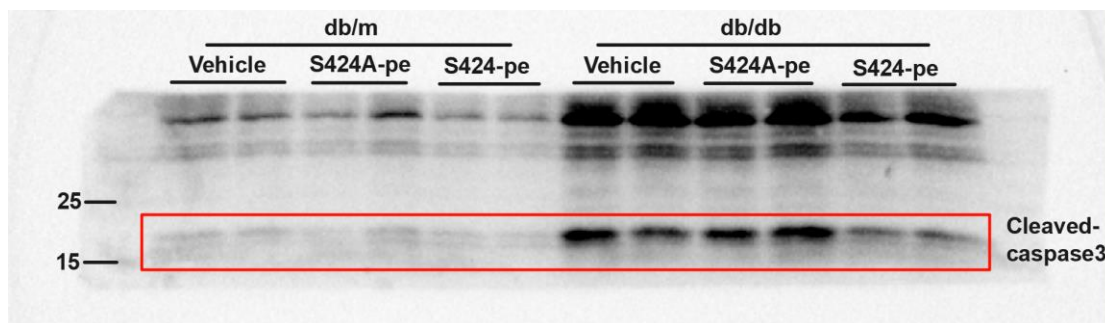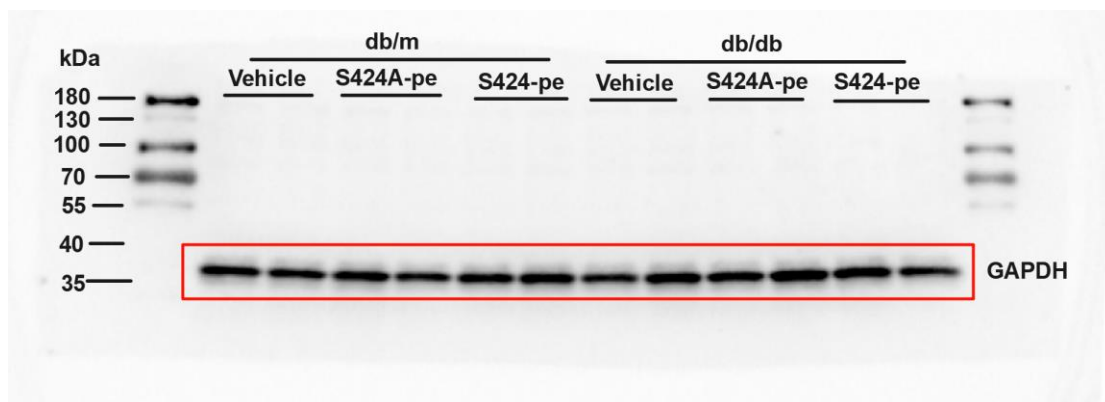

Supplement: Supplementary file 8 — Source data Fig. 6 [file 44318_2024_263_MOESM8_ESM.zip › Figure 6/6G/6G Western blot description for cropped image.pdf]

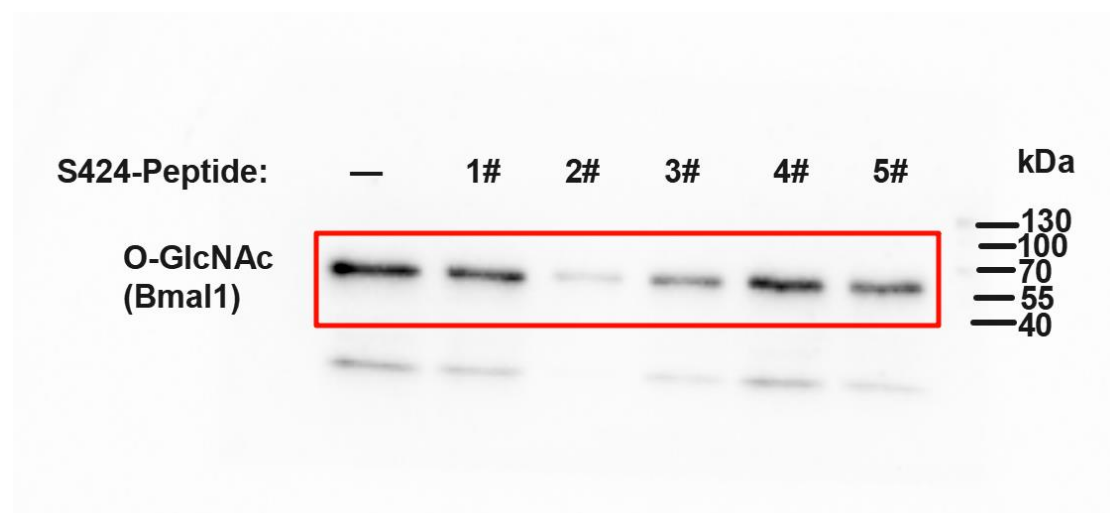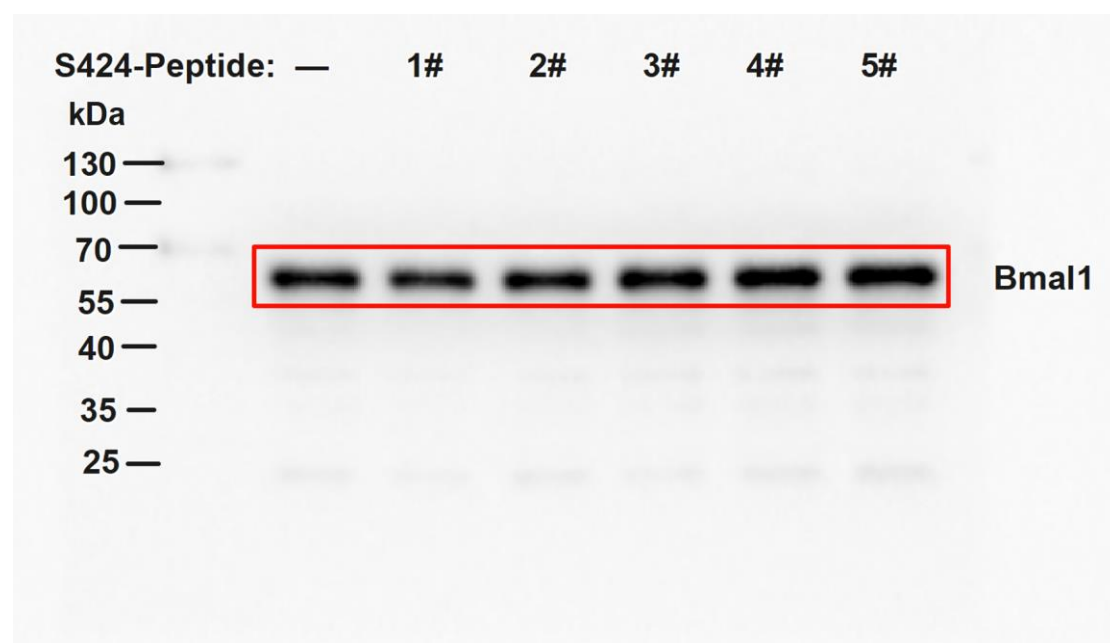

Supplement: Supplementary file 8 — Source data Fig. 6 [file 44318_2024_263_MOESM8_ESM.zip › Figure 6/6B/6B Western blot description for cropped image.pdf]

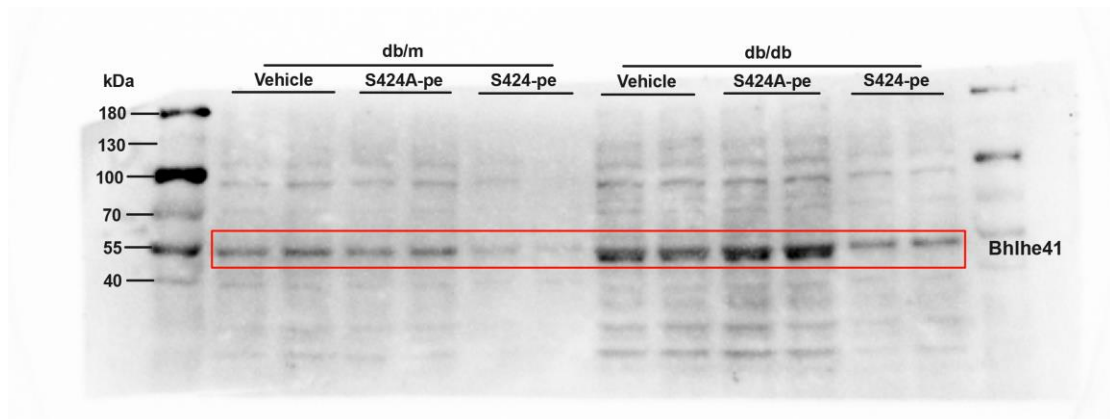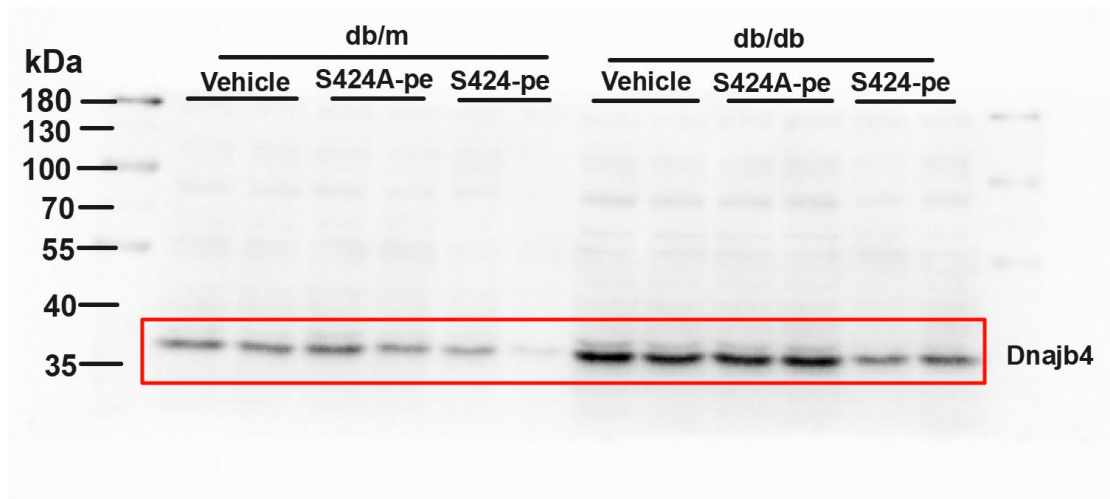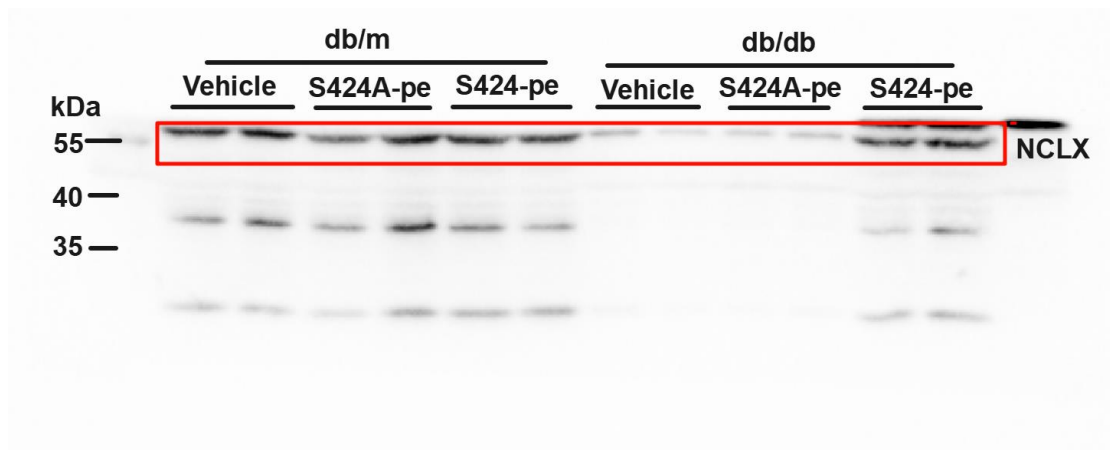

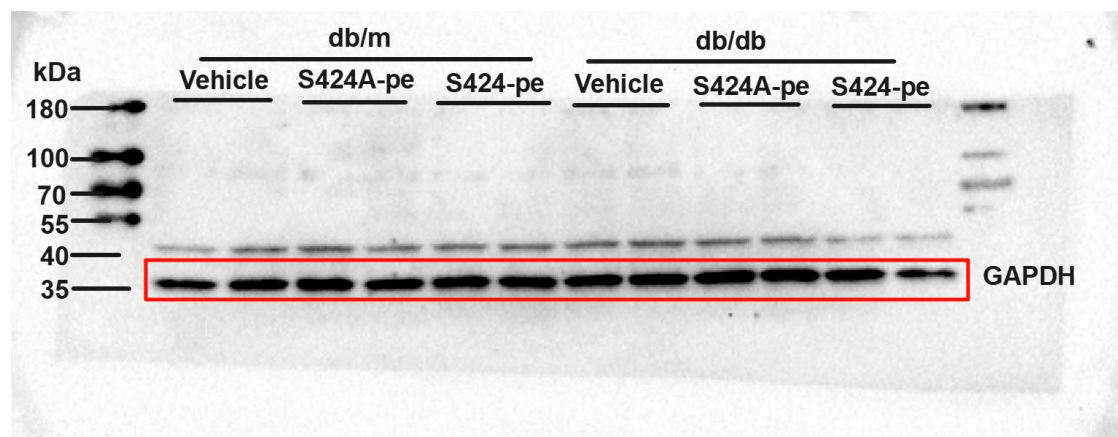

Supplement: Supplementary file 8 — Source data Fig. 6 [file 44318_2024_263_MOESM8_ESM.zip › Figure 6/6E/6E Western blot description for cropped image.pdf]

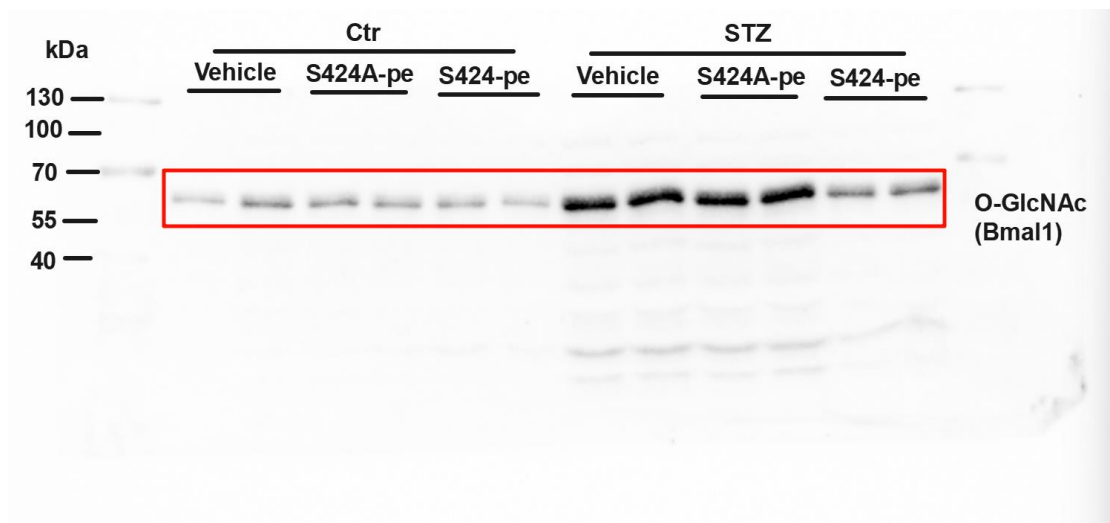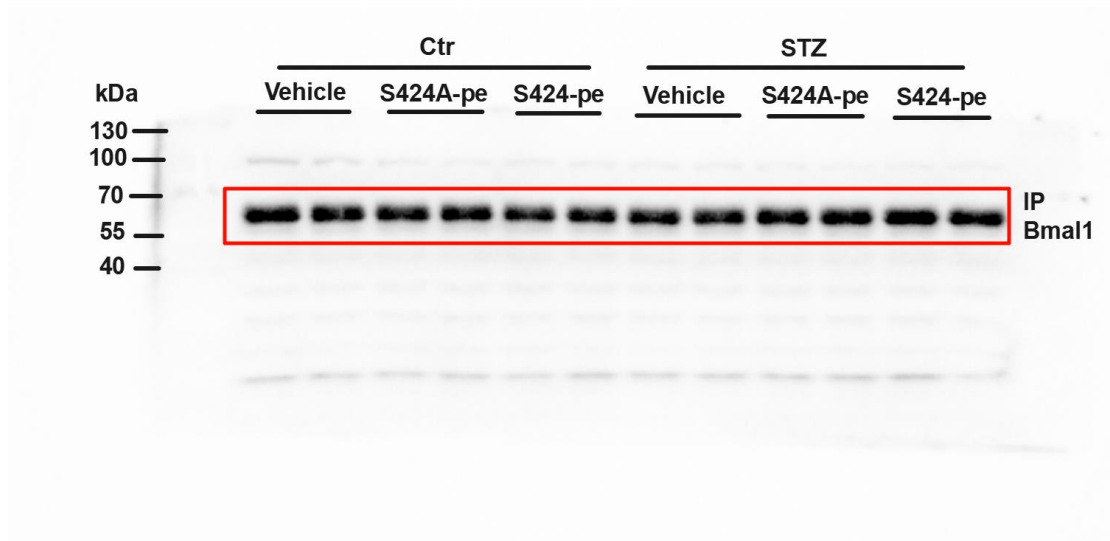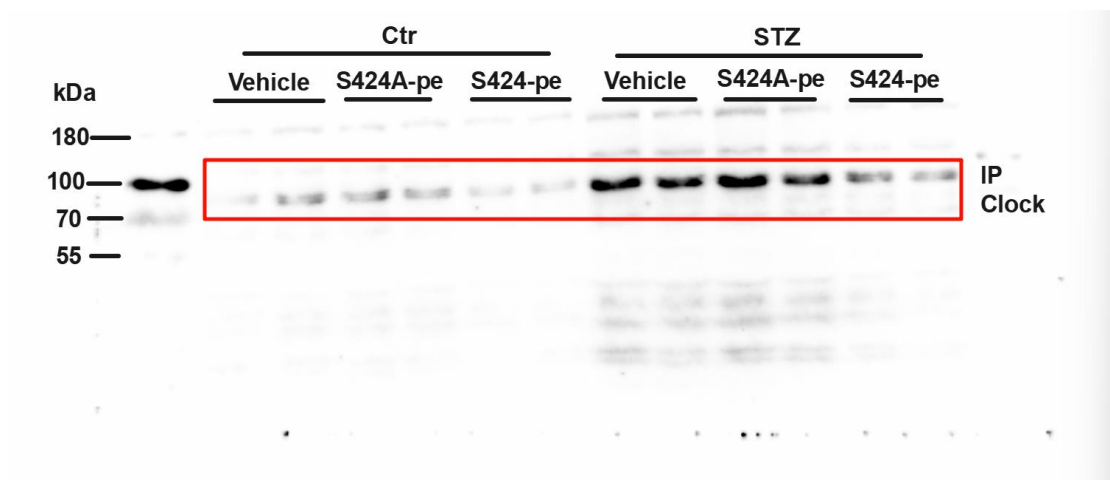

Supplement: Supplementary file 8 — Source data Fig. 6 [file 44318_2024_263_MOESM8_ESM.zip › Figure 6/6D/6D Western blot description for cropped image.pdf]

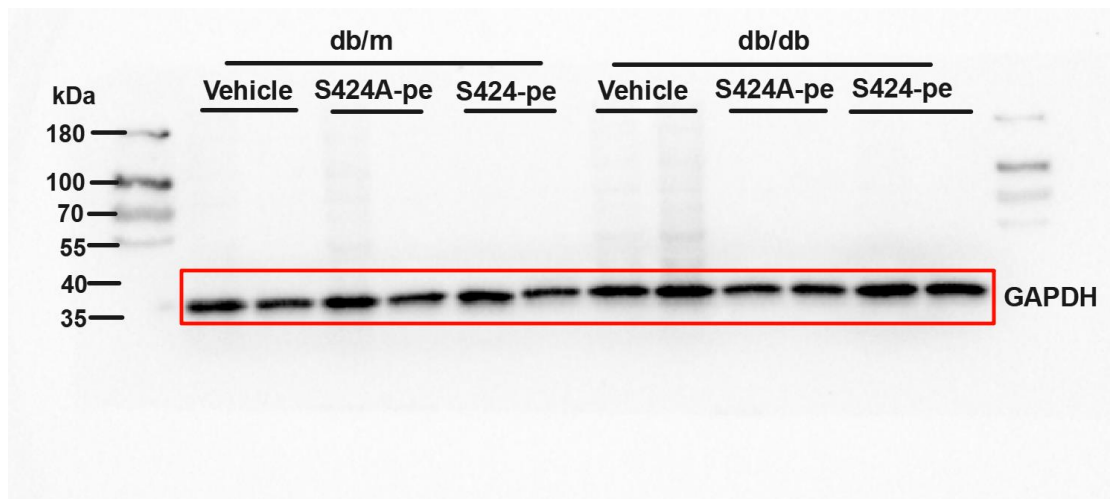

Supplement: Supplementary file 8 — Source data Fig. 6 [file 44318_2024_263_MOESM8_ESM.zip › Figure 6/6C/6C Western blot description for cropped image.pdf]
